# Genomic 5-mC contents in peripheral blood leukocytes were independent protective factors for coronary artery disease with a specific profile in different leukocyte subtypes

**DOI:** 10.1186/s13148-018-0443-x

**Published:** 2018-01-23

**Authors:** Qianyun Deng, Wei Huang, Chunyan Peng, Jiajia Gao, Zuhua Li, Xueping Qiu, Na Yang, Bifeng Yuan, Fang Zheng

**Affiliations:** 1 Center for Gene Diagnosis, Zhongnan Hospital of Wuhan University, Donghu Road 169, Wuhan, 430071 China; 2 Department of Chemistry, Key Laboratory of Analytical Chemistry for Biology and Medicine (Ministry of Education), Wuhan University, Wuhan, 430071 China; 3 Department of Laboratory Medicine, Taihe Hospital, Hubei University of Medicine, Shiyan, Hubei 442000 China

**Keywords:** Mass spectrometry, 5-Methylcytosine, 5-Hydroxymethylcytosine, Peripheral blood leukocytes, Coronary artery disease

## Abstract

**Background:**

Alterations in DNA methylation are demonstrated in atherosclerosis pathogenesis. However, changing rules of global DNA methylation and hydroxymethylation in peripheral blood leukocytes (PBLs) and different blood cell subtypes of coronary artery disease (CAD) patients are still inconclusive, and much less is known about mechanisms underlying.

**Results:**

We recruited 265 CAD patients and 270 healthy controls with genomic DNA from PBLs, of which 50 patients and 50 controls were randomly chosen with DNA from isolated neutrophils, lymphocytes and monocytes, and RNA from PBLs. Genomic 5-methylcytosine (5-mC) and 5-hydroxymethylcytosine (5-hmC) contents were quantified by liquid chromatography-electrospray ionization-tandem mass spectrometry (LC-ESI-MS/MS) assay. Genomic 5-mC contents were negatively associated with the serum total cholesterol (TC) level (*P* = 0.010), age (*P* = 0.016), and PBL classifications (*P* = 0.023), explaining 6.8% individual variation in controls. Furthermore, genomic 5-mC contents were inversely associated with an increased risk of CAD (odds ratio (OR) = 0.325, 95% confidence interval (CI) = 0.237~0.445, *P* = 2.62 × 10^− 12^), independent of PBL counts and classifications, age, sex, histories of hyperlipidemia, hypertension, and diabetes. Within-individual analysis showed a general 5-mC decrease in PBL subtypes, but significant difference was found in monocytes only (*P* = 0.001), accompanied by increased 5-hmC (*P* = 3.212 × 10^− 4^). In addition, coincident to the reduced *DNMT1* expression in patients’ PBLs, the expression level of DNMT1 was significantly lower (*P* = 0.022) in oxidized low-density lipoprotein (ox-LDL) stimulated THP-1-derived foam cells compared to THP-1 monocytes, with decreased genomic 5-mdC content (*P* = 0.038).

**Conclusions:**

Global hypomethylation of blood cells defined dominantly by the monocyte DNA hypomethylation is independently associated with the risk of CAD in Chinese Han population. The importance of monocytes in atherosclerosis pathophysiology may demonstrate via an epigenetic pathway, but prospective studies are still needed to test the causality.

**Electronic supplementary material:**

The online version of this article (10.1186/s13148-018-0443-x) contains supplementary material, which is available to authorized users.

## Background

Coronary artery disease (CAD) is the leading cause of death worldwide, counting for trillions of health care expenditure [1]. This situation will continue to deteriorate globally as risk factors continue increasing, such as hypertension, dyslipidemia, diabetes, and obesity. Despite advances in the understanding of causative portions of genetic variants, life styles, and environment factors, existing knowledge cannot fully explain the complex pathophysiology of gene-environment interactions underlying CAD. Epigenetic modification mechanisms of the genome might reveal the clue of this cooperation [2–4].

DNA methylation, methyl group added to the 5-carbon position of a cytosine (5-methylcytosine, 5-mC) within the whole genome that occur mostly at CpG sites [5], is one of the best understood epigenetic mechanisms thus far [6]. DNA methylation has been suggested to regulate gene expression and implicated in numerous biological and pathological processes including CAD [7–11]. Since a large portion of 5-mC is found in repeat sequences and transposable elements, such as long interspersed nuclear element (LINE-1) and ALU [12–14], methylation of these repetitive elements has thus been used as a surrogate for the global methylation of the genome [15]. It could be directly detected using bisulfite sequencing or other methods primarily based on the digestion of genomic DNA by restriction enzymes, like Luminometric Methylation Assay, LUMA, and the [^3^H]-methyl acceptance-based method [16, 17].

Though variations of global DNA methylation in peripheral blood leukocytes (PBLs) have been exploited in CAD, the observations remain contradictive. Baccarelli et al. found that blood LINE-1 hypomethylation was associated with baseline ischemic heart diseases and stroke in the Boston-area Normative Aging Study [18]. Haley L et al. reported LINE-1 hypomethylation was correlated to higher levels of low-density lipoprotein cholesterol (LDL-C) in a Samoan islander population [19]. In a prospective study conducted by Guarrera et al. [20], global DNA hypomethylation measured in LINE-1 repeats was associated with cardiovascular disease and myocardial infarction risk in men, being more pronounced in cases with shorter time to disease. In contrast, the association between an elevated global DNA methylation and CAD has also been observed in other studies [21, 22]. So far, these studies were based on different populations using different detection methods, part of which did not include PBL counts and classifications as probable confounders. These inconsistent findings indicated further study is necessary.

In recent years, 5-hydroxmethylcytosine (5-hmC) has been discovered as the oxidation product of 5-mC [23]. Though 5-hmC has the relatively low abundance in all cell types, it is a stable modification of genomic DNA [24]. Furthermore, it appears to be required for stem cells to maintain their pluripotency [25] and significantly reduced in many cancer types [26–28]. CAD-associated changes in global DNA hydroxymethylation are not yet known.

So, we adopted the latest liquid chromatography-electrospray ionization-tandem mass spectrometry (LC-ESI-MS/MS) method to compare global 5-mC (*n* = 535) and 5-hmC (*n* = 100) of PBLs and to evaluate intra-individual variations from different cell subtypes of PBLs between CAD patients and controls.

## Results

### Genomic 5-mC contents were decreased in cases compared with controls

Characteristics of the whole study population are presented in Table 1 (Additional file 1: Table S1–S4). The mean of 5-mC contents of CAD patients was significantly lower than controls (3.90 ± 0.63 vs 4.42 ± 0.87%, *P* = 1.10 × 10^− 14^) (Table 1).

**Table 1:** Characteristics of cases and controls in association analysis

Clinical data	Control (*n* = 270)	Case^a^ (*n* = 265)	*P*
Age (years)	57.72 ± 10	58.03 ± 9.5	0.716^b^
Male gender	150 (56%)	154 (58%)	0.601^c^
*Hypertension*	*93 (34%)*	*169 (64%)*	*< 0.001* ^c^
*Hyperlipidemia*	*63 (23%)*	*100 (38%)*	*< 0.001* ^c^
*Diabetes*	*66 (24%)*	*106 (40%)*	*< 0.001* ^c^
TC (mmol/L)	4.36 ± 0.55	4.37 ± 1.07	0.832^b,d^
*TG (mmol/L)*	*1.01 (0.77~1.28)*	*1.35 (1.00~1.92)*	*< 0.001* ^d,e^
*HDL-C (mmol/L)*	*1.26 (1.13~1.42)*	*1.10 (0.94~1.34)*	*< 0.001* ^d,e^
*LDL-C (mmol/L)*	*2.57 ± 0.36*	*2.43 ± 0.88*	*0.017* ^b,d^
*FBG (mmol/L)*	*4.71 (4.35~5.15)*	*5.84 (5.31~6.87)*	*< 0.001* ^e^
*PBLs counts (10* ^*9*^ */L)*	*5.30 (4.60~6.29)*	*6.58 (5.33~7.92)*	*< 0.001* ^e^
*PBLs classifications (PBMCs %)*	*40.31 ± 8.11*	*34.48 ± 10.16*	*< 0.001* ^b^
*5-mC contents (5-mC/C %)*	*4.42 ± 0.87*	*3.90 ± 0.63*	*< 0.001* ^b,f^

Data are presented as mean ± SD or as median (inter-quartile range). Italic letters show the significant associations and their *P* values. PBL classifications were calculated by percentages of PBMCs in PBLs

^a^The CAD patients are consists of 71 (27%) myocardial infarction and 194 (73%) angina pectoris, respectively

^b^Student’s *t* test

^c^χ^2^ test

^d^Most of the CAD patients with hyperlipidemia were under anti-hyperlipidemia therapy in the case group

^e^Mann-Whitney *U* test

^f^*P* = 1.10 × 10^− 14^

### Genomic 5-mC contents were negatively related to age, TC levels, and PBL classifications in controls

Since blood lipids, fasting blood glucose, and blood pressure are well-known established risk factors of CAD, we performed statistical analysis to evaluate correlations of DNA methylation with age, gender, TC, TG, HDL-C, LDL-C, FBG, systolic blood pressure (SBP), diastolic blood pressure (DBP), PBL counts, and classifications (peripheral blood mononuclear cells, PBMC percentages) in controls, to identify confounders in the association study between DNA methylation and CAD. We found 5-mC contents were negatively correlated with age (beta (*β*) = − 0.129, *P* = 0.034), TC (*β* = − 0.173, *P* = 0.004), and PBL classifications (*β* = − 0.163, *P* = 0.007) but positively correlated with PBL counts (*β* = 0.150, *P* = 0.014) (Table 2). However, after forward stepwise multivariate linear regression, only age (*β* = − 0.143, *P* = 0.016), TC (*β* = − 0.158, *P* = 0.010), and PBL classifications (*β* = − 0.137, *P* = 0.023) were independent factors associated with 5-mC contents, which could partially explain 6.8% individual variation.

**Table 2:** Associations of 5-mC contents with clinical characteristics in the controls

Parameters	Genomic 5-mC contents			
	Univariate associations		Multivariate associations	
	*β*	*P*	*β*	*P*
*Age*	*− 0.129*	*0.034*	*− 0.143*	*0.016*
*TC*	*− 0.173*	*0.004*	*− 0.158*	*0.010*
TG	− 0.048	0.434	–	–
HDL-C	0.008	0.894	–	–
LDL-C	− 0.045	0.460	–	–
FBG	− 0.065	0.286	–	–
SBP	0.054	0.378	–	–
DBP	− 0.007	0.902	–	–
*PBLs counts*	*0.150*	*0.014*	–	–
*PBLs classifications*	*− 0.163*	*0.007*	*−0.137*	*0.023*
*R* ^*2*^			*0.068*	–

HDL-c was sqrt-transformed. *β* stands for beta and is the standardized regression coefficient. The *β* value represents change in each parameter per SD change in 5-mC contents. Italic letters show the significant associations and their *P* values

### Genomic 5-mC contents were independent protective factors for CAD

**Fig. 1:**
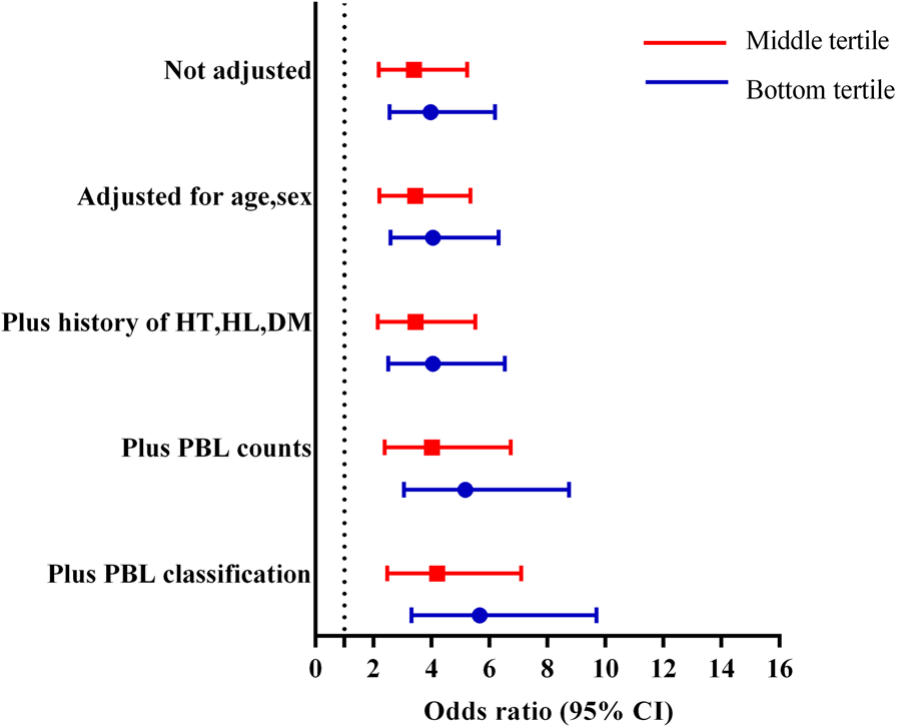
Association of global 5-mC contents tertiles with coronary artery disease risk. The top tertile was used as the reference group

Without adjustment, the OR of genomic 5-mC contents for CAD was 0.397 (95% CI 0.308~0.512, *P* = 1.15 × 10^− 12^), which indicated genomic 5-mC contents were protective factors for CAD. After progressive adjustment for various CAD risk factors especially history of hyperlipidemia and PBL counts and classifications, the association of genomic 5-mC contents with CAD remained significant (OR = 0.325, 95% CI 0.237~0.445, *P* = 2.62 × 10^− 12^) (Table 3). In analysis adjusted for age and sex, the OR for CAD was 4.044 (95% CI 2.589~6.316) in individuals with 5-mC contents in the bottom tertile compared with the top tertile (Table 4). After progressive adjustment for various risk factors, 5-mC contents remained significantly associated with CAD prevalence. The ORs for CAD progressively decreased across tertiles of 5-mC contents (Fig. 1).

**Table 3:** Associations between global 5-mC contents and CAD

	Odds ratio (95% CI)	*P* value
Not adjusted	0.397 (0.308, 0.512)	1.15 × 10^−12^
Adjusted for age and sex	0.393 (0.304, 0.507)	8.40 × 10^−13^
Plus histories of HT, HL, and DM	0.388 (0.295, 0.511)	1.66 × 10^−11^
Plus PBL counts	0.355 (0.262, 0.481)	2.54 × 10^− 11^
Plus PBL classifications	0.325 (0.237, 0.445)	2.62 × 10^−12^

The OR value represents altered risk of CAD odds per unit change in global 5-mC contents. Odds ratio less than 1 indicates that the independent factor (i.e., global 5-mC contents) is a protective factor

**Table 4:** Associations of global 5-mC contents with CAD prevalence

	Bottom tertile^a^	Middle tertile	Per SD decrease
Not adjusted	3.974 (2.552, 6.189)	3.393 (2.183, 5.237)	2.517 (1.951, 3.245)
Adjusted for age and sex	4.044 (2.589, 6.316)	3.433 (2.203, 5.349)	2.546 (1.971, 3.289)
Plus histories of HT, HL, and DM	4.048 (2.509, 6.532)	3.446 (2.153, 5.516)	2.577 (1.956, 3.395)
Plus PBL counts	5.165 (3.049, 8.750)	4.012 (2.391, 6.731)	2.816 (2.077, 3.816)
Plus PBL classifications	5.667 (3.312, 9.699)	4.195 (2.482, 7.093)	3.076 (2.245, 4.213)

Data are odds ratio (95% CI). Odds ratio more than 1 indicates that the independent factor (i.e., global 5-mC contents divided in each group) is a risk factor. The top tertile was used as the reference group. Tertile cut-points were 3.77 and 4.43%, respectively, based on global 5-mC distributions among the whole samples (i.e., 535 participants)

^a^For trend, *P* < 0.001

### DNMT1 expression and genomic 5-mC decreased in PBLs while 5-hmC elevated in monocytes in CAD

**Fig. 2:**
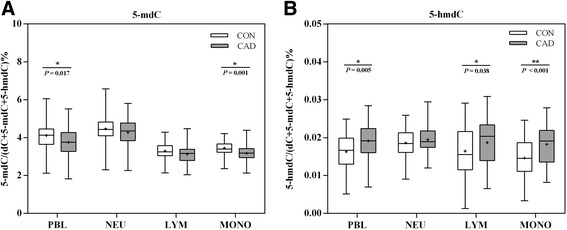
Overall distributions of 5-mC and 5-hmC in different cell types. **a** Comparison of distributions of genomic 5-mC in different cell type between controls and patients. **b** Comparison of distributions of genomic 5-hmC in different cell type between controls and patients

To further investigate the association between PBL classifications and 5-mC contents in CAD, 50 CAD patients and 50 controls were randomly selected from the whole samples. The randomly selected subjects had similar clinical and epigenetic characteristics as compared to the whole samples (Table 5, Additional file 1: Table S3–S4). Figure 2 reports the overall distribution (mean, median, and inter-quartile range) in each subtype of PBLs for the 100 selected subjects (Additional file 1: Table S5). There was a significant decrease of 5-mC (*P* = 0.017, Fig. 2a) but a statistical increase of 5-hmC (*P* = 0.005, Fig. 2b) in CAD. The overall distributions of 5-mC and 5-hmC were not significantly different among all three PBL subtypes, though the highest methylation (*P* > 0.05) and hydroxymethylation were in neutrophils while a significantly decreased 5-mC (*P* = 0.001, Fig. 2a) along with elevated 5-hmC (*P* < 0.001, Fig. 2b) in monocytes.

**Table 5:** Characteristics of case and control subgroups in PBL subtype analysis

Clinical data	Control (*n* = 50)	Case^a^ (*n* = 50)	*P*
Age (years)	64 ± 9.5	62 ± 9.9	0.248^b^
Male gender	26 (52%)	27 (54%)	0.84^c^
*Hypertension*	*16 (32%)*	*31 (62%)*	*0.003* ^c^
*Hyperlipidemia*	*9 (18%)*	*19 (38%)*	*0.026* ^c,d^
*Diabetes*	*10 (20%)*	*21 (42%)*	*0.017* ^c^
*PBLs counts (10* ^*9*^ */L)*	*5.48 (4.56~6.65)*	*6.63 (5.63~9.28)*	*0.001* ^e^
*Neutrophil (%)*	*59.22 ± 10.55*	*66.01 ± 11.45*	*0.003* ^b^
*Lymphocyte (%)*	*30.28 ± 8.46*	*24.08 ± 9.72*	*0.001* ^b^
Monocyte (%)	7.72 ± 2.78	7.47 ± 2.47	0.625^b^
*5-mC contents (5-mC/C %)*	*4.11 ± 0.73*	*3.75 ± 0.76*	*0.017* ^b^
*5-hmC contents (5-hmC/C %)*	*0.0163 ± 0.0052*	*0.0191 ± 0.0048*	*0.005* ^b^
*DNMT1 expressions*	*0.0084 ± 0.0058*	*0.0064 ± 0.0029*	*0.006* ^b^

Data are presented as mean ± SD or as median (inter-quartile range). Italic letters show the significant associations and their *P* values

^a^The CAD patients are consists of 14 (28%) myocardial infarction and 36 (72%) angina pectoris, respectively

^b^Student’s *t* test

^c^χ^2^ test

^d^Most of the CAD patients with hyperlipidemia were under anti-hyperlipidemia therapy in the case group

^e^Mann-Whitney *U* test

Table 6 shows the within-person correlations of 5-mC and 5-hmC between PBLs and PBL subtypes (neutrophils, lymphocytes, and monocytes). In controls, 5-mC contents of PBL exhibit strong associations with all three subtypes, showing Pearson correlation coefficients of 0.333 (*P* = 0.018), 0.346 (*P* = 0.014), and 0.426 (*P* = 0.002), respectively, for neutrophils, lymphocytes, and monocytes. The association remains significant in cases except the correlation between PBLs and neutrophils. The 5-hmC contents of PBLs showed no significant correlation with 5-hmC of all three PBL subtypes in controls, but was correlated with that of monocytes only (*r* = 0.454, *P* = 0.001) in cases.

**Table 6:** Within-person Pearson correlation coefficients of 5-mC and 5-hmC between PBLs and PBL subtypes

PBL subtypes			CON (*n* = 50)			CAD (*n* = 50)		
			NEU	LYM	MONO	NEU	LYM	MONO
PBL	5-mC	*r*	0.333	0.346	0.426	0.247	0.382	0.439
		*P*	*0.018*	*0.014*	*0.002*	0.084	*0.006*	*0.001*
	5-hmC	*r*	0.171	0.191	0.225	− 0.070	0.002	0.454
		*P*	0.235	0.183	0.117	0.629	0.988	*0.001*

DNA demethylation may be approached via passive demethylation by DNA methyltransferases (DNMT1, DNMT3A, and DNMT3B) or active demethylation of 5-mC, referring to the stepwise enzymatic oxidation by ten-eleven translocation (Tet) enzymes (Tet1, Tet2, and Tet3). Active and passive demethylation pathways are not mutually exclusive; thus, we analyzed the mRNA expression both of DNMTs and Tets.

DNMT1 expression significantly decreased in the CAD cohort as compared with the control (0.0064 ± 0.0029 vs 0.0084 ± 0.0058, *P* = 0.006) (Table 5). However, there was no significant difference in mRNA expressions of DNMT3A, DNMT3B, TET1, TET2, and TET3 between CAD patients and controls (Additional file 1: Figure S1).

### The 5-mC and DNMT1 expression both decreased in THP-1 foam cells

**Fig. 3:**
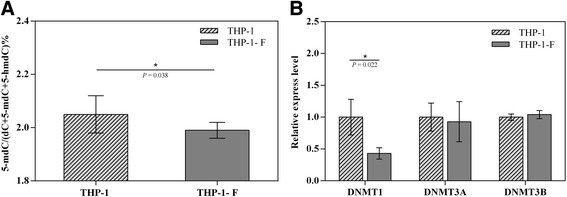
Epigenetic changes between THP-1 and THP-1 foam cells. **a** Measure contents of genomic 5-mC in THP-1 cell and THP-1-F repetitions. **b** Relative expression levels of DNMTs in THP-1 cell and THP-1-F repetitions. THP-1-F and THP-1 foam cells. The experiments for each cell line was triplicated, and the error bar indicates standard deviation

We found genomic 5-mC hypomethylation was also presented in the formation of THP-1-derived foam cells in vitro. A marginal significant decrease (*P* = 0.038) of 5-mdC contents was found in THP-1 foam cells (1.99 ± 0.007) compared with THP-1 monocytes (2.15 ± 0.07), which corresponds to a 6% decrease in the overall genomic methylation level (Fig. 3a). On a quantification assessment, DNMT1 was significantly downregulated (*P* = 0.022) while DNMT3A and DNMT3B have no change between the THP-1 monocytes and THP-1 foam cells (Fig. 3b).

## Discussion

DNA methylation plays a vital role in development, aging, and pathological process of certain diseases [29]. Early studies reported the relationship between CAD and global DNA methylation measured in LINE-1 and ALU repeats, and the results included both positive and negative correlations [18–22]. Actually, when using these repeat sequences, there is uncertainty about its comparability and the extent to which it reflects global DNA methylation contents [30]. Moreover, methods using enzyme digestion and isotope labeling or chemiluminescent, in which the methylation status of cytosine is typically assessed by reactions with sodium bisulfite modification or methylation-sensitive restriction enzymes, a complete conversion/restriction-enzymatic-reaction is difficult to be achieved [22, 31, 32]. In addition, these assays could not distinguish 5-mC from 5-hmC, and most studies did not consider the confusion induced by PBL counts and classifications [33]. So, the controversial results with different markers and methods of global DNA methylation urge us to comprehensively investigate the association between the global 5-mC contents of different blood cell sources and CAD.

Case and control study would give us some clues for association analysis, on conditions that we could exclude necessary confounders for 5-mC content detection. Hence, we compared global 5-mC contents between CAD cases and controls. Importantly, global 5-mC contents were significantly decreased in cases. However, DNA methylation is not only a heritable epigenetic element but also a dynamic process related to demographical, clinical, and environmental factors. Therefore, we need to survey controls for defining the confounding factors in DNA methylation analysis. Interestingly, in controls, after forward stepwise multivariate linear regression, only age, TC, and PBL classifications were independent factors associated with 5-mC contents, which could partially explain 6.8% individual variation. Thus, age, TC, and PBL classification were identified as important cofounders for case-control study of 5-mC contents. Then, after these confounders were adjusted one by one in unconditional logistic regression, we could conclude 5-mC contents as an independent protective factor for CAD. Moreover, the reverse correlation between risk factors (age, TC, PBL classification) and 5-mC contents was coincident with the decrease of 5-mC contents as a protective factor in CAD cases.

Actually, the longitudinal decline in genome methylation over time has been reported [14], and DNA methylation biomarkers showed high correlation with chronological age and with age-acceleration effects associated with pathological conditions, morbidity, and mortality [34, 35]. Accumulated data supported the idea that DNA methylation showed reduced stringency in maintenance over the lifespan, resulting in an increase in inter-individual variability along with the overall decrease in DNA methylation [36]. Our previous study [37] also observed a slight negative correlation between 5-mC and chronological age.

Meanwhile, the heterogeneous composition of different blood cells within whole blood samples also has an influence on the determined global methylation [38–41]. But whether the variation DNA methylation was the result of the PBL classification, or the cause, or the company, need further investigation. On the other aspect, people more and more realize the important role of circulating immune cells in the inflammation process of atherosclerosis [42]. Not only the cell amount but also the cell classification that is more important decides the outcome of CAD [39]. Therefore, associations of global methylation patterns with certain health-associated conditions can partly be the results of a cell composition effect. Thus, the PBLs were divided into three categories: neutrophils, lymphocytes, and monocytes. Then, we analyzed the 5-mC contents separately to confirm that the changed DNA methylation between cases and controls was not only due to altered PBL classifications. Subgroup analysis results were in accordance to and confirmed the previous finding in groups, while the 5-mC contents of PBLs were significantly decreased in CAD and correlated with those of neutrophils, lymphocytes, and monocytes in controls. However, the correlation of global DNA methylation between PBLs and neutrophils vanished in CAD subgroup, even with a significant increase of neutrophil percentage. But significant correlations of global DNA methylation between PBLs and monocytes were present with the most dominant reduction in global DNA methylation of patients’ monocytes. Based on this result, combining the negative correlation between TC on 5-mC in 270 controls, we hypothesized that monocytes in CAD might be more susceptible to exposures of CAD risk factors (i.e., TC) in epigenetic pathway, compared to other blood cell subtypes. Neutrophils may respond to these exposures via alternative ways [43], such as increased amount.

Recently, it is reported that circulating immune cells can be primed by external stimuli, such as lipid particles, one of the most differences in blood between CAD cases and controls, to obtain a long-term epigenetic memory [44]. A study of the TG-lowering drug fenofibrate suggested that a 3-week daily treatment was not sufficient to reverse lipid-associated DNA methylation changes despite the short half-life of various blood cell types [45]. This suggests these changes did not arise in the circulation but occurred already in hematopoietic stem cells, where lipid priming has previously been started. We thought that monocytes might have been epigenetically altered in microenvironment with CAD risk factors, even before they migrated and settled under endothelia, and further facilitated the CAD process.

Migration of monocytes and formation of lipid-laden macrophage is the trigger of atherosclerosis plaque [38]. However, we were not able to gather the corresponding tissue samples in plaque lesions in CAD patients. Thus, in vitro cell model provides a proper alternative. As a classic model system for investigating the possible molecular mechanisms in foam cell development, THP-1 human monocyte cell line was used as the in vitro model. In agreement with the decreased global 5-mdC contents in CAD patients, lower genomic DNA methylation was also found in THP-1-derived foam cells compared to THP-1 monocytes, which suggests demethylation process might be involved into the foam stage of atheromatous plaque. The lower global 5-mC contents in monocytes of CAD patients, together with a decrease in the process of THP-1 foam cell formation, might imply a persistent decline in monocytes/macrophage during progression of atherosclerosis. Although our observational cross-section study could not infer causal directions, we could conclude monocytes with decreased 5-mC as a character of atherosclerosis pathology, and speculate monocytes were prone to external stimulus and differentiated hereafter to carry a reduced 5-mC. Further investigation is indispensable. In coincidence with previous reports, we also found the reduced global DNA methylation was accompanied with decreased expression of DNMT1 [46, 47].

Apart from the significant global hypomethylation found in monocytes of CAD patients and in vitro monocyte cell line model, DNA methylation alterations in specific genes in monocyte/macrophage in atherosclerosis have also been reported. Using dietary inducing hypercholesterolemia, mice can lead a hypomethylation state in spleen focus-forming virus proviral integration oncogene (PU.1) and interferon regulatory factor 8 (IRF8) and consequently resulted in a phenotypic alteration in monocytes and macrophages [48]. Monocyte chemotactic protein-1 (MCP-1), related with the migration and gather of monocytes, showed an overexpression due to the DNA hypomethylation induced by homocysteine, which is mediated by NF-κB/DNMT1 pathway [49]. A recently longitudinal study [50] showed ARID5B (transcription coactivator for histone H3K9me2 demethylation) CpG methylation is inversely associated with both ARID5B expression and atherosclerosis in human CD14+ blood monocyte, while the associations vanished in T cell samples from the same subset. These data suggest that whether in the causal or reflective pathway of disease, epigenome features in circulating monocytes are potential “biosensors” that might be useful for detecting early signs of metabolic disorders and elevated CAD risk.

In addition, we found 5-hmC levels are also highly heterogeneous in PBL subtypes (Table 6). 5-hmC is less abundant than 5-mC and is present at higher levels in neurons than in other cell types. Quantification of bulk levels of 5-hmC found that it is relatively rare, with levels varying by tissue from < 0.1 to 0.7% of cytosines globally [51]. The ratio of 5-hmC vs 5-mC is a cell-type specific, and the dynamics of generation and maintenance of 5-mC hydroxylation might also affect 5-mC levels. In PBLs and monocytes, elevated 5-hmC along with decreased 5-mC was found in CAD compared to controls (Fig. 2). It is reasonable because 5-hmC is the oxidized product of 5-mC, which is an intermediate product in DNA demethylation. Additionally, it is widely reported that the levels of 5-hmC inversely correlate with the rate of proliferation [52], and when the abundance and distribution of 5-hmC change, diseases occur. However, what kind of “epigenetic signal” 5-hmC is and how it relates to the 5-mC signal remain to be the main challenges that need to be addressed to date.

Nevertheless, the present study still has some limitations. First, there is significant evidence that many genetic, demographical, clinical, and environmental factors are strong cofounding variables. However, these underappreciated confounding variables all contribute to the overall measured phenotype. Within our present study, only age, sex, history of hypertension, hyperlipidemia, diabetes, blood lipids, fasting blood glucose, PBL counts, and classifications had been taken into account, and data such as smoking habits and BMI failed to be collected, which was one of our limitations. Second, even though we have isolated different subtypes of PBLs to detect 5-mC contents of each subtype and confirmed the association within each PBL subtype in vivo experiments, the sample size is still relatively small. Third, since it was a cross-sectional study, this investigation could not observe in prospective way and thus could not identify the causal effect, and longitudinal studies are more convinced to confirm the temporal relation of epigenetic changes in blood cells in the progress of CAD and to prove TC as causes. Thus, prospective studies are really required for further study.

## Conclusions

In summary, the measurement of 5-mC contents by LC-ESI-MS/MS system in a relatively large sample for epigenetic study demonstrated that a significant reduction of 5-mC contents was the independent risk factor of CAD from the molecular epidemiology aspect. The decreased methylation level during the foam cell formation in THP-1 cells and in monocyte subtypes of CAD supported that DNA hypomethylation in PBLs could be a contributor to the pathogenesis of CAD. These suggested a possible epigenetic pathway of monocytes involved in CAD pathogenesis.

## Methods

### Chemicals and reagents

The 2′-deoxycytidine (dC), 2′-deoxyguanosine (dG), 2′-deoxyadenosine, thymidine (T), cytidine (rC), guanosine (rG), adenosine (rA), uridine (rU), 5-methyl-2′-deoxycytidine (5-mdC), and 5-hydroxymethyl-2′-deoxycytidine (5-hmdC) were purchased from Sigma-Aldrich (Beijing, China). Phosphodiesterase I was from Sigma-Aldrich (St. Louis, MO, USA). S1 nuclease and alkaline phosphatase (CIAP) were purchased from Takara Biotechnology Co., Ltd. (Dalian, China). Chloroform and formic acid were purchased from Sinopharm Chemical Reagent Co., Ltd. (Shanghai, China), and chromatographic grade methanol was from Merck (Darmstadt, Germany). Water used throughout the study was purified by a Milli-Q apparatus (Millipore, Bedford, MA). All other reagents were obtained from various commercial sources and were of analytical grade unless otherwise indicated.

### Subjects

This case-control study was approved by the Medical Ethics Committee of Zhongnan Hospital of Wuhan University (approval number 2010052), and written informed consent were obtained from all participants prior to enter the study. A total of 265 patients with CAD and 270 control subjects were collected in Zhongnan Hospital, Wuhan University, Hubei, China, from March 2012 to April 2016, based on the criterion as previous reports [53]. All participants were unrelated individuals from the Chinese Han population. To verify the different methylation level of different cell subsets, 50 CAD patients and 50 healthy controls were randomly selected for cell isolation and mRNA expression research. Briefly, this study enrolled CAD patients with ≥ 70% stenosis of one major coronary artery, or ≥ 50% of the left main coronary artery, which was confirmed by coronary angiography. Exclusion criteria included acute heart failure, congenital heart disease, myocardial bridge or cardiomyopathy, coronary artery spasm, severe non-coronary cardiovascular diseases, systemic infections or inflammatory diseases, and the use of immunosuppressant or chemotherapeutic agents. Healthy individuals and patients with a normal angiogram were selected as controls (non-CAD). All control participants showed no signs of abovementioned cardiac or systemic diseases based on physical examination records of enrollment. Concentrations of fasting blood glucose (FBG), total cholesterol (TC), total triglyceride (TG), high-density lipoprotein cholesterol (HDL-C), and low-density lipoprotein cholesterol (LDL-C) were detected by standard techniques, which were employed by the Core Laboratory in Zhongnan Hospital. Relevant data were collected from all the participants by interviews or from medical case files.

### Isolation of cell subsets

Fresh peripheral blood was taken from participants into a 10-ml sterile EDTA tubes, 1 ml blood was used for DNA and RNA extraction, respectively, and others were used to isolate cell subsets. Peripheral blood mononuclear cells (PBMCs) were isolated by centrifugation in Ficoll/Hypaque 1.077 g/ml (Sigma Chemical Co., Munich, Germany) and washed twice with phosphate buffered saline (PBS, Sigma). Monocytes were purified from PBMCs using positive selection in a magnetic-activated cell sorted (MACS) system (Miltenyi Biotech, Bergisch Gladbach, Germany) with CD14 microbeads (Miltenyi) according to the manufacturer instructions. The depleted cells were also collected as lymphocyte rich set. The dense layer of cells from the 1.077 g/ml Ficoll/Hypaque separation, which contains mainly neutrophils and erythrocytes, was lysed twice using an ammonium chloride buffer to remove erythrocytes. Successful isolation of monocytes was confirmed with FACS using conjugated antibodies (M5E2, BD Biosciences). The monocyte purity was on average 95% (Additional file 1; Figure S2).

### DNA extraction and enzymatic digestion

Total DNA was isolated from whole blood and separated blood subtypes using a Qiagen DNeasy Blood & Tissue Kit following the manufacturer instructions. After extraction, DNA was quantified by NanoDrop (Thermo Scientific NanoDrop products, Wilmington, DE). The isolated genomic DNA was enzymatically digested according to previously described method. Briefly, DNA (3 μg) was first denatured by heating at 95 °C for 5 min and then chilling on ice for 2 min. Then, 1/10 volume of S1 nuclease buffer (30 mM CH_3_COONa, pH 4.6, 280 mM NaCl, 1 mM ZnSO_4_) and 100 units of S1 nuclease were added before the mixture (20 μL) was incubated at 37 °C for 16 h. Subsequently, after 1/10 volume of alkaline phosphatase buffer (50 mM Tris-HCl, 10 mM MgCl_2_, pH 9.0), 0.002 units of venom phosphodiesterase I, and 10 units of alkaline phosphatase were added, the solution was incubated at 37 °C for an additional 4 h followed by extraction with an equal volume of chloroform for twice. The aqueous layer was collected and lyophilized to dryness and then reconstituted in 100 μL water. About 30 μL of the obtained samples were then subjected to liquid chromatography-electrospray ionization-tandem mass spectrometry (LC-ESI-MS/MS) analysis.

### LC-ESI-MS/MS analysis

As stated in the previous research [54], analysis of the samples was performed on the LC-ESI-MS/MS system consisting of an AB 3200 QTRAP mass spectrometer (Applied Biosystems, Foster City, CA, USA) with an electrospray ionization source (Turbo Ionspray) and a Shimadzu LC-20ADHPLC (Tokyo, Japan) with two LC-20AD pumps, a SIL-20A autosampler, a CTO-20AC thermostatted column compartment, and a DGU-20A3 degasser. Data acquisition and processing were performed using AB SCIEX Analyst 1.5 Software. The HPLC separation was performed on a Hisep C18-T column (150 mm × 2.1 mm i.d., 5 μm, Weltech Co., Ltd., Wuhan, China) with a flow rate of 0.2 mL/min at 35 °C. Formic acid in water (0.1%, *v*/*v*, solvent A) and formic acid in methanol (0.1% *v*/*v*, solvent B) were employed as mobile phase. A gradient of 5 min 5% B, 10 min 5–30% B, 5 min 30–50% B, 3 min 50–5% B, and 17 min 5% B was used.

The mass spectrometry detection was performed under positive electrospray ionization mode [37]. The target nucleosides were monitored by multiple reaction monitoring (MRM) mode using the mass transitions (precursor ions ➔ product ions) of dC (228.4 ➔ 112.2), T (243.3 ➔ 127.2), dA (252.4 ➔ 136.2), dG (268.4 ➔ 152.4), rC (244.4 ➔ 112.2), rU (245.4 ➔ 113.1), rA (268.4 ➔ 136.2), rG (284.5 ➔ 152.2), 5-mdC (242.3 ➔ 126.1), and 5-hmdC (258.2 ➔ 142.1). The ions for nucleosides were listed in Additional file 1: Table S6. The MRM parameters of all nucleosides were optimized to achieve maximal detection sensitivity.

Genomic 5-mC and 5-hmC were calculated using the following formula we had described in our previous study.

$$ 5-\mathrm{mC},\%\left(\mathrm{or}\kern0.5em 5-\mathrm{hmC},\%\right)=\frac{{\mathrm{M}}_{5-\mathrm{mdC}}\left(\mathrm{or}\kern0.50em {\mathrm{M}}_{5-\mathrm{hmdC}}\right)}{{\mathrm{M}}_{\mathrm{dC}}+{\mathrm{M}}_{5-\mathrm{mdC}}+{\mathrm{M}}_{5-\mathrm{hmC}}}\times 100\% $$

M_5-mdc_, M_5-hmdC_, and M_dC_ are the molar quantities of 5-mC, 5-hmC, and C, respectively, determined in DNA samples.

Method validation was done with synthesized 5-mC or 5-hmC containing oligodeoxynucleotide by comparing the measured 5-mdC or 5-hmdC contents to the theoretical 5-mdC or 5-hmdC contents. The results showed that good accuracy could be achieved, which are manifested by relative errors being − 5.0–11.0% (Additional file 1: Table S7). The reproducibility of the method was evaluated by the measurement of intra- and inter-day imprecisions (Additional file 1: Table S8). These results indicated that the online trapping/LC-ESI-MS/MS method was reliable for the simultaneous quantification of 5-mC and 5-hmC in genomic DNA.

### RNA extraction and quantification real-time PCR

Total RNA was isolated using RNeasy Plus Mini Kit (Qiagen, Hilden, Germany) according to the manufacturer instructions, and cDNA synthesis was performed with Revert Aid First Strand cDNA Synthesis Kit (Thermo Scientific Corp., Lithuania). Five nanograms of cDNA were used for real-time reaction using Fast SYBR® Green PCR Master Mix-PE (Applied Biosystem) and Bio-RAD CFX 96 real-time system (Bio-Rad Laboratories (Shanghai) Co., Ltd.). Relative gene expression levels were calculated using the comparative crossing threshold method of relative quantification (△Cq) and fold change (FC) values. △Cq was designated as the mean Cq (mean of duplicates) of a target gene subtracted by the mean Cq (mean of duplicates) of a reference gene (*GAPDH*). Based on the recommendations from the manufacturer, Cq expression cutoff was set to 30, which was applied for calculation. For comparison of mean expression levels between the CAD group and the control group, FC was designated as 2^−△Cq^. The detailed primer information and qPCR data processing were shown in (Additional file 1: Table S9, Figure S3).

### Statistical analysis

Continuous variables were expressed as mean ± SD or as median (inter-quartile range), as appropriate, based on distributions. Continuous variables were compared by student’s *t* test or Mann-Whitney *U* test and categorical variables by chi-squared test. The correlations between DNA methylation and age, sex, hypertension, hyperlipidemia type 2 diabetes, PBL counts, and classifications were analyzed by univariate regression and multivariate regression. For multivariate regression, genomic 5-mC content was used as dependent variable, and age, TC, PBL counts, and PBL classifications were used as independent variable. Associations between global 5-mC contents and CAD were analyzed by multivariate logistic regression. Associations of global 5-mC contents with CAD prevalence were analyzed by unconditional logistic regression, progressively adjusted for age, sex, history of hypertension, hyperlipidemia, diabetes, PBL counts, and PBL classifications. We characterized the shapes of the associations by the calculated ORs using tertiles of global 5-mC contents in all 535 participants. We used Pearson correlation coefficients to estimate the within-person correlation by source of DNA. All statistical tests were two-sided, and the level of statistical significance was set at *P* < 0.05. All analyses were performed in SPSS version 17.0 (SPSS Inc., Chicago, USA).

## Additional file

Additional file 1: Table S1. Clinical characteristics and measure contents of 5-mdC in genomic DNA of blood from 220 healthy controls. **Table S2.** Clinical characteristics and measure contents of 5-mdC in genomic DNA of blood from 215 CAD patients. **Table S3.** Clinical characteristics of 50 healthy controls in the randomly selected subgroup. **Table S4.** Clinical characteristics of 50 CAD patients in the randomly selected subgroup. **Table S5.** Measured contents of 5-mdC and 5-hmdC in genomic DNA of blood samples in subgroup with different blood cell sources. **Table S6.** The qualitative and quantitative ions for the detection of nucleosides. **Table S7.** Accuracy of the method for the detection of 5-mdC and 5-hmdC. **Table S8.** Intra- and inter-day imprecision for the quantification of 5-mdC and 5-hmdC by LC-ESI-MS/MS method. **Table S9.** Primers used for qPCR. **Figure S1.** The mRNA expression for DNMT1, DNMT3A, DNMT3B, TET1, TET2, and TET3 in the subgroup. **Figure S2.** Confirmation of the isolated monocytes with FACS. **Figure S3.** The standard curves, amplification plots, and melting peaks of qPCR. (DOCX 2049 kb)

## Abbreviations

5-hmC: 5-Hydroxymethylcytosine
5-hmdC: 5-Hydroxymethyl-2’deoxycytidine
5-mC: 5-Methylcytosine
5-mdC: 5-Methyl-2’deoxycytidine
ARID5B: Transcription coactivator for histone H3K9me2 demethylation
CAD: Coronary artery disease
CI: Confidence interval
DBP: Diastolic blood pressure
DM: Diabetes mellitus
DNMTs: DNA methyltransferases
FBG: Fasting blood glucose
GAPDH: Glyceraldehyde 3-phosphate dehydrogenase
HDL-C: High-density lipoprotein cholesterol
HL: Hyperlipidemia
HT: Hypertension
IRF8: Interferon regulatory factor 8
LC-ESI-MS/MS: Liquid chromatography-electrospray ionization-tandem mass spectrometry
LDL-C: Low-density lipoprotein cholesterol
LINE-1: Long interspersed nuclear element-1
LYM: Lymphocyte
MCP-1: Monocyte chemotactic protein-1
MONO: Monocyte
MRM: Multiple reaction monitoring
NEU: Neutrophil
OR: Odds ratio
ox-LDL: Oxidized low-density lipoprotein
PBLs: Peripheral blood leukocytes
PBMCs: Peripheral blood mononuclear cells
PU.1: Spleen focus-forming virus proviral integration oncogene
qPCR: Quantification real-time PCR
SBP: Systolic blood pressure
TC: Total cholesterol
TET: Ten-eleven translocation
TG: Total triglyceride
THP-1: Human acute monocytic leukemia cell line
