# Supplementary material for: Genomic 5-mC contents in peripheral blood leukocytes were independent protective factors for coronary artery disease with a specific profile in different leukocyte subtypes
Source: Clin Epigenetics. 2018 Jan 23;10:9. doi: 10.1186/s13148-018-0443-x (PMC5782379; doi:10.1186/s13148-018-0443-x)

Supporting Information

for

**Genomic 5-mC Contents in Peripheral Blood Leukocytes Were Independent Protective Factors for Coronary Artery Disease with a Specific Profile in Different Leukocyte Subtypes**

Qianyun Deng,^a, 1^ Wei Huang,^b, 1^ Chunyan Peng,^a, c, 1,^ Jiajia Gao,^a^ Zuhua Li,^a^ Xueping Qiu,^a^ Na Yang,^a^ Bifeng Yuan,^b^ * and Fang Zheng^a^ *

^a^ Center for Gene Diagnosis, Zhongnan Hospital of Wuhan University, Donghu Road 169, Wuhan, 430071, China.

^b^ Key Laboratory of Analytical Chemistry for Biology and Medicine (Ministry of Education), Department of Chemistry, Wuhan University, Wuhan, China

^c^ Department of Laboratory Medicine, Taihe Hospital, Hubei University of Medicine, Shiyan 442000, Hubei, China

^1^ These authors contributed equally to this work.

*** Corresponding authors. Center for Gene Diagnosis, Zhongnan Hospital of Wuhan University, Donghu Road 169, 430071, Wuhan, China.

E-mail addresses: [zhengfang@whu.edu.cn](mailto:zhengfang@whu.edu.cn) (F. Zheng) and [bfyuan@whu.edu.cn](mailto:bfyuan@whu.edu.cn) (B. Yuan)

Running Title: Contents of genomic 5-mdC are correlated with CA

**Table S1.** Clinical characteristics and measure contents of 5-mdC in genomic DNA of blood from 220 healthy controls.

| Sample ID | Gender^a^ | Age | 5-mdC, % | HT^b^ | HL^b^ | DM^b^ | TC | TG | HLL-c | LDL-c | FBG | SBP | DBP | CBC | | | |
| --- | --- | --- | --- | --- | --- | --- | --- | --- | --- | --- | --- | --- | --- | --- | --- | --- | --- |
|  |  |  |  |  |  |  | (mmol/L) | | | | | (mm Hg) | | PBL(10^9^/L) | NEU% | LYM% | MONO% |
| N137 | 2 | 41 | 6.37 ± 0.611 | 1 | 0 | 1 | 4.24 | 0.98 | 1.19 | 2.49 | 5.38 | 94 | 59 | 6.27 | 58.4 | 33.8 | 5.9 |
| N146 | 2 | 35 | 4.86 ± 0.010 | 0 | 1 | 0 | 3.89 | 0.56 | 1.19 | 2.46 | 4.48 | 130 | 82 | 5.57 | 63.7 | 33.0 | 2.0 |
| N152 | 2 | 54 | 4.66 ± 0.112 | 1 | 0 | 1 | 4.31 | 1.6 | 1.18 | 2.52 | 6.13 | 96 | 65 | 6.88 | 59.3 | 26.9 | 11.3 |
| N153 | 1 | 68 | 2.16 ± 0.001 | 1 | 0 | 1 | 4.85 | 1.32 | 1.94 | 2.12 | 4.48 | 103 | 81 | 4.78 | 59.4 | 29.7 | 10.5 |
| N160 | 2 | 28 | 4.83 ± 0.484 | 0 | 1 | 0 | 4.91 | 0.83 | 1.49 | 2.83 | 4.38 | 121 | 83 | 6.29 | 69.8 | 21.9 | 5.4 |
| N162 | 1 | 66 | 3.57 ± 0.175 | 1 | 1 | 0 | 4.9 | 1.6 | 1.18 | 2.88 | 5.6 | 101 | 56 | 6.04 | 34.6 | 58.6 | 2.3 |
| N177 | 1 | 59 | 4.60 ± 0.242 | 0 | 0 | 0 | 3.99 | 1.35 | 1 | 2.64 | 5.54 | 119 | 75 | 7.57 | 51.3 | 39.2 | 7.0 |
| N182 | 2 | 34 | 5.32 ± 0.088 | 0 | 0 | 1 | 5.19 | 0.64 | 1.82 | 2.37 | 5.84 | 151 | 78 | 7.08 | 71.7 | 26.6 | 1.3 |
| N201 | 1 | 38 | 4.36 ± 0.231 | 1 | 0 | 1 | 4.94 | 1.53 | 1.24 | 2.96 | 4.77 | 139 | 87 | 6.65 | 48.2 | 43.9 | 3.5 |
| N206 | 1 | 58 | 3.72 ± 0.281 | 0 | 0 | 1 | 4.39 | 0.91 | 1.42 | 2.86 | 5.31 | 104 | 63 | 3.41 | 58.9 | 39.0 | 0.3 |
| N210 | 1 | 85 | 5.44 ± 0.060 | 1 | 0 | 0 | 4.21 | 0.91 | 1.12 | 2.62 | 5.25 | 128 | 60 | 5.51 | 56.3 | 31.4 | 5.8 |
| N213 | 2 | 52 | 4.51 ± 0.065 | 0 | 0 | 0 | 5.11 | 1.63 | 1.29 | 2.97 | 5.21 | 134 | 70 | 5.21 | 64.8 | 31.9 | 2.5 |
| N219 | 2 | 50 | 5.33 ± 0.002 | 0 | 1 | 1 | 4.65 | 1.74 | 1.34 | 2.74 | 4.56 | 101 | 74 | 8.36 | 77.2 | 18.7 | 2.4 |
| N226 | 2 | 55 | 4.83 ± 0.295 | 1 | 0 | 0 | 4.82 | 1.01 | 1.46 | 2.85 | 4.64 | 99 | 56 | 7.48 | 62.9 | 30.9 | 5.1 |
| N229 | 1 | 64 | 5.60 ± 0.056 | 1 | 1 | 0 | 3.83 | 0.63 | 1.24 | 2.33 | 5.26 | 105 | 85 | 5.15 | 54.6 | 28.5 | 14.2 |
| N293 | 1 | 70 | 5.18 ± 0.116 | 0 | 0 | 1 | 4.56 | 0.77 | 1.65 | 2.36 | 3.95 | 157 | 80 | 8.46 | 66.5 | 21.2 | 9.5 |
| N297 | 1 | 66 | 4.93 ± 0.011 | 0 | 0 | 0 | 4.69 | 1.07 | 0.98 | 3.03 | 5 | 131 | 88 | 7.19 | 75.1 | 16.8 | 7.1 |
| N298 | 2 | 49 | 4,61 ± 0.192 | 1 | 1 | 0 | 4.56 | 1.23 | 1.16 | 2.65 | 4.05 | 112 | 71 | 6.54 | 62.3 | 27.8 | 7.5 |
| N299 | 1 | 57 | 1.80 ± 0.057 | 1 | 0 | 1 | 4.23 | 1.09 | 1.18 | 2.58 | 5.62 | 120 | 76 | 4.74 | 64.8 | 27.2 | 6.5 |
| N300 | 1 | 53 | 4.65 ± 0.038 | 0 | 0 | 0 | 4.79 | 0.63 | 1.54 | 2.63 | 4.11 | 140 | 80 | 5.62 | 59.5 | 27.8 | 7.7 |
| N301 | 2 | 60 | 4.00 ± 0.166 | 0 | 0 | 1 | 4.35 | 1.08 | 1.05 | 2.71 | 4.23 | 120 | 70 | 3.36 | 52.4 | 34.5 | 12.2 |
| N302 | 2 | 54 | 5.26 ± 0.284 | 1 | 0 | 0 | 4.44 | 0.7 | 1.21 | 2.71 | 4.45 | 108 | 68 | 5.7 | 56.1 | 36.0 | 6.7 |
| N305 | 1 | 39 | 2.91 ± 0.049 | 1 | 1 | 1 | 4.3 | 1.07 | 1.06 | 1.64 | 4.43 | 120 | 80 | 4.58 | 64.4 | 27.7 | 7.0 |
| N306 | 1 | 67 | 5.02 ± 0.146 | 0 | 0 | 0 | 3.79 | 1.03 | 1.02 | 2.3 | 4.54 | 131 | 82 | 5.03 | 61.2 | 30.2 | 7.8 |
| N308 | 1 | 39 | 2.76 ± 0.052 | 0 | 0 | 1 | 4.64 | 1.08 | 1.14 | 2.7 | 4.02 | 121 | 82 | 3.84 | 54.2 | 38.8 | 5.7 |
| N309 | 1 | 55 | 5.87 ± 0.145 | 0 | 1 | 1 | 3.56 | 0.88 | 1.21 | 2.12 | 4.8 | 110 | 73 | 4.64 | 56.3 | 31.5 | 8.8 |
| N312 | 2 | 39 | 4.41 ± 0.039 | 1 | 0 | 0 | 4.26 | 1.18 | 1 | 2.56 | 4.23 | 120 | 80 | 6 | 54.5 | 35.5 | 8.5 |
| N313 | 1 | 35 | 5.07 ± 0.116 | 0 | 0 | 0 | 4.27 | 0.75 | 1.19 | 2.56 | 4.66 | 113 | 65 | 6.004 | 53.0 | 36.5 | 6.0 |
| N314 | 2 | 46 | 4.44 ± 0.047 | 0 | 0 | 0 | 3.88 | 0.67 | 1.22 | 2.34 | 4.21 | 101 | 66 | 6.09 | 45.6 | 47.0 | 4.4 |
| N315 | 1 | 57 | 4.68 ± 0.118 | 1 | 0 | 1 | 3.84 | 1.52 | 1.02 | 2.28 | 4.06 | 115 | 77 | 4.93 | 47.5 | 42.0 | 8.3 |
| N316 | 2 | 50 | 5.49 ± 0.135 | 0 | 0 | 1 | 4.14 | 1.6 | 0.95 | 2.53 | 4.32 | 127 | 84 | 4.15 | 69.4 | 24.1 | 3.1 |
| N319 | 1 | 58 | 2.97 ± 0.049 | 1 | 0 | 1 | 4.59 | 0.67 | 1.35 | 2.58 | 4.02 | 106 | 59 | 5 | 54.0 | 30.2 | 11.8 |
| N320 | 1 | 38 | 4.67 ± 0.081 | 1 | 0 | 1 | 5.14 | 0.98 | 1.32 | 2.99 | 4.42 | 106 | 58 | 6.15 | 53.2 | 39.7 | 2.9 |
| N321 | 2 | 58 | 4.78 ± 0.075 | 0 | 0 | 0 | 4.75 | 0.57 | 1.32 | 2.77 | 4.28 | 108 | 67 | 3.73 | 49.6 | 38.9 | 10.7 |
| N322 | 2 | 52 | 4.53 ± 0.089 | 0 | 0 | 0 | 4.21 | 0.81 | 1.1 | 2.63 | 4.1 | 123 | 75 | 5.03 | 54.0 | 38.2 | 6.6 |
| N323 | 1 | 57 | 3.84 ± 0.030 | 1 | 1 | 0 | 4.47 | 0.76 | 1.13 | 2.88 | 3.97 | 91 | 66 | 7.18 | 65.9 | 28.8 | 0.3 |
| N325 | 2 | 45 | 6.31 ± 0.287 | 0 | 0 | 0 | 4.53 | 0.73 | 1.41 | 2.64 | 4.5 | 126 | 74 | 4.62 | 53.7 | 34.2 | 3.2 |
| N326 | 2 | 67 | 4.52 ± 0.086 | 0 | 0 | 1 | 4.28 | 0.67 | 1.57 | 2.68 | 4.02 | 131 | 73 | 4.72 | 51.7 | 30.7 | 9.3 |
| N329 | 1 | 70 | 4.08 ± 0.110 | 0 | 0 | 0 | 4.85 | 1.29 | 1.31 | 2.72 | 4.32 | 111 | 70 | 6.26 | 63.3 | 27.3 | 7.3 |
| N330 | 1 | 45 | 5.45 ± 0.242 | 0 | 0 | 0 | 4.04 | 0.78 | 1.31 | 2.46 | 4.14 | 116 | 67 | 5.01 | 67.7 | 25.9 | 5.8 |
| N332 | 1 | 63 | 5.51 ± 0.147 | 1 | 0 | 0 | 5.14 | 1.38 | 1.33 | 2.95 | 4.68 | 152 | 81 | 4.12 | 48.2 | 41.3 | 8.3 |
| N335 | 2 | 65 | 5.34 ± 0.019 | 1 | 0 | 1 | 4.62 | 0.77 | 1.44 | 2.78 | 4.21 | 100 | 70 | 4.47 | 59.5 | 32.4 | 6.3 |
| N340 | 1 | 63 | 4.43 ± 0.002 | 0 | 1 | 1 | 4.44 | 1.25 | 0.97 | 2.93 | 4.68 | 139 | 66 | 6.12 | 62.0 | 29.1 | 7.8 |
| N341 | 2 | 56 | 4.38 ± 0.480 | 0 | 0 | 0 | 5.17 | 1.24 | 1.64 | 2.9 | 4.21 | 133 | 81 | 4.99 | 52.7 | 40.5 | 4.4 |
| N343 | 2 | 64 | 5.38 ± 0.031 | 0 | 0 | 0 | 4.15 | 1.63 | 1.3 | 2.5 | 4.27 | 140 | 73 | 6.23 | 58.5 | 28.8 | 7.8 |
| N344 | 2 | 58 | 5.19 ± 0.149 | 0 | 0 | 0 | 4.9 | 1.08 | 1.33 | 2.81 | 3.96 | 110 | 70 | 4.84 | 48.6 | 36.4 | 7.4 |
| N345 | 1 | 46 | 5.30 ± 0.083 | 0 | 0 | 0 | 4.54 | 1.03 | 1.09 | 2.94 | 4.79 | 117 | 80 | 6.89 | 57.6 | 32.5 | 6.1 |
| N347 | 1 | 50 | 5.56 ± 0.034 | 1 | 0 | 0 | 4.24 | 0.57 | 1.26 | 2.69 | 4.46 | 129 | 86 | 4.16 | 55.8 | 32.2 | 8.9 |
| N348 | 1 | 57 | 5.81 ± 0.052 | 0 | 0 | 0 | 4.31 | 0.86 | 1.72 | 2.16 | 4.28 | 142 | 87 | 4.65 | 60.1 | 26.5 | 12.5 |
| N349 | 1 | 57 | 4.36 ± 0.094 | 1 | 0 | 1 | 4.06 | 0.79 | 1.35 | 2.45 | 4.05 | 99 | 70 | 5.97 | 45.8 | 45.7 | 5.5 |
| N352 | 1 | 49 | 5.68 ± 0.103 | 0 | 0 | 0 | 4.37 | 1.67 | 1.04 | 2.71 | 4.62 | 109 | 66 | 6.19 | 52.5 | 43.1 | 3.1 |
| N353 | 1 | 52 | 5.06 ± 0.045 | 1 | 0 | 0 | 3.15 | 1.47 | 0.96 | 2.09 | 4.52 | 122 | 81 | 4.85 | 61.4 | 26.6 | 9.3 |
| N354 | 2 | 52 | 5.04 ± 0.059 | 1 | 0 | 1 | 4.84 | 1.27 | 1.33 | 2.91 | 4.03 | 125 | 76 | 5.98 | 60.9 | 32.4 | 5.9 |
| N355 | 1 | 45 | 5.08 ± 0.337 | 0 | 0 | 0 | 4.23 | 1.06 | 1.35 | 2.54 | 4.37 | 97 | 66 | 5.64 | 44.9 | 37.9 | 10.5 |
| N356 | 1 | 54 | 3.88 ± 0.237 | 1 | 0 | 0 | 4.37 | 1.24 | 1.44 | 2.6 | 4.1 | 89 | 68 | 4.39 | 49.0 | 40.8 | 5.9 |
| N357 | 1 | 46 | 5.37 ± 0.333 | 0 | 0 | 0 | 3.77 | 1.45 | 0.94 | 2.47 | 4.98 | 104 | 70 | 7.86 | 62.8 | 28.1 | 7.1 |
| N359 | 1 | 55 | 5.23 ± 0.073 | 0 | 0 | 0 | 4.71 | 0.82 | 1.22 | 2.93 | 4.05 | 102 | 70 | 5.7 | 62.6 | 26.3 | 7.9 |
| N360 | 2 | 58 | 4.39 ± 0.018 | 1 | 0 | 1 | 5.1 | 1.17 | 1.48 | 2.94 | 4.67 | 121 | 73 | 3.5 | 43.4 | 52.0 | 4.0 |
| N361 | 1 | 58 | 4.52 ± 0.001 | 0 | 0 | 0 | 4.17 | 0.87 | 1.01 | 2.75 | 4.99 | 135 | 92 | 4.6 | 50.9 | 38.0 | 8.9 |
| N363 | 2 | 46 | 5.02 ± 0.118 | 0 | 0 | 0 | 4.18 | 1.14 | 1.49 | 2.44 | 4.71 | 156 | 74 | 5.95 | 42.3 | 55.5 | 0.2 |
| N364 | 1 | 65 | 4.89 ± 0.098 | 0 | 0 | 0 | 3.66 | 1.36 | 1.06 | 2.24 | 4.78 | 131 | 81 | 6.86 | 60.2 | 33.1 | 2.9 |
| N367 | 2 | 47 | 3.57 ± 0.019 | 1 | 1 | 0 | 4.7 | 0.56 | 1.39 | 2.83 | 3.98 | 110 | 80 | 4.83 | 57.2 | 33.5 | 7.9 |
| N368 | 1 | 58 | 4.81 ± 0.107 | 0 | 0 | 0 | 4.56 | 1.23 | 0.99 | 2.98 | 4.63 | 73 | 54 | 5.75 | 57.2 | 32.2 | 6.4 |
| N369 | 2 | 52 | 4.97 ± 0.146 | 0 | 0 | 0 | 4.66 | 1.18 | 1.22 | 2.83 | 4.33 | 98 | 61 | 6.42 | 48.6 | 43.8 | 6.7 |
| N376 | 1 | 54 | 3.67 ± 0.132 | 0 | 0 | 0 | 4.86 | 1.23 | 1.35 | 2.45 | 5.08 | 130 | 86 | 6.73 | 65.1 | 30.0 | 2.7 |
| N377 | 2 | 52 | 4.89 ± 0.097 | 0 | 1 | 1 | 3.4 | 0.89 | 1.3 | 2.09 | 4.02 | 100 | 60 | 4.45 | 53.7 | 37.1 | 7.0 |
| N378 | 1 | 53 | 4.64 ± 0.428 | 0 | 0 | 0 | 3.38 | 1.16 | 1.13 | 2.08 | 4.35 | 121 | 84 | 6.01 | 57.1 | 32.1 | 8.0 |
| N380 | 2 | 55 | 5.40 ± 0.427 | 0 | 1 | 1 | 0.71 | 1.42 | 1.16 | 2.89 | 4.02 | 108 | 63 | 6.89 | 65.9 | 25.4 | 7.1 |
| N381 | 2 | 50 | 5.46 ± 0.000 | 1 | 0 | 1 | 3.44 | 0.89 | 1.3 | 1.99 | 4.05 | 106 | 68 | 6.85 | 62.6 | 28.8 | 6.3 |
| N382 | 2 | 49 | 4.89 ± 0.172 | 1 | 0 | 0 | 4.71 | 1 | 1.03 | 3.02 | 4.63 | 101 | 62 | 4.98 | 60.7 | 28.9 | 7.2 |
| N383 | 1 | 52 | 5.02 ± 0.026 | 0 | 0 | 0 | 4.51 | 1.16 | 1.06 | 2.94 | 4.45 | 114 | 77 | 3.81 | 49.9 | 41.7 | 7.9 |
| N384 | 1 | 54 | 4.93 ± 0.290 | 0 | 0 | 0 | 4.44 | 1.44 | 1.21 | 2.58 | 4.9 | 115 | 84 | 4.48 | 67.1 | 21.7 | 8.3 |
| N385 | 1 | 47 | 5.04 ± 0.138 | 0 | 0 | 0 | 4.25 | 0.96 | 1.21 | 2.61 | 4.5 | 117 | 75 | 4.96 | 49.1 | 43.1 | 4.4 |
| N387 | 1 | 51 | 4.47 ± 0.050 | 1 | 0 | 1 | 4.73 | 0.95 | 1.51 | 2.61 | 4.12 | 128 | 80 | 5.03 | 45.5 | 45.5 | 3.4 |
| N388 | 2 | 49 | 5.35 ± 0.779 | 0 | 0 | 0 | 3.22 | 1.02 | 1.24 | 1.95 | 4.55 | 150 | 84 | 9.85 | 82.1 | 13.0 | 4.1 |
| N391 | 1 | 45 | 4.96 ± 0.284 | 0 | 0 | 0 | 3.89 | 0.98 | 0.98 | 2.5 | 4.23 | 105 | 60 | 4.81 | 63.8 | 31.2 | 4.4 |
| N392 | 2 | 49 | 5.86 ± 0.361 | 0 | 0 | 0 | 4.12 | 1.05 | 1.37 | 2.44 | 4.3 | 102 | 56 | 6.03 | 61.2 | 30.7 | 7.6 |
| N393 | 2 | 54 | 4.89 ± 0.658 | 0 | 1 | 1 | 4.52 | 0.84 | 1.15 | 2.93 | 4.78 | 109 | 78 | 4.89 | 49.1 | 40.3 | 3.9 |
| N394 | 1 | 72 | 5.97 ± 0.135 | 0 | 0 | 0 | 4.25 | 0.73 | 1.16 | 2.72 | 4.37 | 126 | 68 | 4.56 | 44.1 | 44.1 | 9.4 |
| N399 | 1 | 56 | 6.19 ± 0.262 | 0 | 0 | 0 | 4.57 | 0.99 | 1.17 | 3.01 | 5.13 | 120 | 70 | 5.68 | 37.6 | 51.6 | 6.2 |
| N400 | 2 | 55 | 5.07 ± 0.115 | 0 | 0 | 0 | 4.1 | 0.77 | 1.35 | 2.46 | 4.11 | 116 | 65 | 5.01 | 68.9 | 28.9 | 1.4 |
| N403 | 2 | 60 | 3.13 ± 0.014 | 1 | 0 | 0 | 4.63 | 1.12 | 1.42 | 2.65 | 4.56 | 127 | 89 | 4.71 | 60.5 | 34.2 | 4.2 |
| N404 | 1 | 53 | 3.70 ± 0.224 | 1 | 0 | 1 | 4.27 | 0.66 | 1.95 | 2.03 | 4.68 | 140 | 104 | 7.19 | 44.3 | 33.5 | 7.6 |
| N405 | 2 | 50 | 5.05 ± 0.021 | 1 | 0 | 0 | 4.94 | 1.14 | 1.26 | 3.1 | 4.25 | 110 | 72 | 5.1 | 51.3 | 42.4 | 4.7 |
| N406 | 2 | 51 | 5.60 ± 0.175 | 1 | 0 | 1 | 4.16 | 1.03 | 1.21 | 2.65 | 4.24 | 126 | 69 | 7.41 | 65.3 | 29.8 | 2.6 |
| N408 | 1 | 47 | 4.15 ± 0.224 | 0 | 0 | 0 | 4.65 | 0.55 | 1.22 | 2.9 | 4.17 | 118 | 70 | 4.32 | 44.9 | 44.7 | 8.8 |
| N414 | 1 | 59 | 4.63 ± 0.045 | 0 | 0 | 0 | 4.59 | 0.96 | 0.99 | 2.89 | 4.38 | 110 | 70 | 6.59 | 56.7 | 36.0 | 5.9 |
| N416 | 2 | 45 | 4.05 ± 0.550 | 1 | 0 | 0 | 4.33 | 0.79 | 1.03 | 2.84 | 4.05 | 101 | 59 | 6.8 | 64.3 | 27.9 | 6.6 |
| N417 | 1 | 46 | 3.61 ± 0.012 | 1 | 1 | 0 | 4.43 | 0.89 | 1.56 | 2.58 | 4.24 | 90 | 60 | 5.65 | 44.8 | 47.3 | 1.2 |
| N418 | 1 | 53 | 3.50 ± 0.033 | 0 | 1 | 1 | 4.76 | 0.68 | 1.22 | 3.05 | 4.21 | 120 | 80 | 5.73 | 62.3 | 33.7 | 2.3 |
| N425 | 2 | 53 | 5.33 ± 0.372 | 0 | 0 | 0 | 4.93 | 0.57 | 1.68 | 2.68 | 4.28 | 101 | 64 | 4.98 | 61.5 | 33.9 | 2.0 |
| N426 | 1 | 59 | 4.64 ± 0.093 | 0 | 0 | 0 | 3.93 | 0.66 | 1.29 | 2.4 | 4.09 | 123 | 76 | 5.52 | 52.2 | 40.2 | 3.1 |
| N427 | 2 | 60 | 3.40 ± 0.068 | 0 | 1 | 1 | 4.25 | 1.5 | 0.92 | 2.82 | 4.91 | 113 | 62 | 5.24 | 60.3 | 35.9 | 2.1 |
| N428 | 2 | 61 | 3.01 ± 0.049 | 1 | 0 | 0 | 3.82 | 1.13 | 1.12 | 2.38 | 4.8 | 129 | 87 | 4.77 | 44.4 | 46.8 | 7.8 |
| N429 | 2 | 60 | 5.07 ± 0.048 | 0 | 0 | 0 | 4.06 | 0.9 | 1.24 | 2.48 | 4.51 | 120 | 64 | 4.12 | 57.3 | 34.7 | 5.8 |
| N430 | 1 | 62 | 3.56 ± 0.000 | 1 | 0 | 0 | 4.16 | 1.02 | 0.97 | 2.8 | 4.64 | 112 | 62 | 4.86 | 47.1 | 42.2 | 9.5 |
| N432 | 1 | 51 | 6.00 ± 0.027 | 0 | 0 | 0 | 4.04 | 0.67 | 1.67 | 2.1 | 4.12 | 94 | 67 | 5.28 | 56.5 | 29.9 | 12.3 |
| N433 | 1 | 59 | 5.44 ± 0.276 | 1 | 0 | 0 | 3.84 | 0.7 | 1.01 | 2.62 | 5.3 | 142 | 79 | 5.4 | 60.9 | 36.1 | 0.0 |
| N434 | 1 | 51 | 3.39 ± 0.068 | 1 | 0 | 0 | 4.5 | 1.03 | 1.21 | 2.7 | 4.57 | 110 | 66 | 4.04 | 57.9 | 36.1 | 3.0 |
| N436 | 2 | 49 | 5.20 ± 0.155 | 0 | 1 | 1 | 3.98 | 0.71 | 1.18 | 2.3 | 4.25 | 122 | 91 | 5 | 63.2 | 27.8 | 6.6 |
| N437 | 1 | 56 | 3.93 ± 0.098 | 1 | 0 | 0 | 4.67 | 0.68 | 1.14 | 2.72 | 5.19 | 109 | 71 | 4.93 | 59.7 | 30.4 | 7.9 |
| N438 | 1 | 64 | 3.84 ± 0.075 | 0 | 0 | 0 | 4.96 | 1.42 | 0.96 | 2.9 | 5.26 | 148 | 88 | 4.81 | 46.9 | 46.8 | 4.2 |
| N439 | 1 | 59 | 4.00 ± 0.100 | 1 | 0 | 0 | 4.79 | 1.06 | 1.19 | 2.78 | 4.64 | 126 | 71 | 7.32 | 56.0 | 34.3 | 7.1 |
| N440 | 1 | 61 | 5.49 ± 0.146 | 0 | 0 | 0 | 3.29 | 0.64 | 1.1 | 1.96 | 5.53 | 139 | 101 | 5.32 | 69.2 | 22.9 | 6.8 |
| N441 | 2 | 45 | 5.38 ± 0.115 | 1 | 0 | 1 | 4.11 | 0.82 | 1.37 | 2.26 | 5.61 | 100 | 64 | 5.69 | 56.3 | 33.7 | 4.9 |
| N442 | 1 | 56 | 3.78 ± 0.005 | 1 | 0 | 0 | 4.6 | 1.44 | 0.97 | 2.65 | 5.59 | 128 | 83 | 4.64 | 47.1 | 43.8 | 6.5 |
| N443 | 2 | 45 | 3.57 ± 0.012 | 1 | 0 | 0 | 4.01 | 0.95 | 1.02 | 2.35 | 5.46 | 102 | 61 | 4.07 | 50.9 | 36.1 | 11.3 |
| N444 | 1 | 58 | 3.03 ± 0.033 | 0 | 1 | 1 | 4.37 | 0.69 | 1.19 | 2.44 | 5.48 | 116 | 74 | 6.56 | 60.6 | 30.2 | 7.8 |
| N445 | 2 | 43 | 4.43 ± 0.213 | 1 | 0 | 0 | 4.48 | 0.88 | 1.67 | 2.28 | 5.54 | 128 | 83 | 3.62 | 49.4 | 44.2 | 5.8 |
| N446 | 2 | 58 | 3.80 ± 0.319 | 1 | 0 | 1 | 4.7 | 0.9 | 1.25 | 2.76 | 5.58 | 125 | 80 | 7.42 | 55.1 | 37.5 | 5.9 |
| N447 | 1 | 51 | 3.68 ± 0.156 | 0 | 0 | 0 | 4.27 | 0.83 | 1.14 | 2.51 | 4.89 | 96 | 54 | 3.82 | 44.5 | 47.6 | 5.0 |
| N448 | 1 | 58 | 5.72 ± 0.071 | 0 | 0 | 0 | 3.92 | 0.59 | 1.27 | 2.3 | 5.51 | 102 | 65 | 3.86 | 47.4 | 43.0 | 8.8 |
| N449 | 2 | 39 | 5.77 ± 0.057 | 0 | 1 | 1 | 5.15 | 1.47 | 1.67 | 2.54 | 5.24 | 100 | 64 | 4.37 | 49.9 | 37.3 | 5.7 |
| N451 | 1 | 48 | 5.24 ± 0.003 | 1 | 0 | 0 | 4.74 | 0.75 | 1.29 | 2.7 | 4.76 | 106 | 76 | 6.14 | 59.1 | 32.4 | 7.5 |
| N457 | 1 | 48 | 6.61 ± 0.118 | 0 | 0 | 0 | 4.69 | 1.37 | 1.39 | 2.64 | 4.35 | 120 | 80 | 6.05 | 61.4 | 27.8 | 9.2 |
| N458 | 2 | 64 | 4.14 ± 0.007 | 1 | 0 | 1 | 4.42 | 1.31 | 1.41 | 2.48 | 5.13 | 97 | 59 | 5.7 | 55.5 | 32.8 | 9.6 |
| N459 | 2 | 49 | 4.81 ± 0.085 | 1 | 0 | 0 | 3.88 | 0.77 | 1.15 | 2.34 | 5.03 | 117 | 69 | 6.04 | 52.8 | 36.4 | 7.9 |
| N464 | 1 | 81 | 4.59 ± 0.045 | 0 | 1 | 1 | 4.92 | 1.25 | 1.26 | 2.9 | 5.02 | 113 | 61 | 4.53 | 52.9 | 32.0 | 9.8 |
| N465 | 2 | 57 | 4.52 ± 0.003 | 0 | 0 | 0 | 4.8 | 1.67 | 1.14 | 2.75 | 4.97 | 142 | 81 | 3.47 | 65.5 | 29.1 | 4.3 |
| N466 | 2 | 59 | 3.77 ± 0.153 | 0 | 0 | 1 | 4.85 | 0.84 | 1.55 | 2.72 | 4.53 | 147 | 88 | 7.37 | 56.9 | 30.3 | 6.3 |
| N467 | 1 | 56 | 4.02 ± 0.077 | 1 | 0 | 0 | 4.51 | 1.65 | 1.11 | 2.63 | 4.4 | 160 | 58 | 6.56 | 57.5 | 27.1 | 8.3 |
| N469 | 2 | 48 | 3.84 ± 0.014 | 0 | 1 | 1 | 4.59 | 0.97 | 1.54 | 2.47 | 5.24 | 125 | 72 | 5.1 | 48.6 | 40.2 | 8.2 |
| N472 | 2 | 59 | 4.10 ± 0.118 | 1 | 0 | 0 | 4.33 | 0.75 | 1.17 | 2.65 | 5.45 | 128 | 76 | 5.25 | 54.0 | 33.2 | 9.1 |
| N475 | 1 | 58 | 3.37 ± 0.102 | 0 | 0 | 0 | 5.1 | 0.84 | 1.29 | 3 | 5.36 | 121 | 71 | 5.73 | 58.6 | 28.0 | 10.9 |
| N482 | 1 | 58 | 4.02 ± 0.039 | 0 | 1 | 1 | 3.97 | 1.24 | 1.23 | 1.94 | 5.26 | 111 | 71 | 4.71 | 55.2 | 34.9 | 7.0 |
| N485 | 2 | 47 | 5.16 ± 0.025 | 1 | 0 | 0 | 4.59 | 1.04 | 1.26 | 2.74 | 4.63 | 102 | 60 | 5.47 | 50.1 | 40.1 | 8.5 |
| N486 | 1 | 53 | 3.69 ± 0.015 | 1 | 0 | 0 | 4.41 | 0.83 | 1.26 | 2.59 | 5.33 | 121 | 67 | 5.92 | 56.6 | 34.8 | 7.3 |
| N488 | 1 | 48 | 3.52 ± 0.143 | 1 | 0 | 1 | 4.78 | 0.88 | 1.1 | 2.91 | 4.59 | 137 | 75 | 6.36 | 62.3 | 26.8 | 8.5 |
| N489 | 2 | 41 | 3.75 ± 0.030 | 0 | 0 | 0 | 3.2 | 0.99 | 1.39 | 1.6 | 5.12 | 125 | 69 | 5.3 | 53.1 | 34.6 | 6.5 |
| N490 | 1 | 57 | 3.79 ± 0.024 | 0 | 0 | 0 | 4.22 | 0.74 | 1.25 | 2.49 | 4.71 | 98 | 61 | 5.97 | 45.6 | 47.3 | 4.7 |
| N495 | 2 | 55 | 4.05 ± 0.215 | 1 | 0 | 1 | 4.91 | 1.17 | 1.44 | 2.67 | 4.6 | 110 | 60 | 3.89 | 49.7 | 39.6 | 7.6 |
| N503 | 2 | 73 | 3.66 ± 0.036 | 0 | 0 | 0 | 4.43 | 0.98 | 1.21 | 2.69 | 4.73 | 112 | 76 | 4.38 | 51.7 | 34.8 | 8.7 |
| N507 | 1 | 51 | 3.70 ± 0.126 | 0 | 0 | 0 | 3.82 | 1.55 | 0.96 | 2.48 | 4.71 | 103 | 68 | 6.14 | 56.0 | 31.8 | 10.0 |
| N510 | 1 | 67 | 4.57 ± 0.167 | 1 | 0 | 0 | 3.81 | 1.26 | 1.1 | 2.28 | 4.79 | 150 | 80 | 11.6 | 67.2 | 23.6 | 7.6 |
| N512 | 2 | 51 | 3.77 ± 0.162 | 0 | 0 | 0 | 4.25 | 0.78 | 1.62 | 2.22 | 3.98 | 106 | 67 | 6.69 | 84.9 | 8.1 | 5.8 |
| N514 | 2 | 59 | 3.63 ± 0.109 | 0 | 0 | 0 | 5.17 | 1.13 | 1.34 | 3 | 4.25 | 149 | 78 | 5 | 50.8 | 42.4 | 5.6 |
| N518 | 1 | 56 | 3.95 ± 0.020 | 0 | 0 | 0 | 5.17 | 1.71 | 1.44 | 2.86 | 4 | 121 | 85 | 4.64 | 68.7 | 21.0 | 7.9 |
| N523 | 1 | 80 | 3.34 ± 0.030 | 0 | 0 | 0 | 4.94 | 1.01 | 1.33 | 2.83 | 4.02 | 116 | 72 | 6.38 | 65.3 | 21.8 | 10.9 |
| N524 | 2 | 76 | 3.67 ± 0.081 | 0 | 0 | 0 | 5.04 | 1.24 | 1.42 | 2.75 | 4.7 | 86 | 44 | 5.48 | 61.9 | 30.3 | 6.7 |
| N527 | 1 | 71 | 3.92 ± 0.050 | 1 | 0 | 0 | 4.51 | 0.65 | 1.91 | 2.08 | 5.16 | 125 | 75 | 3.89 | 48.2 | 39.0 | 10.9 |
| N530 | 2 | 48 | 3.93 ± 0.010 | 0 | 1 | 1 | 4.74 | 0.59 | 1.37 | 2.71 | 4.05 | 108 | 74 | 5.29 | 43.8 | 45.1 | 7.1 |
| N534 | 2 | 69 | 3.78 ± 0.005 | 0 | 0 | 0 | 4.96 | 1.2 | 1.36 | 2.98 | 4.29 | 98 | 59 | 3.82 | 55.6 | 35.2 | 7.8 |
| N535 | 2 | 52 | 3.79 ± 0.108 | 0 | 0 | 0 | 5.14 | 1.06 | 1.48 | 3.02 | 4.6 | 100 | 60 | 4.74 | 57.3 | 34.0 | 6.3 |
| N563 | 1 | 60 | 4.13 ± 0.002 | 1 | 0 | 1 | 4.04 | 0.97 | 0.99 | 2.65 | 4.99 | 120 | 87 | 6.71 | 62.4 | 26.9 | 8.0 |
| N565 | 1 | 73 | 3.56 ± 0.302 | 0 | 0 | 0 | 3.63 | 0.63 | 1.1 | 2.25 | 4.4 | 98 | 61 | 4.8 | 52.4 | 34.4 | 8.7 |
| N567 | 1 | 50 | 4.20 ± 0.070 | 0 | 1 | 0 | 5.03 | 0.78 | 1.43 | 2.98 | 4.35 | 136 | 80 | 4.99 | 51.9 | 32.2 | 8.2 |
| N571 | 1 | 48 | 3.77 ± 0.060 | 0 | 0 | 0 | 4.67 | 0.63 | 1.33 | 2.73 | 4.04 | 109 | 64 | 6.78 | 51.9 | 35.0 | 10.9 |
| N572 | 1 | 73 | 3.50 ± 0.044 | 0 | 0 | 0 | 4.45 | 0.86 | 1.16 | 2.88 | 5.74 | 136 | 73 | 6.01 | 44.4 | 39.5 | 8.2 |
| N573 | 2 | 41 | 3.29 ± 0.116 | 0 | 1 | 0 | 4.64 | 0.62 | 1.63 | 2.51 | 4.24 | 107 | 73 | 4.53 | 57.3 | 30.7 | 9.3 |
| N580 | 1 | 55 | 3.45 ± 0.049 | 1 | 1 | 1 | 4.37 | 1.4 | 1.04 | 2.59 | 4.15 | 121 | 82 | 6.84 | 59.9 | 27.1 | 9.7 |
| N584 | 2 | 69 | 3.66 ± 0.324 | 0 | 1 | 0 | 4.07 | 1.6 | 1.19 | 2.29 | 4.6 | 106 | 50 | 4.04 | 50.2 | 32.6 | 10.0 |
| N601 | 2 | 64 | 3.22 ± 0.093 | 0 | 0 | 1 | 3.62 | 0.76 | 1.28 | 2.05 | 4.84 | 106 | 45 | 2.99 | 47.0 | 39.1 | 9.5 |
| N602 | 2 | 62 | 3.13 ± 0.015 | 0 | 0 | 0 | 4.62 | 1.58 | 1.2 | 2.64 | 5.52 | 134 | 66 | 4.46 | 51.2 | 39.9 | 7.3 |
| N603 | 1 | 69 | 3.23 ± 0.037 | 0 | 0 | 0 | 4.56 | 1.16 | 1.7 | 1.93 | 4.47 | 115 | 73 | 4.7 | 43.4 | 48.2 | 6.2 |
| N607 | 2 | 51 | 3.33 ± 0.075 | 1 | 0 | 0 | 5.05 | 1.37 | 1.02 | 3.07 | 4.86 | 110 | 67 | 5.06 | 36.4 | 56.0 | 6.1 |
| N610 | 1 | 70 | 4.18 ± 0.421 | 0 | 1 | 0 | 4.23 | 1.05 | 1.7 | 2.1 | 5.98 | 117 | 67 | 4.19 | 61.2 | 25.2 | 7.8 |
| N615 | 1 | 74 | 4.02 ± 0.105 | 0 | 1 | 0 | 3.75 | 0.61 | 1.05 | 2.33 | 5.16 | 142 | 71 | 4.79 | 59.5 | 28.0 | 10.1 |
| N620 | 2 | 61 | 3.87 ± 0.246 | 0 | 0 | 0 | 4.18 | 0.61 | 1.33 | 2.33 | 4.68 | 116 | 70 | 3.95 | 60.7 | 26.4 | 11.0 |
| N622 | 1 | 64 | 3.91 ± 0.006 | 1 | 0 | 0 | 4.37 | 0.57 | 1.38 | 2.45 | 4.84 | 100 | 65 | 6.12 | 51.3 | 34.7 | 10.1 |
| N625 | 2 | 60 | 3.89 ± 0.075 | 0 | 0 | 0 | 5.06 | 1.01 | 1.32 | 2.89 | 5.24 | 156 | 68 | 5.58 | 54.7 | 37.4 | 5.8 |
| N626 | 1 | 71 | 3.49 ± 0.022 | 0 | 0 | 0 | 3.56 | 1.27 | 0.96 | 2.14 | 5.45 | 120 | 71 | 5.61 | 58.3 | 31.9 | 7.0 |
| N629 | 1 | 64 | 4.14 ± 0.233 | 0 | 0 | 0 | 4.26 | 0.89 | 1.11 | 2.79 | 4.36 | 76 | 52 | 6.6 | 60.4 | 30.2 | 7.7 |
| N630 | 1 | 71 | 4.13 ± 0.218 | 0 | 1 | 0 | 4.62 | 1.16 | 1.07 | 2.91 | 5.61 | 152 | 73 | 3.62 | 61.2 | 28.1 | 8.2 |
| N632 | 1 | 74 | 4.34 ± 0.019 | 1 | 0 | 0 | 3.83 | 1.03 | 0.96 | 2.38 | 5.07 | 108 | 60 | 5.68 | 63.6 | 26.2 | 8.5 |
| N635 | 2 | 56 | 3.73 ± 0.176 | 0 | 1 | 0 | 4.75 | 1.3 | 1.23 | 2.79 | 4.86 | 115 | 69 | 5.02 | 56.9 | 34.5 | 6.1 |
| N636 | 1 | 55 | 4.60 ± 0.137 | 1 | 1 | 0 | 4.03 | 0.91 | 1.08 | 2.49 | 4.32 | 87 | 64 | 5.62 | 52.8 | 33.1 | 7.8 |
| N640 | 1 | 76 | 4.32 ± 0.037 | 0 | 1 | 0 | 5.08 | 0.75 | 1.29 | 3.07 | 4.68 | 116 | 53 | 3.89 | 49.4 | 37.4 | 9.7 |
| N641 | 2 | 47 | 4.43 ± 0.068 | 0 | 0 | 0 | 4.35 | 1.11 | 1.2 | 2.61 | 4.64 | 112 | 68 | 4.82 | 57.7 | 32.7 | 7.3 |
| N645 | 2 | 57 | 4.54 ± 0.107 | 1 | 0 | 0 | 5.18 | 0.76 | 1.33 | 3.04 | 4.73 | 110 | 65 | 4.48 | 64.7 | 26.0 | 7.1 |
| N650 | 1 | 73 | 3.59 ± 0.123 | 0 | 0 | 0 | 4.37 | 0.99 | 1.48 | 2.41 | 5.15 | 132 | 53 | 4.61 | 54.5 | 34.6 | 9.2 |
| N652 | 1 | 75 | 5.52 ± 0.217 | 0 | 1 | 0 | 4.5 | 0.64 | 1.11 | 2.83 | 4.82 | 132 | 73 | 5.31 | 58.8 | 19.9 | 9.9 |
| N655 | 1 | 71 | 4.73 ± 0.051 | 0 | 0 | 0 | 4.02 | 0.93 | 1.13 | 2.39 | 4.89 | 146 | 76 | 5.43 | 47.1 | 41.7 | 8.5 |
| N656 | 2 | 47 | 4.60 ± 0.242 | 0 | 0 | 0 | 3.8 | 1.05 | 1.15 | 2.46 | 5.32 | 121 | 65 | 6.36 | 59.0 | 31.9 | 5.8 |
| N658 | 1 | 53 | 4.85 ± 0.269 | 0 | 1 | 0 | 4.84 | 1.54 | 0.94 | 2.78 | 4.67 | 126 | 80 | 7.1 | 67.0 | 22.8 | 7.9 |
| N661 | 1 | 46 | 6.56 ± 0.343 | 0 | 0 | 0 | 4.65 | 0.62 | 1.07 | 3.03 | 4.68 | 132 | 66 | 5.18 | 45.3 | 36.6 | 9.1 |
| N663 | 2 | 61 | 5.91 ± 0.078 | 1 | 0 | 0 | 4.53 | 0.6 | 1.79 | 2.46 | 4.75 | 132 | 79 | 2.97 | 50.6 | 37.8 | 8.4 |
| N704 | 1 | 62 | 4.08 ± 0.129 | 0 | 1 | 0 | 4.58 | 1.37 | 1.07 | 2.84 | 4.16 | 122 | 77 | 6.8 | 67.7 | 15.7 | 14.2 |
| N705 | 1 | 58 | 6.18 ± 0.265 | 0 | 0 | 1 | 3.99 | 1.24 | 1.08 | 2.4 | 4.62 | 140 | 76 | 6.6 | 57.3 | 31.9 | 5.9 |
| N706 | 2 | 58 | 5.54 ± 0.007 | 1 | 0 | 0 | 4.53 | 1.05 | 1.39 | 2.62 | 4.35 | 100 | 53 | 4.9 | 58.9 | 32.6 | 6.9 |
| N707 | 1 | 57 | 6.11 ± 0.325 | 0 | 1 | 0 | 4.3 | 0.95 | 1.04 | 2.74 | 4.41 | 117 | 72 | 5.3 | 54.0 | 33.9 | 6.9 |
| N718 | 1 | 50 | 5.94 ± 0.124 | 1 | 0 | 0 | 4.09 | 0.56 | 1.69 | 2.24 | 5.46 | 115 | 62 | 3.5 | 48.4 | 40.4 | 5.7 |
| N720 | 2 | 57 | 5.32 ± 0.003 | 0 | 0 | 0 | 4.97 | 1.03 | 1.33 | 2.92 | 4.99 | 139 | 66 | 5.1 | 62.7 | 30.5 | 5.8 |
| N723 | 1 | 69 | 6.06 ± 0.042 | 0 | 1 | 1 | 3.63 | 0.57 | 1.67 | 1.89 | 4.76 | 91 | 57 | 6 | 65.5 | 22.4 | 9.8 |
| N725 | 1 | 70 | 5.95 ± 0.151 | 0 | 0 | 0 | 5.1 | 0.91 | 1.37 | 3.01 | 4.87 | 113 | 84 | 7 | 59.6 | 29.0 | 9.0 |
| N730 | 1 | 73 | 5.02 ± 0.791 | 0 | 1 | 0 | 4.31 | 1.47 | 0.96 | 2.75 | 4.87 | 116 | 80 | 6.4 | 59.4 | 29.2 | 8.9 |
| N734 | 2 | 52 | 5.60 ± 0.086 | 1 | 0 | 0 | 4.52 | 0.56 | 1.37 | 2.65 | 4.54 | 100 | 70 | 4.1 | 54.3 | 37.1 | 6.0 |
| N735 | 2 | 55 | 3.42 ± 0.277 | 0 | 0 | 0 | 4.53 | 0.65 | 1.33 | 2.71 | 5.8 | 119 | 72 | 4.31 | 52.0 | 38.6 | 7.9 |
| N736 | 2 | 64 | 4.11 ± 0.585 | 0 | 1 | 0 | 4.82 | 1.43 | 1.24 | 2.84 | 4.83 | 124 | 66 | 3.97 | 36.7 | 49.6 | 8.9 |
| N738 | 1 | 53 | 4.72 ± 0.005 | 0 | 0 | 0 | 4.9 | 1.09 | 1.26 | 3.02 | 4.94 | 158 | 60 | 5.38 | 63.9 | 27.4 | 5.8 |
| N740 | 2 | 56 | 4.52 ± 0.264 | 0 | 1 | 0 | 4.03 | 0.95 | 1.19 | 2.41 | 4.66 | 111 | 83 | 4.07 | 58.7 | 32.0 | 6.3 |
| N741 | 1 | 54 | 4.26 ± 0.129 | 0 | 0 | 0 | 4.67 | 0.78 | 1.29 | 2.89 | 4.47 | 132 | 86 | 7.63 | 39.4 | 49.6 | 8.8 |
| N742 | 2 | 61 | 4.02 ± 0.157 | 1 | 0 | 0 | 4.25 | 0.91 | 1.3 | 2.57 | 4.57 | 138 | 81 | 3.52 | 48.5 | 41.0 | 8.1 |
| N743 | 1 | 67 | 4.74 ± 0.063 | 1 | 1 | 0 | 3.72 | 0.79 | 0.97 | 2.49 | 5.04 | 109 | 72 | 6.4 | 57.2 | 36.7 | 5.0 |
| N744 | 1 | 58 | 3.95 ± 0.093 | 0 | 0 | 0 | 4.03 | 1.09 | 1.13 | 2.48 | 4.77 | 98 | 55 | 4.94 | 58.5 | 30.0 | 7.4 |
| N745 | 2 | 56 | 5.72 ± 0.132 | 0 | 0 | 0 | 4.61 | 1.31 | 1.15 | 2.91 | 5.06 | 147 | 72 | 3.23 | 45.3 | 44.2 | 7.0 |
| N749 | 2 | 65 | 4.15 ± 0.282 | 0 | 1 | 0 | 4.51 | 0.74 | 1.94 | 2.26 | 4.34 | 118 | 74 | 5.54 | 43.5 | 41.7 | 9.7 |
| N756 | 1 | 51 | 3.94 ± 0.114 | 0 | 1 | 0 | 4.55 | 1.03 | 1.17 | 2.83 | 4.68 | 123 | 82 | 5.02 | 66.2 | 23.1 | 8.4 |
| N758 | 1 | 62 | 5.02 ± 0.580 | 0 | 0 | 0 | 4.47 | 0.82 | 1.44 | 2.64 | 4.42 | 115 | 77 | 5.1 | 59.7 | 33.3 | 5.6 |
| N761 | 2 | 61 | 5.27 ± 0.209 | 1 | 0 | 1 | 5.12 | 1.57 | 1.33 | 2.99 | 4.76 | 115 | 68 | 5.84 | 57.0 | 33.0 | 8.6 |
| N782 | 1 | 62 | 4.53 ± 0.349 | 1 | 1 | 0 | 3.08 | 0.58 | 1.18 | 1.92 | 4.64 | 116 | 77 | 6.23 | 59.8 | 27.2 | 6.8 |
| N784 | 2 | 48 | 5.42 ± 0.136 | 0 | 1 | 0 | 4.44 | 1.23 | 1.6 | 2.47 | 4.23 | 121 | 83 | 6.6 | 70.4 | 20.1 | 7.2 |
| N788 | 1 | 53 | 4.59 ± 0.468 | 0 | 0 | 0 | 3.68 | 0.87 | 1.04 | 2.5 | 4.35 | 120 | 70 | 8.7 | 65.0 | 26.4 | 6.8 |
| N792 | 1 | 56 | 5.40 ± 0.077 | 0 | 1 | 0 | 4.23 | 0.79 | 1.32 | 2.53 | 3.94 | 110 | 89 | 6.6 | 59.8 | 30.2 | 8.5 |
| N805 | 2 | 57 | 4.26 ± 0.219 | 0 | 0 | 0 | 4.81 | 1.07 | 1.3 | 2.89 | 5.33 | 135 | 83 | 4.8 | 63.9 | 28.3 | 6.9 |
| N806 | 2 | 63 | 3.35 ± 0.307 | 1 | 0 | 0 | 4.17 | 1.3 | 1.26 | 2.33 | 4.3 | 121 | 73 | 3.5 | 58.6 | 29.7 | 8.6 |
| N812 | 1 | 70 | 3.38 ± 0.180 | 0 | 1 | 1 | 4.47 | 1.55 | 0.98 | 2.85 | 4.96 | 120 | 69 | 3.9 | 56.8 | 29.4 | 10.1 |
| N814 | 1 | 31 | 3.86 ± 0.515 | 1 | 0 | 0 | 4.15 | 0.83 | 1.48 | 2.37 | 4.73 | 110 | 70 | 6.5 | 49.3 | 38.6 | 8.1 |
| N840 | 1 | 55 | 5.57 ± 0.073 | 1 | 0 | 0 | 5.1 | 0.82 | 1.58 | 2.7 | 4.52 | 122 | 81 | 3.44 | 63.6 | 26.7 | 7.9 |
| N842 | 2 | 56 | 5.55 ± 0.231 | 0 | 0 | 0 | 3.54 | 0.58 | 1.37 | 2.05 | 5.45 | 116 | 64 | 5.6 | 62.4 | 27.6 | 7.8 |
| N844 | 2 | 60 | 3.44 ± 0.285 | 0 | 1 | 1 | 4.57 | 0.96 | 1.32 | 2.63 | 5.23 | 109 | 62 | 5.6 | 65.4 | 22.2 | 7.2 |
| N848 | 2 | 56 | 3.20 ± 0.123 | 0 | 0 | 0 | 4.26 | 0.57 | 1.25 | 2.61 | 5.13 | 136 | 68 | 4.4 | 48.3 | 41.3 | 7.3 |
| N853 | 1 | 77 | 3.75 ± 0.155 | 0 | 0 | 0 | 4.9 | 1.43 | 1.01 | 3.05 | 4.46 | 111 | 72 | 10.8 | 63.3 | 27.8 | 5.7 |
| N854 | 2 | 63 | 4.03 ± 0.037 | 0 | 1 | 1 | 4.14 | 1.25 | 1.16 | 2.52 | 4.93 | 118 | 61 | 4.8 | 60.9 | 32.0 | 6.4 |
| N856 | 1 | 48 | 4.55 ± 0.107 | 0 | 0 | 0 | 5.06 | 1.39 | 1.12 | 3.03 | 4.59 | 117 | 87 | 5.2 | 54.9 | 34.0 | 5.7 |
| N867 | 1 | 61 | 4.25 ± 0.141 | 1 | 1 | 0 | 4.75 | 1.01 | 1.07 | 2.14 | 4.7 | 126 | 88 | 7.8 | 57.6 | 31.8 | 9.5 |
| N868 | 1 | 55 | 3.34 ± 0.005 | 0 | 0 | 0 | 4.19 | 0.62 | 1.48 | 2.35 | 4.93 | 110 | 70 | 3.5 | 58.3 | 31.3 | 8.3 |
| N873 | 1 | 52 | 3.36 ± 0.153 | 0 | 0 | 0 | 4.6 | 0.64 | 1.72 | 2.44 | 4.73 | 91 | 62 | 3.7 | 47.6 | 45.1 | 5.9 |
| N876 | 2 | 48 | 3.85 ± 0.046 | 0 | 1 | 1 | 4.75 | 0.66 | 1.36 | 2.86 | 5.05 | 104 | 64 | 4.5 | 52.3 | 38.3 | 7.5 |
| N877 | 2 | 49 | 3.23 ± 0.133 | 0 | 0 | 1 | 4.28 | 0.6 | 1.37 | 2.57 | 4.34 | 100 | 61 | 4.6 | 48.0 | 43.4 | 6.4 |
| N878 | 1 | 67 | 5.91 ± 0.518 | 1 | 1 | 0 | 3.66 | 0.88 | 1.02 | 2.45 | 4.59 | 116 | 59 | 3.7 | 58.5 | 29.5 | 9.3 |
| ^a^ 1 stands for Male, 2 stands for Female.  ^b^ The status with ( represented by 1) or without (represented by 0) specific medical conditions rather than unstable clinical examination results were recorded in this research.  TC, total cholesterol; TG, total triglyceride; HDL-C, high density lipoprotein cholesterol; LDL-C, low-density lipoprotein cholesterol; FBG, fasting blood glucose; SBP, [systolic](javascript:void(0);) blood [pressure](javascript:void(0);); DBP, diastolic blood pressure; CBC, complete blood count; PBL, peripheral blood leukocyte; NEU, neutrophil; LYM, lymphocyte; MONO, monocyte. | | | | | | | | | | | | | | | | | |

**Table S2.** Clinical characteristics and measure contents of 5-mdC in genomic DNA of blood from 215 CAD patients.

| Sample ID | Gender^a^ | Age | 5-mdC, % | HT^b^ | HL^b^ | DM^b^ | TC | TG | HLL-c | LDL-c | FBG | CBC | | | |
| --- | --- | --- | --- | --- | --- | --- | --- | --- | --- | --- | --- | --- | --- | --- | --- |
|  |  |  |  |  |  |  | (mmol/L) | | | | | PBL(10^9^/L) | NEU% | LYM% | MONO% |
| C275 | 1 | 48 | 4.56 ± 0.267 | 1 | 0 | 0 | 4.87 | 1.1 | 1.52 | 2.84 | 4.63 | 7.36 | 64.9 | 26.2 | 7.1 |
| C276 | 1 | 71 | 4.29 ± 0.044 | 1 | 0 | 0 | 3.46 | 1.33 | 0.96 | 1.82 | 6.12 | 11.37 | 87.0 | 7.4 | 4.8 |
| C279 | 1 | 55 | 4.10 ± 0.102 | 1 | 0 | 0 | 3.01 | 0.97 | 1.05 | 1.74 | 4.87 | 3.4 | 51.2 | 34.7 | 11.8 |
| C281 | 2 | 67 | 3.72 ± 0.176 | 1 | 0 | 0 | 5.71 | 0.91 | 1.72 | 3.43 | 5.53 | 5 | 81.0 | 22.8 | 5.4 |
| C283 | 1 | 48 | 3.81 ± 0.249 | 0 | 0 | 0 | 4.96 | 2.7 | 1.04 | 2.68 | 5.48 | 5.79 | 43.5 | 31.3 | 6.9 |
| C284 | 1 | 54 | 3.58 ± 0.242 | 1 | 0 | 0 | 4.82 | 5.85 | 0.77 | 1.71 | 3.93 | 9.51 | 57.5 | 35.2 | 3.7 |
| C289 | 2 | 42 | 3.28 ± 0.157 | 0 | 1 | 0 | 6.63 | 2.4 | 1.03 | 4.44 | 5.46 | 9.29 | 57.6 | 31.7 | 9.4 |
| C291 | 1 | 59 | 3.29 ± 0.009 | 1 | 0 | 0 | 6.3 | 3.24 | 1.12 | 4.01 | 12.46 | 6.88 | 62.4 | 28.9 | 6.7 |
| C294 | 2 | 68 | 3.64 ± 0.203 | 1 | 1 | 0 | 4.34 | 1.38 | 1.09 | 2.7 | 5.64 | 4.35 | 61.1 | 27.1 | 8.5 |
| C300 | 1 | 55 | 4.27 ± 0.034 | 1 | 0 | 1 | 2.78 | 1.03 | 0.88 | 1.66 | 6.33 | 4.34 | 62.9 | 27.4 | 7.8 |
| C302 | 2 | 45 | 3.90 ± 0.081 | 0 | 1 | 0 | 6.33 | 1.65 | 1.4 | 4.04 | 4.15 | 7.09 | 45.3 | 44.4 | 7.6 |
| C303 | 1 | 58 | 3.91 ± 0.115 | 1 | 0 | 0 | 3.17 | 0.95 | 1.04 | 1.63 | 5.51 | 5.21 | 55.5 | 34.0 | 8.3 |
| C305 | 2 | 46 | 3.67 ± 0.726 | 1 | 0 | 0 | 6.87 | 1.26 | 1.46 | 4.27 | 8.42 | 8.33 | 85.4 | 10.4 | 3.7 |
| C315 | 2 | 64 | 3.84 ± 0.253 | 1 | 0 | 0 | 3.47 | 1.3 | 1.11 | 1.57 | 6.77 | 5.07 | 59.0 | 30.6 | 8.1 |
| C319 | 1 | 63 | 3.76 ± 0.176 | 0 | 0 | 1 | 2.92 | 0.56 | 1.41 | 1.06 | 4.4 | 5.38 | 70.8 | 19.5 | 7.6 |
| C321 | 1 | 47 | 4.41 ± 0.193 | 0 | 0 | 0 | 5.48 | 1.42 | 1.06 | 3.22 | 16.35 | 5.23 | 52.2 | 35.2 | 10.1 |
| C326 | 2 | 48 | 3.22 ± 0.045 | 1 | 1 | 0 | 5.52 | 2.79 | 1.09 | 3.36 | 5.93 | 5.81 | 58.7 | 31.5 | 6.7 |
| C333 | 1 | 57 | 3.00 ± 0.214 | 0 | 1 | 0 | 2.92 | 0.56 | 1.41 | 1.06 | 6.8 | 3.98 | 62.6 | 29.1 | 6.5 |
| C337 | 2 | 55 | 2.41 ± 0.049 | 0 | 0 | 0 | 3.7 | 1.22 | 1.3 | 2.13 | 5.58 | 8.35 | 55.8 | 31.7 | 7.3 |
| C343 | 2 | 55 | 3.21 ± 0.079 | 1 | 1 | 1 | 6.73 | 3.22 | 1.1 | 4.12 | 27.66 | 9 | 84.0 | 12.7 | 3.0 |
| C344 | 2 | 59 | 2.77 ± 0.117 | 1 | 1 | 0 | 4.45 | 3.56 | 0.88 | 1.95 | 5.4 | 4.65 | 45.2 | 46.9 | 5.8 |
| C370 | 1 | 52 | 4.34 ± 0.209 | 1 | 0 | 1 | 6.73 | 3.22 | 1.1 | 4.12 | 11 | 7.56 | 70.2 | 16.4 | 7.8 |
| C374 | 2 | 58 | 3.78 ± 1.122 | 0 | 0 | 0 | 4.57 | 1.18 | 1.22 | 2.76 | 8.27 | 6.41 | 51.0 | 37.8 | 7.8 |
| C381 | 1 | 70 | 3.01 ± 0.029 | 1 | 0 | 0 | 3.17 | 0.95 | 1.04 | 1.63 | 4.7 | 6.49 | 69.6 | 21.3 | 7.1 |
| C383 | 2 | 62 | 2.92 ± 0.104 | 1 | 1 | 0 | 4.23 | 1.57 | 1.33 | 2.06 | 5.69 | 5.67 | 57.7 | 34.7 | 7.4 |
| C389 | 1 | 48 | 3.50 ± 0.146 | 1 | 0 | 1 | 4.45 | 3.56 | 0.88 | 1.95 | 5.9 | 13.39 | 91.2 | 4.2 | 4.6 |
| C404 | 1 | 58 | 2.88 ± 0.108 | 0 | 1 | 1 | 3.29 | 1.1 | 1.2 | 1.42 | 5.87 | 6.3 | 45.6 | 40.0 | 11.1 |
| C405 | 2 | 66 | 4.33 ± 0.081 | 1 | 1 | 0 | 4.9 | 1.22 | 1.15 | 2.77 | 5.46 | 3.88 | 49.7 | 36.6 | 8.8 |
| C411 | 2 | 74 | 3.32 ± 0.010 | 0 | 0 | 1 | 4.48 | 1.51 | 0.96 | 2.63 | 6.43 | 6.15 | 57.1 | 29.9 | 12.2 |
| C412 | 2 | 57 | 3.35 ± 0.119 | 0 | 0 | 0 | 2.77 | 0.82 | 1.36 | 0.98 | 5.04 | 4.22 | 52.1 | 34.6 | 9.5 |
| C416 | 1 | 72 | 4.34 ± 0.096 | 1 | 0 | 1 | 3.98 | 0.6 | 1.39 | 2.8 | 9.88 | 6.71 | 70.8 | 15.5 | 12.4 |
| C417 | 1 | 54 | 3.03 ± 0.059 | 0 | 0 | 0 | 2.77 | 0.82 | 1.36 | 0.98 | 6.7 | 6.75 | 56.6 | 26.2 | 5.6 |
| C420 | 2 | 48 | 3.46 ± 0.102 | 0 | 0 | 1 | 3.2 | 1.26 | 1.1 | 1.48 | 6.9 | 10.63 | 77.1 | 15.5 | 5.8 |
| C422 | 1 | 72 | 3.30 ± 0.083 | 1 | 1 | 0 | 3.42 | 2.25 | 0.72 | 1.71 | 5.77 | 5.49 | 52.3 | 32.8 | 10.7 |
| C427 | 1 | 42 | 3.18 ± 0.264 | 1 | 1 | 0 | 4 | 1.82 | 0.88 | 1.94 | 5.33 | 6.58 | 53.5 | 30.2 | 8.8 |
| C433 | 2 | 54 | 4.14 ± 0.173 | 1 | 0 | 0 | 3.42 | 2.25 | 0.72 | 1.71 | 7.7 | 5.62 | 62.3 | 25.6 | 8.2 |
| C436 | 1 | 39 | 3.98 ± 0.063 | 0 | 0 | 0 | 5.89 | 3.55 | 0.84 | 3.34 | 5.17 | 6.28 | 46.3 | 43.8 | 8.0 |
| C437 | 2 | 48 | 3.30 ± 0.041 | 1 | 0 | 1 | 3.9 | 1.4 | 0.88 | 2.02 | 8.41 | 7.42 | 45.0 | 42.9 | 6.6 |
| C446 | 2 | 54 | 3.88 ± 0.090 | 0 | 0 | 0 | 5.7 | 4.21 | 1.03 | 2.77 | 5.45 | 5.45 | 47.3 | 41.7 | 6.6 |
| C448 | 1 | 78 | 3.63 ± 0.057 | 1 | 0 | 1 | 4.6 | 1.14 | 0.75 | 3.43 | 5.36 | 5.15 | 67.4 | 22.7 | 6.8 |
| C451 | 2 | 44 | 3.72 ± 0.053 | 0 | 0 | 1 | 6.65 | 1.97 | 1.29 | 3.76 | 20.39 | 6.9 | 39.6 | 42.0 | 8.1 |
| C455 | 1 | 62 | 3.33 ± 0.025 | 1 | 1 | 0 | 5.7 | 4.21 | 1.03 | 2.77 | 7.17 | 7.54 | 77.7 | 12.9 | 5.6 |
| C458 | 1 | 67 | 4.15 ± 0.074 | 1 | 1 | 0 | 6.16 | 2.57 | 0.92 | 3.38 | 6.78 | 4.36 | 46.3 | 38.5 | 7.3 |
| C465 | 2 | 63 | 3.34 ± 0.135 | 1 | 1 | 0 | 6.75 | 1.48 | 1.37 | 4.57 | 6.21 | 8.63 | 73.9 | 20.0 | 5.4 |
| C474 | 1 | 46 | 3.72 ± 0.211 | 1 | 0 | 1 | 4.14 | 1.59 | 0.82 | 2.23 | 5.5 | 7.22 | 49.9 | 36.3 | 8.9 |
| C476 | 2 | 53 | 4.06 ± 0.124 | 1 | 1 | 0 | 3.57 | 2.15 | 0.91 | 1.63 | 5.39 | 7.43 | 49.9 | 40.1 | 7.9 |
| C485 | 1 | 49 | 3.49 ± 0.115 | 1 | 1 | 1 | 2.9 | 0.51 | 1.54 | 1.03 | 5.47 | 4.34 | 59.4 | 27.9 | 10.8 |
| C487 | 1 | 71 | 3.72 ± 0.013 | 1 | 0 | 0 | 2.77 | 1.18 | 1.14 | 1.07 | 5.81 | 5.18 | 55.2 | 28.6 | 7.1 |
| C488 | 1 | 61 | 3.18 ± 0.104 | 0 | 0 | 0 | 5.61 | 1.13 | 1.03 | 3.54 | 3.97 | 6.2 | 51.3 | 39.2 | 6.3 |
| C489 | 2 | 56 | 3.81 ± 0.520 | 0 | 1 | 0 | 5.1 | 1.92 | 1.26 | 2.9 | 5.39 | 6.07 | 48.1 | 34.4 | 8.9 |
| C492 | 1 | 49 | 3.65 ± 0.028 | 1 | 1 | 0 | 4.21 | 1.3 | 1.1 | 2.28 | 4.88 | 7.19 | 45.8 | 41.9 | 6.8 |
| C496 | 2 | 61 | 3.90 ± 0.080 | 1 | 1 | 0 | 6.65 | 1.13 | 1.46 | 4.1 | 6.02 | 4.36 | 49.5 | 40.1 | 6.9 |
| C497 | 2 | 62 | 3.90 ± 0.006 | 1 | 1 | 0 | 4.9 | 2.18 | 0.93 | 3.38 | 5.52 | 6.39 | 65.3 | 26.4 | 7.0 |
| C507 | 2 | 58 | 3.77 ± 0.007 | 0 | 0 | 1 | 4.21 | 1.2 | 0.66 | 3.19 | 8.31 | 6.89 | 67.9 | 22.5 | 7.4 |
| C512 | 1 | 65 | 3.68 ± 0.016 | 1 | 1 | 1 | 4.48 | 2.96 | 0.74 | 2.75 | 14.9 | 8.95 | 69.2 | 19.8 | 7.9 |
| C513 | 1 | 47 | 3.51 ± 0.049 | 0 | 0 | 0 | 4.21 | 0.73 | 1.38 | 2.33 | 5.01 | 4.94 | 65.8 | 24.7 | 7.7 |
| C517 | 2 | 56 | 3.50 ± 0.116 | 0 | 0 | 1 | 4.07 | 2.14 | 0.77 | 2.27 | 6.34 | 4.79 | 62.8 | 26.7 | 8.6 |
| C529 | 1 | 46 | 3.75 ± 0.184 | 1 | 0 | 1 | 3.29 | 1.02 | 0.96 | 2.26 | 14.04 | 7.79 | 61.4 | 25.0 | 11.3 |
| C534 | 2 | 74 | 3.74 ± 0.032 | 0 | 0 | 1 | 3.81 | 0.64 | 1.68 | 1.54 | 5.97 | 6.97 | 53.8 | 37.3 | 7.3 |
| C538 | 2 | 49 | 3.97 ± 0.012 | 0 | 0 | 1 | 4.8 | 3.32 | 0.97 | 2.42 | 7.12 | 8.03 | 58.8 | 29.8 | 8.3 |
| C544 | 2 | 46 | 3.76 ± 0.043 | 0 | 0 | 1 | 3.55 | 1.18 | 0.86 | 2.18 | 6.91 | 5.08 | 53.5 | 34.1 | 8.5 |
| C546 | 1 | 54 | 3.84 ± 0.073 | 0 | 0 | 1 | 8.04 | 1.64 | 1.17 | 5.88 | 22.64 | 11.77 | 73.2 | 21.5 | 4.7 |
| C566 | 1 | 54 | 4.38 ± 0.030 | 1 | 0 | 0 | 4.82 | 5.85 | 0.77 | 1.71 | 3.93 | 7.5 | 61.2 | 30.9 | 6.9 |
| C573 | 2 | 51 | 3.68 ± 0.011 | 0 | 0 | 0 | 4.79 | 0.57 | 2.03 | 2.44 | 7.03 | 8.59 | 79.7 | 15.7 | 4.0 |
| C577 | 2 | 60 | 3.89 ± 0.031 | 0 | 0 | 0 | 4.79 | 1.63 | 1.41 | 2.74 | 5.99 | 4.95 | 57.0 | 29.5 | 9.9 |
| C581 | 2 | 62 | 3.67 ± 0.186 | 0 | 0 | 0 | 4.9 | 1.24 | 1.7 | 2.65 | 4.42 | 5.83 | 55.2 | 34.5 | 8.7 |
| C584 | 2 | 59 | 3.71 ± 0.006 | 1 | 1 | 0 | 5.82 | 1.62 | 1.54 | 3.56 | 11.7 | 14.62 | 81.2 | 12.1 | 6.4 |
| C591 | 2 | 54 | 2.98 ± 0.122 | 1 | 0 | 0 | 3.26 | 0.9 | 1.06 | 1.77 | 10.7 | 17.45 | 82.4 | 12.3 | 4.8 |
| C699 | 1 | 62 | 3.35 ± 0.084 | 0 | 1 | 0 | 4.9 | 1.24 | 1.7 | 2.65 | 5.48 | 5.79 | 55.3 | 31.1 | 10.4 |
| C702 | 2 | 67 | 3.07 ± 0.034 | 1 | 0 | 1 | 3.7 | 1.21 | 1.76 | 1.17 | 6.25 | 9.87 | 72.6 | 20.7 | 5.6 |
| C715 | 2 | 47 | 3.06 ± 0.196 | 0 | 1 | 0 | 4.7 | 2.46 | 1.25 | 2.33 | 5.43 | 6.41 | 61.6 | 32.1 | 4.5 |
| C725 | 1 | 34 | 4.35 ± 0.079 | 1 | 0 | 0 | 4.06 | 0.92 | 1 | 2.58 | 5.92 | 15.52 | 82.6 | 12.4 | 3.3 |
| C727 | 2 | 51 | 3.26 ± 0.190 | 1 | 0 | 1 | 4.68 | 1.45 | 0.88 | 2.54 | 7.37 | 5.12 | 71.1 | 21.3 | 6.8 |
| C730 | 1 | 63 | 4.54 ± 0.134 | 0 | 0 | 0 | 3.4 | 0.69 | 1.04 | 1.54 | 7.24 | 7.6 | 45.1 | 38.6 | 13.0 |
| C736 | 1 | 55 | 4.38 ± 0.224 | 1 | 0 | 1 | 5.48 | 1.6 | 0.86 | 3.49 | 8.07 | 6.58 | 52.3 | 33.6 | 12.3 |
| C737 | 1 | 47 | 4.64 ± 0.201 | 0 | 1 | 0 | 8.16 | 0.79 | 1.12 | 5.2 | 6.07 | 4.93 | 74.2 | 16.4 | 5.1 |
| C740 | 1 | 61 | 3.26 ± 0.068 | 1 | 1 | 0 | 4.59 | 1.66 | 0.91 | 2.41 | 5.48 | 4.24 | 60.4 | 29.5 | 8.0 |
| C764 | 1 | 51 | 4.01 ± 0.148 | 1 | 0 | 1 | 3.17 | 1.1 | 0.91 | 1.41 | 5.92 | 4.88 | 50.6 | 26.0 | 19.3 |
| C774 | 1 | 75 | 4.74 ± 0.142 | 1 | 0 | 1 | 2.96 | 1.11 | 0.63 | 1.51 | 5.9 | 6.94 | 62.1 | 18.4 | 15.0 |
| C782 | 2 | 48 | 4.40 ± 0.064 | 1 | 1 | 0 | 3.61 | 1.56 | 0.92 | 1.03 | 5.05 | 7.37 | 64.3 | 27.8 | 5.7 |
| C790 | 2 | 75 | 4.40 ± 0.205 | 0 | 0 | 0 | 4.62 | 5.28 | 0.71 | 1.68 | 5.5 | 5.68 | 60.2 | 29.9 | 7.4 |
| C799 | 2 | 46 | 4.41 ± 0.505 | 1 | 0 | 0 | 3.76 | 1.06 | 1.19 | 2 | 5.98 | 5.86 | 52.0 | 35.0 | 8.9 |
| C807 | 1 | 58 | 4.88 ± 0.367 | 1 | 0 | 0 | 5.8 | 1.29 | 1.1 | 3.87 | 4.9 | 4.71 | 55.0 | 32.3 | 8.3 |
| C809 | 1 | 70 | 4.65 ± 0.002 | 0 | 0 | 0 | 4.23 | 0.76 | 1.49 | 2.11 | 5.48 | 4.94 | 75.5 | 13.2 | 8.7 |
| C815 | 2 | 69 | 3.59 ± 0.031 | 1 | 0 | 0 | 3.92 | 1.48 | 0.97 | 2.22 | 5.17 | 6.83 | 57.1 | 29.1 | 8.9 |
| C821 | 2 | 61 | 3.01 ± 0.090 | 1 | 1 | 0 | 3.39 | 1.54 | 0.88 | 1.74 | 4.65 | 5.95 | 54.8 | 33.6 | 6.9 |
| C822 | 1 | 46 | 3.35 ± 0.068 | 1 | 1 | 0 | 4.23 | 0.76 | 1.49 | 2.11 | 3.78 | 6.71 | 63.9 | 27.3 | 6.3 |
| C824 | 1 | 53 | 4.96 ± 0.182 | 1 | 1 | 1 | 4.45 | 2.16 | 1.05 | 2.26 | 15.8 | 7.44 | 79.6 | 14.9 | 3.6 |
| C836 | 1 | 59 | 4.94 ± 0.088 | 1 | 1 | 1 | 4.2 | 1.19 | 0.98 | 2.8 | 5.74 | 7.36 | 43.8 | 49.5 | 6.0 |
| C837 | 1 | 53 | 4.62 ± 0.343 | 0 | 0 | 0 | 4.05 | 1.15 | 1.2 | 2.24 | 5.8 | 6.73 | 70.7 | 21.5 | 5.5 |
| C839 | 1 | 58 | 3.35 ± 0.063 | 0 | 0 | 1 | 3.64 | 1.21 | 0.83 | 2.43 | 8.45 | 7.97 | 70.0 | 20.6 | 7.4 |
| C845 | 1 | 56 | 4.87 ± 0.034 | 1 | 1 | 0 | 2.51 | 1.96 | 0.65 | 1.17 | 6.09 | 9.04 | 71.2 | 20.5 | 7.2 |
| C851 | 2 | 55 | 4.69 ± 0.203 | 0 | 1 | 0 | 3.61 | 2.4 | 0.68 | 1.95 | 5.46 | 6.13 | 48.5 | 35.9 | 10.3 |
| C857 | 1 | 47 | 5.06 ± 0.020 | 0 | 1 | 0 | 4.77 | 1.82 | 1.02 | 2.71 | 6.25 | 8.3 | 57.2 | 29.0 | 10.4 |
| C858 | 1 | 55 | 3.88 ± 0.029 | 0 | 0 | 0 | 3.61 | 2.4 | 0.68 | 1.95 | 7.56 | 7.86 | 70.1 | 20.6 | 5.2 |
| C881 | 2 | 55 | 4.80 ± 0.186 | 0 | 0 | 1 | 2.95 | 1.91 | 0.8 | 1.31 | 3.59 | 13.39 | 76.0 | 15.4 | 8.1 |
| C888 | 1 | 63 | 4.39 ± 0.147 | 1 | 1 | 0 | 4.72 | 0.78 | 1.45 | 2.78 | 5.51 | 7.6 | 63.2 | 23.2 | 11.2 |
| C898 | 1 | 59 | 4.37 ± 0.429 | 1 | 1 | 0 | 3.17 | 1.83 | 0.84 | 1.7 | 5.38 | 5.9 | 82.4 | 13.7 | 3.7 |
| C899 | 1 | 38 | 4.75 ± 0.404 | 0 | 0 | 0 | 2.57 | 0.72 | 1.02 | 1.24 | 4.9 | 12.29 | 49.0 | 11.0 | 8.1 |
| C904 | 2 | 49 | 4.61 ± 0.230 | 1 | 0 | 1 | 5.94 | 1.76 | 1.46 | 3.6 | 6.59 | 3.76 | 64.9 | 26.9 | 5.1 |
| C914 | 1 | 48 | 3.34 ± 0.080 | 0 | 0 | 0 | 3.76 | 2.44 | 0.85 | 2 | 4.82 | 5.46 | 67.6 | 20.7 | 6.6 |
| C921 | 1 | 51 | 4.09 ± 0.314 | 0 | 0 | 0 | 5.32 | 5.32 | 1.16 | 3.58 | 29.57 | 7.29 | 70.6 | 19.8 | 6.7 |
| C932 | 2 | 51 | 3.73 ± 0.181 | 0 | 1 | 0 | 4.37 | 1.13 | 1.55 | 2 | 5.78 | 4.98 | 41.8 | 34.9 | 9.6 |
| C936 | 1 | 53 | 3.28 ± 0.050 | 0 | 0 | 1 | 4.44 | 2.66 | 0.9 | 2.47 | 4.05 | 6.58 | 50.2 | 37.2 | 7.9 |
| C945 | 2 | 69 | 3.85 ± 0.408 | 0 | 1 | 0 | 4.16 | 1.13 | 1.26 | 2.18 | 6.63 | 5.93 | 54.5 | 32.5 | 9.8 |
| C948 | 1 | 60 | 3.87 ± 0.265 | 0 | 0 | 1 | 4.05 | 1.01 | 1.16 | 2.33 | 9.12 | 9.26 | 67.2 | 19.5 | 4.5 |
| C953 | 1 | 67 | 3.54 ± 0.131 | 1 | 0 | 1 | 4.37 | 1.13 | 1.55 | 2 | 7.92 | 3.854 | 77.6 | 42.8 | 10.9 |
| C961 | 1 | 52 | 3.68 ± 0.059 | 1 | 1 | 1 | 3.92 | 1.97 | 0.98 | 1.88 | 15.36 | 4.05 | 54.1 | 31.9 | 8.4 |
| C963 | 1 | 62 | 3.18 ± 0.226 | 1 | 0 | 1 | 5.3 | 1.91 | 1.21 | 3.49 | 7.25 | 6.37 | 70.3 | 22.8 | 5.3 |
| C967 | 2 | 60 | 3.11 ± 0.151 | 0 | 0 | 0 | 3.99 | 1.52 | 1.73 | 1.64 | 4.9 | 4.51 | 47.9 | 37.3 | 11.3 |
| C968 | 1 | 69 | 3.08 ± 0.024 | 0 | 0 | 0 | 2.65 | 0.77 | 1.13 | 1.08 | 4.78 | 6.09 | 50.1 | 38.3 | 7.7 |
| C969 | 1 | 64 | 3.61 ± 0.035 | 1 | 0 | 0 | 5.31 | 2.13 | 1.16 | 3.25 | 5.31 | 6.77 | 56.0 | 34.1 | 7.5 |
| C970 | 1 | 49 | 3.69 ± 0.146 | 0 | 0 | 0 | 2.88 | 1.9 | 0.8 | 1.24 | 6.23 | 7.52 | 59.2 | 21.3 | 6.4 |
| C984 | 1 | 56 | 3.36 ± 0.043 | 0 | 0 | 0 | 3.47 | 0.94 | 1.55 | 1.51 | 5.14 | 8.89 | 64.0 | 22.9 | 10.5 |
| C1011 | 1 | 62 | 3.79 ± 0.101 | 1 | 0 | 0 | 2.88 | 1.9 | 0.8 | 1.24 | 4.9 | 6.32 | 62.8 | 27.5 | 5.9 |
| C1041 | 1 | 50 | 4.27 ± 0.239 | 1 | 1 | 0 | 4.98 | 2.65 | 0.99 | 2.9 | 7.92 | 10.05 | 68.5 | 22.1 | 6.8 |
| C1126 | 1 | 55 | 4.17 ± 0.525 | 0 | 0 | 0 | 3.93 | 0.84 | 1.15 | 2.23 | 4.46 | 5.7 | 74.7 | 18.9 | 4.9 |
| C1127 | 1 | 35 | 3.81 ± 0.055 | 0 | 0 | 0 | 3.97 | 0.9 | 1.44 | 1.95 | 6.71 | 8.68 | 78.1 | 18.2 | 2.3 |
| C1128 | 1 | 55 | 3.77 ± 0.176 | 1 | 1 | 0 | 2.99 | 1.03 | 0.64 | 1.79 | 5.69 | 7.58 | 46.0 | 32.8 | 13.5 |
| C1129 | 2 | 65 | 4.27 ± 0.076 | 1 | 0 | 0 | 4.68 | 2.76 | 1.26 | 2.54 | 7.12 | 8.53 | 73.3 | 16.5 | 7.2 |
| C1132 | 1 | 49 | 4.20 ± 0.121 | 1 | 0 | 0 | 3.48 | 1.13 | 1.21 | 1.89 | 5.27 | 8.07 | 76.0 | 15.5 | 6.9 |
| C1134 | 1 | 67 | 4.44 ± 0.037 | 1 | 0 | 0 | 2.99 | 1.03 | 0.64 | 1.79 | 5.43 | 7.39 | 64.8 | 23.3 | 10.3 |
| C1136 | 2 | 55 | 4.75 ± 0.273 | 1 | 0 | 0 | 3.54 | 1.02 | 1.07 | 2.07 | 6.35 | 7.13 | 50.6 | 36.6 | 8.1 |
| C1139 | 1 | 77 | 4.31 ± 0.096 | 1 | 0 | 0 | 3.74 | 0.66 | 1 | 2.03 | 8.37 | 4.2 | 62.1 | 23.6 | 9.0 |
| C1145 | 1 | 51 | 4.47 ± 0.020 | 1 | 0 | 0 | 2.94 | 1.14 | 0.79 | 1.42 | 5.22 | 4.98 | 46.4 | 40.4 | 8.6 |
| C1148 | 2 | 50 | 4.57 ± 0.165 | 1 | 1 | 1 | 3.74 | 0.66 | 1 | 2.03 | 5.78 | 5.11 | 63.6 | 26.0 | 6.3 |
| C1149 | 1 | 46 | 4.09 ± 0.039 | 0 | 0 | 0 | 3.9 | 1.27 | 1.37 | 2.26 | 6.77 | 5.26 | 55.5 | 30.0 | 10.8 |
| C1150 | 1 | 63 | 4.41 ± 0.165 | 1 | 0 | 0 | 4.75 | 0.82 | 1.27 | 3.08 | 7.96 | 12.9 | 81.1 | 12.0 | 5.7 |
| C1152 | 1 | 62 | 4.17 ± 0.295 | 0 | 0 | 0 | 4.21 | 1.72 | 0.94 | 2.62 | 5.22 | 6.61 | 57.9 | 31.5 | 5.7 |
| C1155 | 2 | 55 | 4.33 ± 0.173 | 1 | 0 | 0 | 3.32 | 1.26 | 0.89 | 1.68 | 5.99 | 5.06 | 42.3 | 50.4 | 4.7 |
| C1156 | 1 | 48 | 4.22 ± 0.022 | 1 | 0 | 0 | 3.96 | 2.19 | 0.67 | 1.9 | 4.83 | 7.36 | 72.8 | 18.2 | 7.1 |
| C1157 | 1 | 49 | 4.56 ± 0.211 | 1 | 0 | 0 | 5.36 | 1.75 | 1.69 | 2.79 | 15.08 | 10.31 | 85.6 | 8.7 | 5.2 |
| C1159 | 1 | 63 | 4.36 ± 0.123 | 0 | 0 | 0 | 4.87 | 1.27 | 0.95 | 3.05 | 4.91 | 5.9 | 66.3 | 22.9 | 7.6 |
| C1160 | 1 | 59 | 3.87 ± 0.215 | 1 | 0 | 0 | 4.37 | 1.97 | 0.98 | 2.25 | 6.49 | 7.73 | 65.3 | 25.4 | 7.1 |
| C1164 | 1 | 66 | 4.26 ± 0.134 | 1 | 0 | 0 | 3.2 | 0.81 | 1.16 | 1.55 | 6.81 | 3.97 | 63.5 | 22.7 | 10.1 |
| C1167 | 2 | 71 | 4.41 ± 0.017 | 1 | 0 | 1 | 4.6 | 0.98 | 2.38 | 1.54 | 7.21 | 8.61 | 76.9 | 15.3 | 7.0 |
| C1171 | 1 | 47 | 4.87 ± 0.057 | 1 | 0 | 0 | 3.62 | 0.93 | 1.36 | 1.75 | 5.72 | 5.87 | 62.0 | 28.6 | 7.5 |
| C1173 | 2 | 64 | 5.16 ± 0.026 | 1 | 0 | 1 | 4.4 | 1.15 | 1.09 | 2.56 | 10.13 | 5.98 | 68.7 | 22.7 | 6.2 |
| C1174 | 2 | 47 | 4.49 ± 0.375 | 1 | 1 | 0 | 5.18 | 1.6 | 1.28 | 2.82 | 5.94 | 10.31 | 67.9 | 20.5 | 7.9 |
| C1175 | 1 | 57 | 4.76 ± 0.091 | 1 | 0 | 0 | 4.55 | 1.28 | 1.38 | 2.45 | 7.12 | 10.03 | 80.3 | 14.4 | 4.5 |
| C1176 | 1 | 57 | 4.85 ± 0.273 | 1 | 0 | 0 | 2.61 | 0.98 | 1.09 | 1.05 | 5.64 | 4.76 | 65.3 | 22.7 | 10.1 |
| C1178 | 2 | 48 | 4.93 ± 0.195 | 1 | 1 | 0 | 4.61 | 1.71 | 0.86 | 2.63 | 6.13 | 8.31 | 68.0 | 26.0 | 3.7 |
| C1182 | 1 | 45 | 4.18 ± 0.012 | 0 | 0 | 0 | 3.13 | 1.27 | 0.8 | 1.46 | 8.06 | 5.32 | 52.6 | 20.9 | 12.4 |
| C1183 | 2 | 56 | 4.21 ± 0.299 | 1 | 0 | 0 | 5.83 | 1.01 | 1.48 | 3.96 | 8.16 | 6.32 | 61.1 | 27.5 | 7.4 |
| C1184 | 1 | 46 | 4.39 ± 0.111 | 1 | 0 | 0 | 4.08 | 1.35 | 1.08 | 2 | 9.49 | 5.7 | 53.7 | 30.5 | 11.2 |
| C1194 | 1 | 55 | 4.24 ± 0.186 | 1 | 0 | 0 | 3.89 | 0.87 | 1.12 | 2.32 | 6.71 | 4.53 | 59.8 | 27.8 | 7.9 |
| C1196 | 1 | 64 | 4.00 ± 0.150 | 0 | 0 | 0 | 3.08 | 1.6 | 0.98 | 1.46 | 6.16 | 8.35 | 60.8 | 28.3 | 7.9 |
| C1199 | 2 | 68 | 4.00 ± 0.301 | 1 | 0 | 0 | 3.85 | 0.98 | 1.41 | 2.13 | 5.88 | 6.77 | 40.9 | 45.2 | 8.1 |
| C1202 | 1 | 57 | 5.12 ± 0.160 | 1 | 0 | 0 | 4.27 | 1.92 | 0.86 | 2.5 | 5.95 | 5.91 | 57.5 | 24.2 | 11.2 |
| C1204 | 1 | 45 | 4.05 ± 0.366 | 0 | 0 | 0 | 4.87 | 0.87 | 1.16 | 3.17 | 5.4 | 4.95 | 53.1 | 30.7 | 8.3 |
| C1205 | 2 | 58 | 4.01 ± 0.220 | 0 | 0 | 0 | 2.5 | 0.69 | 0.77 | 1.74 | 4.69 | 9.36 | 77.8 | 15.7 | 4.9 |
| C1208 | 1 | 53 | 3.89 ± 0.088 | 1 | 0 | 0 | 4.12 | 1.12 | 0.96 | 2.6 | 5.29 | 5.65 | 43.0 | 40.0 | 10.8 |
| C1209 | 1 | 71 | 4.40 ± 0.541 | 0 | 0 | 0 | 5.07 | 1.3 | 0.89 | 3.28 | 5.22 | 6.2 | 55.0 | 34.7 | 8.5 |
| C1211 | 1 | 71 | 3.72 ± 0.198 | 1 | 0 | 0 | 4.08 | 1.43 | 1.07 | 2.17 | 4.9 | 8.46 | 74.9 | 13.8 | 9.5 |
| C1215 | 1 | 61 | 3.81 ± 0.108 | 1 | 0 | 0 | 3.21 | 0.74 | 1.04 | 1.6 | 5.06 | 7.72 | 68.0 | 21.0 | 8.2 |
| C1216 | 2 | 53 | 3.28 ± 0.014 | 1 | 0 | 1 | 3.22 | 0.84 | 1.18 | 1.57 | 5.51 | 6.6 | 46.2 | 45.2 | 7.0 |
| C1218 | 2 | 56 | 2.91 ± 0.055 | 0 | 0 | 0 | 5.4 | 1.39 | 2.03 | 2.68 | 5.94 | 4.41 | 56.5 | 32.9 | 8.2 |
| C1220 | 2 | 73 | 3.72 ± 0.324 | 0 | 0 | 0 | 3.72 | 1.12 | 0.91 | 2.15 | 5.4 | 5.21 | 58.7 | 30.3 | 7.9 |
| C1222 | 2 | 42 | 3.43 ± 0.222 | 0 | 0 | 0 | 5.33 | 1.75 | 3.29 | 1.41 | 5.98 | 14.71 | 60.6 | 20.7 | 6.7 |
| C1225 | 1 | 66 | 3.98 ± 0.227 | 0 | 1 | 0 | 4.14 | 1.7 | 0.93 | 2.22 | 5.98 | 5.92 | 60.0 | 27.7 | 9.6 |
| C1226 | 1 | 48 | 3.17 ± 0.096 | 0 | 1 | 0 | 6.52 | 5.21 | 1.03 | 3.19 | 6.33 | 9.94 | 70.1 | 18.8 | 7.8 |
| C1227 | 2 | 45 | 3.94 ± 0.051 | 0 | 1 | 1 | 5.49 | 2.35 | 1.82 | 2.81 | 7.22 | 13.64 | 79.9 | 16.1 | 3.6 |
| C1228 | 2 | 62 | 3.72 ± 0.107 | 1 | 1 | 0 | 5.88 | 1.5 | 1.5 | 3.51 | 5.41 | 6.29 | 50.9 | 38.0 | 7.6 |
| C1230 | 2 | 47 | 1.63 ± 0.165 | 1 | 0 | 1 | 3.32 | 1.51 | 0.97 | 1.69 | 7.21 | 7.74 | 70.7 | 21.3 | 6.1 |
| C1231 | 1 | 56 | 3.72 ± 0.070 | 1 | 0 | 0 | 3.84 | 2.03 | 0.69 | 2.29 | 11.67 | 10.33 | 74.4 | 16.6 | 6.1 |
| C1234 | 2 | 59 | 4.56 ± 0.311 | 1 | 0 | 0 | 4.75 | 2.7 | 0.94 | 2.63 | 6.29 | 8.76 | 63.6 | 26.6 | 7.5 |
| C1237 | 2 | 51 | 3.86 ± 0.033 | 1 | 0 | 0 | 4.52 | 0.7 | 1.94 | 2.28 | 5.58 | 4.12 | 62.6 | 26.2 | 6.6 |
| C1239 | 1 | 55 | 3.49 ± 0.420 | 0 | 0 | 0 | 3.99 | 0.75 | 1.37 | 2.21 | 5.07 | 6.35 | 53.2 | 37.5 | 7.6 |
| C1241 | 1 | 62 | 3.82 ± 0.471 | 0 | 0 | 0 | 4.74 | 0.86 | 1.19 | 2.84 | 5.67 | 6.21 | 61.5 | 25.1 | 9.3 |
| C1242 | 1 | 50 | 3.62 ± 0.170 | 1 | 0 | 0 | 3.19 | 0.98 | 1.1 | 1.16 | 5.09 | 7.57 | 64.6 | 29.5 | 5.0 |
| C1247 | 1 | 69 | 3.69 ± 0.076 | 0 | 1 | 0 | 6.69 | 1.58 | 0.85 | 4.29 | 5.69 | 4.97 | 53.1 | 29.6 | 14.9 |
| C1250 | 2 | 63 | 2.05 ± 0.017 | 1 | 1 | 1 | 5.06 | 1.94 | 1.41 | 2.53 | 5.5 | 7.85 | 57.1 | 31.2 | 6.2 |
| C1251 | 1 | 63 | 3.65 ± 0.183 | 0 | 0 | 0 | 4.8 | 0.73 | 1.23 | 3.01 | 5.84 | 3.51 | 61.3 | 29.6 | 7.1 |
| C1252 | 1 | 45 | 4.06 ± 0.512 | 1 | 1 | 1 | 4.9 | 1.79 | 1.06 | 3.15 | 5.86 | 7.2 | 71.5 | 19.0 | 7.9 |
| C1254 | 2 | 57 | 2.96 ± 0.068 | 1 | 0 | 0 | 3.33 | 1.49 | 1.01 | 1.78 | 4.88 | 6.69 | 47.7 | 39.3 | 7.9 |
| C1255 | 1 | 57 | 2.71 ± 0.250 | 0 | 0 | 0 | 3.55 | 2.08 | 1.14 | 1.81 | 4.44 | 6.73 | 59.1 | 34.2 | 5.3 |
| C1258 | 2 | 49 | 4.49 ± 0.283 | 1 | 1 | 0 | 4.64 | 1.96 | 1.01 | 2.6 | 4.72 | 5.84 | 50.7 | 37.3 | 7.9 |
| C1259 | 1 | 74 | 4.74 ± 0.008 | 0 | 0 | 0 | 2.94 | 0.67 | 0.87 | 1.48 | 4.67 | 6.21 | 65.2 | 23.3 | 8.1 |
| C1260 | 1 | 64 | 4.94 ± 0.058 | 1 | 0 | 1 | 4.49 | 0.71 | 1.6 | 2.26 | 8.05 | 6.51 | 65.4 | 22.6 | 10.1 |
| C1262 | 1 | 68 | 4.71 ± 0.219 | 1 | 0 | 0 | 5.1 | 1.04 | 1.48 | 2.94 | 5.66 | 6.1 | 49.5 | 37.4 | 6.9 |
| C1303 | 2 | 42 | 4.76 ± 1.006 | 1 | 0 | 0 | 4.8 | 1.66 | 1.59 | 2.35 | 4.93 | 7.75 | 55.5 | 32.4 | 8.0 |
| C1307 | 2 | 46 | 4.08 ± 0.259 | 1 | 0 | 1 | 4.6 | 0.68 | 1.23 | 2.67 | 7.28 | 5.61 | 56.5 | 31.4 | 9.6 |
| C1308 | 1 | 62 | 4.48 ± 0.071 | 0 | 0 | 0 | 4.19 | 1.78 | 0.93 | 2.21 | 5.19 | 6.76 | 63.6 | 22.0 | 11.5 |
| C1309 | 2 | 64 | 4.53 ± 0.515 | 1 | 1 | 0 | 7.23 | 1.38 | 2.23 | 3.69 | 5.9 | 4.8 | 49.8 | 35.6 | 11.3 |
| C1311 | 1 | 60 | 4.36 ± 0.346 | 1 | 1 | 0 | 3.95 | 1.68 | 1.08 | 1.88 | 5.46 | 5.18 | 59.7 | 28.8 | 9.1 |
| C1313 | 1 | 55 | 4.89 ± 0.007 | 1 | 1 | 0 | 4.25 | 4.83 | 0.94 | 1.61 | 5.66 | 6.69 | 63.8 | 21.8 | 8.8 |
| C1328 | 1 | 64 | 4.78 ± 0.210 | 1 | 0 | 0 | 5.57 | 1.83 | 0.95 | 3.3 | 4.94 | 8.02 | 61.8 | 27.3 | 5.7 |
| C1329 | 1 | 56 | 3.77 ± 0.126 | 1 | 0 | 0 | 5.41 | 1.38 | 1.07 | 3.22 | 5.41 | 4.55 | 61.3 | 28.6 | 8.4 |
| C1330 | 1 | 73 | 5.05 ± 0.142 | 1 | 0 | 0 | 5.07 | 1.08 | 1.08 | 3.06 | 5.55 | 5.93 | 57.3 | 29.7 | 4.7 |
| C1332 | 2 | 52 | 3.71 ± 0.576 | 1 | 0 | 1 | 5.58 | 1.53 | 1.37 | 3.11 | 10.03 | 5.56 | 46.0 | 39.0 | 9.2 |
| C1335 | 1 | 73 | 4.72 ± 0.259 | 1 | 0 | 0 | 4.73 | 0.81 | 1 | 2.88 | 5 | 10.42 | 69.5 | 16.9 | 9.9 |
| C1336 | 2 | 60 | 3.85 ± 0.061 | 1 | 0 | 0 | 3.02 | 0.91 | 1.32 | 1.06 | 6.22 | 9.57 | 74.8 | 15.6 | 7.9 |
| C1345 | 1 | 69 | 3.94 ± 0.011 | 1 | 0 | 0 | 4.78 | 0.89 | 1.3 | 2.48 | 5.71 | 4.76 | 62.8 | 26.3 | 6.9 |
| C1348 | 2 | 46 | 4.29 ± 0.144 | 1 | 0 | 0 | 3.95 | 1.02 | 1.5 | 1.68 | 5.68 | 5.08 | 66.5 | 23.0 | 7.7 |
| C1349 | 2 | 66 | 3.97 ± 0.226 | 1 | 0 | 0 | 2.54 | 0.73 | 1.25 | 0.89 | 4.92 | 6.78 | 57.1 | 32.0 | 7.1 |
| C1351 | 2 | 53 | 4.33 ± 0.093 | 1 | 0 | 0 | 3.86 | 0.49 | 1.18 | 1.86 | 4.77 | 7.05 | 56.6 | 32.1 | 7.1 |
| C1352 | 1 | 70 | 4.70 ± 0.030 | 1 | 0 | 0 | 4.05 | 1.54 | 1.02 | 2.44 | 11.62 | 7.52 | 64.1 | 23.7 | 9.7 |
| C1353 | 1 | 75 | 4.10 ± 0.131 | 0 | 0 | 0 | 4.69 | 1.37 | 0.88 | 2.7 | 6.37 | 4.91 | 55.2 | 35.8 | 5.7 |
| C1355 | 2 | 67 | 3.78 ± 0.044 | 1 | 1 | 1 | 2.54 | 0.73 | 1.25 | 0.89 | 6.66 | 5.12 | 45.9 | 38.5 | 11.5 |
| C1356 | 1 | 79 | 4.41 ± 0.267 | 1 | 0 | 0 | 3.92 | 0.83 | 1.2 | 2.08 | 5.91 | 6.38 | 58.0 | 25.7 | 10.0 |
| C1359 | 2 | 72 | 4.14 ± 0.162 | 1 | 0 | 0 | 4.11 | 0.92 | 1.27 | 2.07 | 5.37 | 4.67 | 53.5 | 33.6 | 8.1 |
| C1360 | 1 | 54 | 4.12 ± 0.074 | 0 | 0 | 0 | 4.82 | 0.45 | 3.06 | 2.57 | 5.72 | 4.97 | 80.9 | 12.5 | 6.0 |
| C1361 | 1 | 54 | 4.53 ± 0.290 | 1 | 0 | 0 | 5.34 | 1.91 | 1.34 | 2.97 | 5.45 | 7.22 | 72.4 | 17.6 | 6.9 |
| C1362 | 2 | 55 | 3.04 ± 0.684 | 0 | 0 | 1 | 6.2 | 2.65 | 1.29 | 3.2 | 5.89 | 9.71 | 64.8 | 21.3 | 10.1 |
| C1365 | 2 | 41 | 2.83 ± 0.004 | 0 | 0 | 1 | 3.28 | 1.61 | 1.4 | 1.17 | 6.02 | 7.79 | 56.6 | 36.1 | 5.5 |
| C1367 | 1 | 57 | 4.54 ± 0.065 | 1 | 0 | 1 | 3.53 | 0.53 | 1.15 | 1.91 | 19.2 | 8.32 | 62.1 | 26.1 | 7.8 |
| C1370 | 2 | 60 | 4.02 ± 0.012 | 1 | 1 | 0 | 3.21 | 1.94 | 0.62 | 1.69 | 6.72 | 8.89 | 67.0 | 23.7 | 5.5 |
| C1371 | 2 | 72 | 4.03 ± 0.202 | 0 | 0 | 0 | 4.18 | 1.18 | 1.03 | 2.54 | 5.51 | 6.62 | 46.1 | 34.6 | 10.9 |
| C1372 | 2 | 47 | 3.86 ± 0.004 | 1 | 0 | 0 | 3.96 | 0.95 | 1.15 | 2.1 | 5.17 | 5.35 | 53.8 | 32.0 | 10.1 |
| C1373 | 1 | 65 | 3.95 ± 0.182 | 1 | 0 | 0 | 4.63 | 1.54 | 1.18 | 2.82 | 5.6 | 8.72 | 63.9 | 20.3 | 8.6 |
| C1376 | 1 | 71 | 4.16 ± 0.037 | 1 | 0 | 1 | 4.97 | 1.21 | 1.21 | 3.07 | 7.46 | 19.38 | 90.0 | 6.0 | 3.9 |
| C1377 | 1 | 41 | 4.23 ± 0.075 | 0 | 1 | 0 | 4.06 | 4.55 | 0.49 | 1.69 | 9.8 | 10.52 | 69.0 | 20.7 | 4.7 |
| C1383 | 2 | 45 | 4.18 ± 0.066 | 1 | 0 | 0 | 5.61 | 2.5 | 1.1 | 3.24 | 5.91 | 8.83 | 58.2 | 31.6 | 7.5 |
| C1386 | 1 | 59 | 4.15 ± 0.105 | 1 | 0 | 0 | 3.67 | 1.21 | 0.89 | 2.09 | 5.52 | 6.16 | 58.1 | 30.8 | 3.9 |
| C1388 | 1 | 62 | 3.56 ± 0.015 | 1 | 0 | 0 | 3.48 | 3.65 | 0.83 | 1.36 | 6.83 | 4.28 | 41.4 | 50.2 | 8.4 |
| ^a^ 1 stands for Male, 2 stands for Female.  ^b^ most of the CAD patients with underlying disease (hypertension, hyperlipidemia, and Type 2 diabetes) were under proper therapy, the status with ( represented by 1) or without (represented by 0) specific medical conditions rather than unstable clinical examination results were recorded in this research.  TC, total cholesterol; TG, total triglyceride; HDL-C, high density lipoprotein cholesterol; LDL-C, low-density lipoprotein cholesterol; FBG, fasting blood glucose; CBC, complete blood count; PBL, peripheral blood leukocyte; NEU, neutrophil; LYM, lymphocyte; MONO, monocyte | | | | | | | | | | | | | | | |

**Table S3.** Clinical characteristics of 50 healthy controls in the randomly selected subgroup.

| Sample ID | Gender^a^ | Age | HT^b^ | HL^b^ | DM^b^ | TC | TG | HLL-c | LDL-c | FBG | SBP | DBP | CBC | | | |
| --- | --- | --- | --- | --- | --- | --- | --- | --- | --- | --- | --- | --- | --- | --- | --- | --- |
|  |  |  |  |  |  | (mmol/L) | | | | | (mm Hg) | | PBL(10^9^/L) | NEU% | LYM% | MONO% |
| N130 | 2 | 72 | 1 | 0 | 0 | 4.61 | 0.49 | 1.27 | 2.61 | 4.55 | 126 | 88 | 3.51 | 60.7 | 32 | 5.4 |
| N132 | 1 | 80 | 0 | 0 | 0 | 3.25 | 1.56 | 1.95 | 2.54 | 5.05 | 124 | 85 | 8.3 | 86.4 | 8.6 | 4.8 |
| N144 | 1 | 54 | 0 | 1 | 0 | 5.17 | 1.21 | 1.32 | 2.94 | 6.01 | 108 | 74 | 8.71 | 65.1 | 24.3 | 7.6 |
| N151 | 1 | 53 | 0 | 0 | 0 | 5.03 | 1.04 | 1.48 | 3.03 | 5.32 | 99 | 63 | 6.76 | 42.7 | 43.3 | 10.2 |
| N172 | 2 | 55 | 1 | 1 | 1 | 5.1 | 0.9 | 1.07 | 3.07 | 4.63 | 117 | 85 | 5.75 | 65.6 | 25.6 | 5.8 |
| N196 | 1 | 74 | 0 | 0 | 0 | 4.79 | 0.98 | 1.27 | 2.82 | 5.02 | 139 | 106 | 7.12 | 77.1 | 18.6 | 3 |
| N205 | 2 | 78 | 0 | 0 | 0 | 3.81 | 1.67 | 1.56 | 2.31 | 5.15 | 138 | 104 | 6.47 | 60.5 | 29.5 | 8 |
| N324 | 2 | 75 | 1 | 0 | 0 | 2.95 | 1.06 | 0.99 | 1.78 | 5.14 | 114 | 83 | 5.27 | 53.6 | 36.6 | 5.6 |
| N372 | 1 | 68 | 0 | 0 | 0 | 4.23 | 0.64 | 1.37 | 0.94 | 5.67 | 131 | 97 | 4.43 | 65.1 | 25.7 | 6.6 |
| N397 | 1 | 49 | 1 | 1 | 1 | 3.82 | 1.2 | 1 | 2.89 | 5.39 | 131 | 100 | 4.56 | 64.7 | 23.7 | 9 |
| N402 | 1 | 57 | 0 | 0 | 0 | 4.77 | 1.63 | 1.05 | 2.16 | 6.07 | 115 | 80 | 7.98 | 56.6 | 32.2 | 7 |
| N411 | 2 | 65 | 1 | 0 | 1 | 4.2 | 1.12 | 1.23 | 2.27 | 5.63 | 113 | 76 | 4.44 | 58.7 | 31.2 | 5.2 |
| N412 | 2 | 68 | 0 | 0 | 0 | 4.29 | 0.4 | 1.85 | 1.47 | 4.84 | 110 | 74 | 5.14 | 44.8 | 42.5 | 9.8 |
| N415 | 1 | 52 | 0 | 0 | 0 | 4.3 | 1.4 | 1.72 | 2.98 | 4.61 | 112 | 74 | 5.95 | 66.6 | 23.2 | 7.3 |
| N422 | 1 | 63 | 1 | 1 | 0 | 4.29 | 1.68 | 1.53 | 2.36 | 4.96 | 129 | 94 | 6.3 | 69.4 | 22.4 | 6.3 |
| N423 | 2 | 53 | 0 | 0 | 0 | 4.97 | 1.55 | 1.27 | 2.37 | 5.13 | 120 | 82 | 7.52 | 49 | 39.8 | 9.9 |
| N423 | 2 | 68 | 1 | 0 | 0 | 4.51 | 1.52 | 0.89 | 2.61 | 5.47 | 92 | 62 | 5.46 | 57 | 33.2 | 7.5 |
| N431 | 2 | 75 | 0 | 0 | 0 | 5.11 | 1.34 | 1.48 | 2.96 | 5.37 | 128 | 96 | 5.39 | 63.8 | 26.6 | 5.5 |
| N452 | 2 | 57 | 1 | 0 | 0 | 4.01 | 0.6 | 1.75 | 2.83 | 4.76 | 125 | 91 | 5.34 | 63.8 | 26.5 | 8.6 |
| N453 | 1 | 81 | 0 | 0 | 0 | 5.12 | 0.69 | 1.02 | 2.9 | 4.69 | 130 | 95 | 5.66 | 39.3 | 40.8 | 17.6 |
| N460 | 2 | 68 | 0 | 0 | 0 | 4.13 | 1.5 | 1.81 | 2.81 | 5.45 | 137 | 100 | 8.83 | 64.2 | 27 | 7.4 |
| N478 | 1 | 64 | 0 | 0 | 0 | 3.9 | 1.19 | 1.52 | 2.32 | 3.31 | 139 | 106 | 5.25 | 58 | 33.5 | 7 |
| N520 | 2 | 59 | 0 | 0 | 0 | 4.14 | 0.7 | 2.28 | 2.91 | 4.94 | 117 | 77 | 6.39 | 67.1 | 28.2 | 3.4 |
| N521 | 2 | 63 | 0 | 0 | 0 | 4.24 | 1.62 | 1.95 | 2.43 | 5.76 | 109 | 70 | 5.07 | 62.5 | 29.8 | 6.1 |
| N536 | 1 | 59 | 1 | 0 | 0 | 4.62 | 1.55 | 1.18 | 2.84 | 5.32 | 93 | 59 | 5.52 | 54.4 | 35.8 | 7.1 |
| N541 | 2 | 70 | 0 | 0 | 1 | 4.45 | 1.55 | 1.31 | 2.37 | 4.66 | 90 | 51 | 6.25 | 67.7 | 24.6 | 5.3 |
| N546 | 1 | 74 | 0 | 0 | 0 | 3.81 | 1.68 | 1.61 | 1.42 | 4.87 | 96 | 61 | 6.38 | 51.9 | 32.5 | 11.6 |
| N556 | 1 | 56 | 1 | 0 | 1 | 4.5 | 0.45 | 1.27 | 2.17 | 5.31 | 111 | 74 | 4.36 | 60.8 | 19.3 | 13.3 |
| N559 | 1 | 54 | 0 | 0 | 0 | 2.84 | 0.59 | 1.2 | 1.81 | 4.15 | 123 | 89 | 4.88 | 61.9 | 24.8 | 9.2 |
| N581 | 2 | 53 | 0 | 1 | 0 | 5.13 | 1.13 | 1.54 | 3.26 | 5.42 | 94 | 62 | 6.52 | 38.6 | 44.6 | 11.9 |
| N582 | 2 | 57 | 1 | 1 | 1 | 5.28 | 1.65 | 1.21 | 2.96 | 5.36 | 114 | 80 | 4.31 | 41.1 | 46.2 | 7.5 |
| N593 | 1 | 47 | 0 | 1 | 1 | 3.03 | 1.72 | 1.46 | 3 | 5.39 | 117 | 78 | 5.43 | 87.3 | 10.3 | 2.2 |
| N600 | 2 | 79 | 0 | 0 | 0 | 3.3 | 1.67 | 1.83 | 1.62 | 6.04 | 95 | 56 | 9.9 | 57.7 | 33.9 | 6.5 |
| N624 | 2 | 64 | 0 | 0 | 0 | 4.41 | 0.88 | 1.55 | 1.43 | 5.27 | 137 | 105 | 3.4 | 58.6 | 28 | 10.6 |
| N664 | 2 | 80 | 1 | 1 | 1 | 3.42 | 1.74 | 1.37 | 1.91 | 6.11 | 113 | 83 | 4.3 | 47.1 | 34.4 | 12 |
| N668 | 1 | 68 | 0 | 0 | 0 | 4.02 | 1.36 | 0.98 | 3.1 | 4.77 | 112 | 79 | 5.49 | 44.9 | 48.2 | 5.9 |
| N728 | 2 | 57 | 0 | 0 | 0 | 4.59 | 0.97 | 1.56 | 2.43 | 5.38 | 104 | 64 | 4.48 | 51 | 41 | 6.1 |
| N746 | 1 | 59 | 0 | 0 | 0 | 5.04 | 1.57 | 1.46 | 3.05 | 5.42 | 126 | 88 | 6.72 | 64.2 | 25 | 7.1 |
| N748 | 1 | 68 | 1 | 0 | 0 | 3.95 | 1.01 | 1.14 | 2.77 | 5 | 137 | 101 | 5.36 | 60.4 | 24.9 | 12.1 |
| N751 | 2 | 80 | 0 | 0 | 0 | 3.66 | 1.42 | 1.64 | 2.34 | 5.19 | 121 | 89 | 4.21 | 51.1 | 34.9 | 8.1 |
| N752 | 2 | 67 | 0 | 0 | 0 | 5.07 | 1.64 | 1.55 | 2.64 | 5.24 | 126 | 91 | 6.81 | 53 | 35 | 10.1 |
| N765 | 2 | 76 | 0 | 0 | 1 | 4.84 | 0.67 | 1.04 | 2.45 | 4.71 | 105 | 65 | 2.45 | 58.1 | 30.7 | 7.2 |
| N781 | 1 | 60 | 1 | 0 | 0 | 3.83 | 1.2 | 1.82 | 2.12 | 4.9 | 93 | 57 | 5.67 | 51.7 | 36.8 | 6.9 |
| N796 | 1 | 72 | 0 | 0 | 0 | 3.76 | 0.76 | 1.62 | 1.55 | 5.47 | 139 | 108 | 5.25 | 61.3 | 28.6 | 7.8 |
| N801 | 2 | 63 | 1 | 0 | 0 | 3.09 | 0.71 | 1.38 | 1.51 | 4.6 | 113 | 75 | 12.22 | 71.3 | 21.4 | 6.8 |
| N820 | 1 | 66 | 0 | 1 | 0 | 4.87 | 1.62 | 1.2 | 2.61 | 4.86 | 102 | 68 | 6.63 | 62.2 | 30.5 | 6.4 |
| N825 | 1 | 57 | 0 | 0 | 1 | 3.71 | 1.31 | 1.21 | 1.56 | 7.66 | 132 | 101 | 8.74 | 77 | 18.3 | 4.2 |
| N851 | 1 | 48 | 0 | 0 | 0 | 3.1 | 1.15 | 1.35 | 2.6 | 5.04 | 102 | 70 | 4.53 | 47.6 | 41 | 9.1 |
| N858 | 1 | 62 | 1 | 0 | 0 | 4.87 | 1.19 | 1.28 | 2.56 | 5.38 | 123 | 84 | 4.77 | 59.7 | 30.2 | 7.4 |
| N880 | 1 | 53 | 0 | 0 | 0 | 4.73 | 1.51 | 1.15 | 2.83 | 4.12 | 103 | 64 | 4.54 | 58.1 | 28.5 | 9.2 |
| ^a^ 1 stands for Male, 2 stands for Female.  ^b^ The status with ( represented by 1) or without (represented by 0) specific medical conditions rather than unstable clinical examination results were recorded in this research.  TC, total cholesterol; TG, total triglyceride; HDL-C, high density lipoprotein cholesterol; LDL-C, low-density lipoprotein cholesterol; FBG, fasting blood glucose; SBP, [systolic](javascript:void(0);) blood [pressure](javascript:void(0);); DBP, diastolic blood pressure; CBC, complete blood count; PBL, peripheral blood leukocyte; NEU, neutrophil; LYM, lymphocyte; MONO, monocyte. | | | | | | | | | | | | | | | | |

**Table S4.** Clinical characteristics of 50 CAD patients in the randomly selected subgroup.

| Sample ID | Gender^a^ | Age | HT^b^ | HL^b^ | DM^b^ | TC | TG | HLL-c | LDL-c | FBG | CBC | | | |
| --- | --- | --- | --- | --- | --- | --- | --- | --- | --- | --- | --- | --- | --- | --- |
|  |  |  |  |  |  | (mmol/L) | | | | | PBL(10^9^/L) | NEU% | LYM% | MONO% |
| C271 | 2 | 64 | 0 | 1 | 0 | 5.12 | 1.83 | 1.2 | 4.05 | 6.11 | 7.19 | 56.6 | 32.1 | 8 |
| C272 | 1 | 72 | 0 | 1 | 1 | 3.92 | 0.68 | 2.01 | 1.81 | 6.83 | 5.33 | 65.2 | 22.4 | 10.3 |
| C278 | 1 | 77 | 1 | 1 | 0 | 4.23 | 1.41 | 1.22 | 2.81 | 5.84 | 6.39 | 66.9 | 24.3 | 7.4 |
| C282 | 1 | 41 | 1 | 1 | 0 | 3.95 | 5.99 | 0.96 | 1.63 | 4.74 | 9.18 | 57.8 | 29.7 | 7.8 |
| C293 | 1 | 47 | 0 | 1 | 0 | 6.34 | 3.07 | 1.53 | 3.98 | 5.85 | 9.57 | 85.4 | 9.4 | 4.8 |
| C299 | 1 | 61 | 1 | 0 | 1 | 3.8 | 1.46 | 1.37 | 2.04 | 9.93 | 6.8 | 55.3 | 31.1 | 10.9 |
| C310 | 1 | 73 | 1 | 0 | 0 | 3.6 | 1.23 | 1.11 | 2.12 | 5.09 | 4.6 | 64.3 | 24.7 | 7.5 |
| C312 | 1 | 66 | 0 | 0 | 1 | 4.69 | 1.33 | 1.19 | 3.13 | 7.49 | 6.53 | 60.2 | 29.9 | 7.52 |
| C335 | 2 | 63 | 0 | 0 | 0 | 4.34 | 1.1 | 1.62 | 2.38 | 5.44 | 11.68 | 82.5 | 8.8 | 8.6 |
| C361 | 2 | 66 | 1 | 0 | 1 | 5.05 | 0.97 | 1.31 | 3.52 | 7.12 | 12.48 | 81 | 12.6 | 5.9 |
| C364 | 1 | 63 | 1 | 1 | 1 | 5.62 | 3.1 | 1.04 | 3.45 | 6.31 | 6.89 | 63.3 | 24.4 | 8.5 |
| C372 | 2 | 64 | 1 | 1 | 0 | 6.18 | 1.35 | 1.11 | 4.16 | 4.98 | 5.34 | 72 | 20.5 | 6.3 |
| C386 | 1 | 79 | 0 | 0 | 0 | 4.17 | 0.5 | 1.46 | 1.69 | 5.26 | 4.11 | 64.6 | 21.3 | 12.2 |
| C401 | 1 | 48 | 1 | 0 | 0 | 5.01 | 1.44 | 1.43 | 1.75 | 6.15 | 6.26 | 62.5 | 27.3 | 8 |
| C414 | 1 | 58 | 0 | 0 | 0 | 4.65 | 1.08 | 1.31 | 3.12 | 5.21 | 12.33 | 73.3 | 15.3 | 10.7 |
| C447 | 1 | 55 | 1 | 1 | 1 | 4.05 | 2.06 | 1 | 2.41 | 9.95 | 11.09 | 87.8 | 9.5 | 2.2 |
| C457 | 2 | 71 | 0 | 0 | 1 | 4.51 | 1.23 | 1.5 | 1.53 | 4.59 | 11.7 | 64.8 | 29.5 | 4.8 |
| C482 | 2 | 64 | 1 | 0 | 0 | 3.84 | 1.1 | 1.26 | 2.05 | 5.87 | 6.6 | 57.6 | 29.2 | 7 |
| C494 | 2 | 79 | 1 | 0 | 0 | 3.59 | 1.34 | 1.08 | 2.11 | 5.26 | 6.03 | 70.4 | 17.2 | 10.2 |
| C504 | 1 | 78 | 1 | 0 | 1 | 3.22 | 0.98 | 1.35 | 2.34 | 6.05 | 6.23 | 63.4 | 23 | 11 |
| C540 | 1 | 63 | 1 | 0 | 0 | 4.86 | 1.47 | 1.05 | 3.02 | 5.12 | 5.32 | 55.6 | 32.5 | 8.1 |
| C557 | 1 | 76 | 1 | 0 | 0 | 4.44 | 1.3 | 1.04 | 2.67 | 5.31 | 6.66 | 65 | 21.3 | 10.2 |
| C572 | 1 | 64 | 1 | 0 | 0 | 3.15 | 0.92 | 1.23 | 1.52 | 5.18 | 7.09 | 55.7 | 34.4 | 7.9 |
| C583 | 2 | 61 | 0 | 1 | 1 | 5.3 | 3.4 | 1.02 | 3.22 | 4.94 | 6.98 | 57.3 | 34.5 | 6.5 |
| C587 | 2 | 64 | 1 | 1 | 1 | 4.88 | 2.53 | 0.94 | 2.84 | 7.08 | 9.02 | 68.9 | 20.8 | 7.4 |
| C589 | 1 | 59 | 1 | 0 | 0 | 3.46 | 1.22 | 0.73 | 2.57 | 5.37 | 5.02 | 61.2 | 26.8 | 8.8 |
| C658 | 2 | 57 | 0 | 0 | 0 | 3.99 | 0.52 | 2.58 | 2.58 | 5.11 | 6.56 | 61.6 | 31.1 | 5 |
| C733 | 1 | 44 | 1 | 0 | 1 | 5.08 | 4 | 1.03 | 3.2 | 8.76 | 4.61 | 55.3 | 32.1 | 8.2 |
| C743 | 2 | 65 | 0 | 0 | 1 | 3.18 | 1.03 | 1.61 | 1.87 | 5.2 | 7.42 | 39.3 | 50.8 | 7.2 |
| C757 | 2 | 63 | 1 | 1 | 1 | 5.59 | 1.95 | 1.06 | 4.39 | 7.75 | 6.86 | 64.4 | 22.9 | 10 |
| C771 | 1 | 69 | 1 | 0 | 1 | 3.85 | 0.79 | 1.03 | 2.96 | 7.12 | 15.15 | 96 | 3.2 | 0.7 |
| C826 | 1 | 56 | 1 | 0 | 0 | 4.39 | 1.52 | 1.08 | 2.32 | 4.42 | 5.75 | 61.4 | 31.2 | 5.5 |
| C856 | 1 | 48 | 1 | 0 | 0 | 3.25 | 3.4 | 0.54 | 1.55 | 5.88 | 5.82 | 64.2 | 25 | 6.6 |
| C939 | 1 | 77 | 1 | 0 | 0 | 4.74 | 1.67 | 1.02 | 3.03 | 5.62 | 4.58 | 65.8 | 19.5 | 12.5 |
| C981 | 2 | 74 | 0 | 1 | 0 | 4.67 | 1.85 | 1.46 | 2.92 | 5.43 | 7.13 | 73.8 | 20.2 | 5.7 |
| C990 | 2 | 61 | 1 | 0 | 0 | 5.05 | 1.29 | 1.54 | 2.89 | 6.69 | 15.01 | 78 | 15.7 | 5.7 |
| C993 | 2 | 43 | 1 | 0 | 1 | 4.82 | 1.91 | 1.11 | 2.95 | 8.06 | 6.38 | 51.9 | 32.5 | 11.6 |
| C997 | 1 | 55 | 0 | 1 | 0 | 6.86 | 2.59 | 0.88 | 4.9 | 5.57 | 6.5 | 61 | 27 | 8.4 |
| C1024 | 1 | 65 | 1 | 0 | 0 | 4.75 | 0.42 | 1.34 | 2.89 | 4.08 | 5.18 | 54.2 | 29.4 | 8.2 |
| C1099 | 2 | 59 | 0 | 1 | 0 | 7.67 | 2.24 | 0.91 | 5.68 | 5.68 | 15.26 | 82.9 | 10.5 | 6.2 |
| C1114 | 1 | 47 | 1 | 0 | 1 | 3.77 | 1.12 | 0.71 | 2.63 | 7.6 | 23 | 94 | 2.5 | 3.5 |
| C1138 | 2 | 52 | 1 | 0 | 0 | 3.74 | 1.04 | 0.82 | 1.3 | 5.05 | 4.46 | 60.6 | 32.4 | 5.3 |
| C1153 | 2 | 46 | 0 | 1 | 1 | 4.89 | 2.36 | 0.96 | 2.17 | 5.61 | 4.51 | 52.8 | 38.8 | 6.8 |
| C1189 | 2 | 68 | 0 | 0 | 1 | 2.89 | 1.75 | 1.16 | 1.38 | 8.74 | 5.72 | 65.5 | 23.6 | 9.2 |
| C1233 | 2 | 60 | 1 | 0 | 1 | 3.47 | 1.61 | 1.61 | 1.7 | 6.24 | 5.88 | 64.1 | 27.5 | 6 |
| C1257 | 1 | 70 | 0 | 1 | 1 | 5.55 | 2.08 | 1.03 | 3.78 | 5.76 | 9.68 | 60.4 | 28.2 | 6.2 |
| C1337 | 2 | 51 | 1 | 0 | 1 | 5.05 | 1.4 | 1.3 | 2.64 | 5.89 | 16.3 | 86.1 | 3.8 | 3 |
| C1338 | 2 | 56 | 0 | 0 | 0 | 4.68 | 0.74 | 2.24 | 2.95 | 5.9 | 7.64 | 72.4 | 17.3 | 8.5 |
| C1341 | 2 | 60 | 0 | 0 | 1 | 2.85 | 1.42 | 1.34 | 1.41 | 9.87 | 7.66 | 60.6 | 31.1 | 6.5 |
| C1381 | 1 | 65 | 1 | 1 | 1 | 4.97 | 2.13 | 1.12 | 2.94 | 7.73 | 4.38 | 55.4 | 35.2 | 8.3 |
| ^a^ 1 stands for Male, 2 stands for Female.  ^b^ most of the CAD patients with underlying disease (hypertension, hyperlipidemia, and Type 2 diabetes) were under proper therapy, the status with ( represented by 1) or without (represented by 0) specific medical conditions rather than unstable clinical examination results were recorded in this research.  TC, total cholesterol; TG, total triglyceride; HDL-C, high density lipoprotein cholesterol; LDL-C, low-density lipoprotein cholesterol; FBG, fasting blood glucose; CBC, complete blood count; PBL, peripheral blood leukocyte; NEU, neutrophil; LYM, lymphocyte; MONO, monocyte. | | | | | | | | | | | | | | |

**Table S5.** Measured contents of 5-mdC and 5-hmdC in genomic DNA of blood samples in subgroup with different blood cell sources.

| Sample ID | 5-mC, % | | | | 5-hmC, % | | | | DNMT1 expression |
| --- | --- | --- | --- | --- | --- | --- | --- | --- | --- |
|  | PBL | NEU | LYM | MONO | PBL | NEU | LYM | MONO |  |
| N130 | 4.04 ± 0.156 | 3.54 ± 0.393 | 3.74 ± 0.294 | 3.86 ± 0.139 | 0.0163 ± 0.00309 | 0.0182 ± 0.00084 | 0.0103 ± 0.00070 | 0.0099 ± 0.00052 | 0.0121 |
| N132 | 5.19 ± 0.052 | 4.87 ± 0.371 | 3.84 ± 0.163 | 3.34 ± 0.048 | 0.0157 ± 0.00047 | 0.0155 ± 0.00122 | 0.0106 ± 0.00175 | 0.0100 ± 0.00141 | 0.0074 |
| N144 | 4.38 ± 0.052 | 5.22 ± 0.047 | 3.83 ± 0.180 | 3.63 ± 0.093 | 0.0159 ± 0.00141 | 0.0183 ± 0.00061 | 0.0229 ± 0.00100 | 0.0159 ± 0.00105 | 0.0136 |
| N151 | 4.65 ± 0.130 | 6.09 ± 0.073 | 3.18 ± 0.012 | 3.08 ± 0.176 | 0.0198 ± 0.00139 | 0.0161 ± 0.00133 | 0.0169 ± 0.00050 | 0.0246 ± 0.00165 | 0.0234 |
| N172 | 3.67 ± 0.396 | 4.76 ± 0.063 | 3.01 ± 0.140 | 3.21 ± 0.086 | 0.0133 ± 0.00053 | 0.0187 ± 0.00033 | 0.0152 ± 0.00180 | 0.0190 ± 0.00035 | 0.0065 |
| N196 | 4.29 ± 0.049 | 4.44 ± 0.238 | 3.52 ± 0.080 | 3.14 ± 0.136 | 0.0192 ± 0.00019 | 0.0209 ± 0.00067 | 0.0113 ± 0.00144 | 0.0156 ± 0.00184 | 0.0064 |
| N205 | 4.48 ± 0.001 | 4.19 ± 0.012 | 3.17 ± 0.288 | 3.64 ± 0.194 | 0.0187 ± 0.00175 | 0.0256 ± 0.00032 | 0.0291 ± 0.00191 | 0.0114 ± 0.00043 | 0.0041 |
| N324 | 4.04 ± 0.073 | 5.46 ± 0.166 | 3.47 ± 0.332 | 3.41 ± 0.054 | 0.0209 ± 0.00129 | 0.0180 ± 0.00190 | 0.0132 ± 0.00197 | 0.0172 ± 0.00108 | 0.0083 |
| N372 | 4.25 ± 0.129 | 4.23 ± 0.033 | 3.02 ± 0.141 | 3.64 ± 0.002 | 0.0126 ± 0.00175 | 0.0193 ± 0.00199 | 0.0256 ± 0.00508 | 0.0214 ± 0.00083 | 0.0104 |
| N397 | 3.55 ± 0.304 | 4.05 ± 0.052 | 3.13 ± 0.081 | 3.26 ± 0.031 | 0.0197 ± 0.00158 | 0.0185 ± 0.00045 | 0.0120 ± 0.00194 | 0.0107 ± 0.00139 | 0.0104 |
| N402 | 4.32 ± 0.151 | 4.93 ± 0.084 | 2.54 ± 0.148 | 3.14 ± 0.032 | 0.0170 ± 0.00185 | 0.0159 ± 0.00152 | 0.0141 ± 0.00051 | 0.0190 ± 0.00115 | 0.0118 |
| N411 | 3.68 ± 0.138 | 4.23 ± 0.082 | 3.08 ± 0.023 | 3.23 ± 0.030 | 0.0179 ± 0.00057 | 0.0195 ± 0.00066 | 0.0260 ± 0.00182 | 0.0184 ± 0.00307 | 0.0049 |
| N412 | 3.30 ± 0.101 | 3.75 ± 0.133 | 2.99 ± 0.179 | 3.58 ± 0.063 | 0.0199 ± 0.00034 | 0.0146 ± 0.00083 | 0.0110 ± 0.00071 | 0.0133 ± 0.00091 | 0.0038 |
| N415 | 3.47 ± 0.084 | 3.79 ± 0.118 | 3.35 ± 0.137 | 3.64 ± 0.047 | 0.0221 ± 0.00108 | 0.0139 ± 0.00121 | 0.0215 ± 0.00059 | 0.0221 ± 0.00030 | 0.0102 |
| N422 | 3.94 ± 0.082 | 4.65 ± 0.189 | 3.37 ± 0.066 | 3.51 ± 0.101 | 0.0146 ± 0.00004 | 0.0233 ± 0.00170 | 0.0247 ± 0.00175 | 0.0191 ± 0.00190 | 0.007 |
| N423 | 3.72 ± 0.108 | 4.48 ± 0.164 | 3.13 ± 0.127 | 3.28 ± 0.002 | 0.0167 ± 0.00023 | 0.0225 ± 0.00096 | 0.0099 ± 0.00092 | 0.0162 ± 0.00079 | 0.0087 |
| N423 | 3.57 ± 0.190 | 4.66 ± 0.191 | 3.16 ± 0.084 | 3.19 ± 0.154 | 0.0154 ± 0.00238 | 0.0211 ± 0.00032 | 0.0116 ± 0.00170 | 0.0098 ± 0.00148 | 0.0095 |
| N431 | 3.91 ± 0.110 | 4.42 ± 0.299 | 3.50 ± 0.027 | 3.86 ± 0.145 | 0.0124 ± 0.00167 | 0.0196 ± 0.00164 | 0.0132 ± 0.00043 | 0.0192 ± 0.00074 | 0.0092 |
| N452 | 4.09 ± 0.178 | 4.77 ± 0.091 | 3.33 ± 0.185 | 2.35 ± 0.072 | 0.0091 ± 0.00194 | 0.0190 ± 0.00196 | 0.0172 ± 0.00087 | 0.0186 ± 0.00063 | 0.0011 |
| N453 | 3.00 ± 0.155 | 4.76 ± 0.058 | 3.08 ± 0.007 | 2.96 ± 0.151 | 0.0136 ± 0.00062 | 0.0220 ± 0.00036 | 0.0156 ± 0.00085 | 0.0123 ± 0.00157 | 0.003 |
| N460 | 4.78 ± 0.181 | 5.21 ± 0.108 | 3.70 ± 0.037 | 3.16 ± 0.075 | 0.0131 ± 0.00153 | 0.0198 ± 0.00071 | 0.0129 ± 0.00029 | 0.0214 ± 0.00151 | 0.0129 |
| N478 | 3.49 ± 0.196 | 4.03 ± 0.032 | 2.87 ± 0.036 | 3.29 ± 0.124 | 0.0142 ± 0.00217 | 0.0162 ± 0.00196 | 0.0173 ± 0.00064 | 0.0166 ± 0.00057 | 0.0070 |
| N520 | 4.73 ± 0.097 | 6.57 ± 0.056 | 2.13 ± 0.097 | 3.22 ± 0.118 | 0.0143 ± 0.00195 | 0.0143 ± 0.00087 | 0.0142 ± 0.00175 | 0.0116 ± 0.00123 | 0.0098 |
| N521 | 4.32 ± 0.030 | 4.79 ± 0.112 | 3.70 ± 0.066 | 3.25 ± 0.092 | 0.0173 ± 0.00100 | 0.0157 ± 0.00023 | 0.0090 ± 0.00019 | 0.0234 ± 0.00141 | 0.0052 |
| N536 | 4.68 ± 0.093 | 4.09 ± 0.169 | 3.32 ± 0.091 | 3.75 ± 0.181 | 0.0107 ± 0.00137 | 0.0259 ± 0.00221 | 0.0141 ± 0.00060 | 0.0116 ± 0.00162 | 0.0086 |
| N541 | 4.34 ± 0.050 | 4.68 ± 0.001 | 3.17 ± 0.134 | 4.06 ± 0.051 | 0.0120 ± 0.00108 | 0.0194 ± 0.00042 | 0.0200 ± 0.00002 | 0.0148 ± 0.00186 | 0.0117 |
| N546 | 3.88 ± 0.393 | 4.24 ± 0.092 | 3.50 ± 0.118 | 3.27 ± 0.081 | 0.0155 ± 0.00249 | 0.0200 ± 0.00025 | 0.0096 ± 0.00198 | 0.0101 ± 0.00048 | 0.0071 |
| N556 | 3.18 ± 0.144 | 4.13 ± 0.222 | 3.16 ± 0.180 | 3.38 ± 0.058 | 0.0063 ± 0.00059 | 0.0183 ± 0.00121 | 0.0013 ± 0.00038 | 0.0112 ± 0.00324 | 0.0086 |
| N559 | 3.68 ± 0.143 | 4.10 ± 0.006 | 3.65 ± 0.170 | 3.73 ± 0.215 | 0.0218 ± 0.00068 | 0.0185 ± 0.00180 | 0.0227 ± 0.00096 | 0.0117 ± 0.00028 | 0.0066 |
| N581 | 2.12 ± 0.018 | 3.24 ± 0.131 | 3.59 ± 0.164 | 3.15 ± 0.165 | 0.0173 ± 0.00150 | 0.0118 ± 0.00130 | 0.0136 ± 0.00178 | 0.0094 ± 0.00056 | 0.0046 |
| N582 | 2.67 ± 0.010 | 4.05 ± 0.098 | 2.63 ± 0.106 | 2.96 ± 0.108 | 0.0068 ± 0.00099 | 0.0091 ± 0.00150 | 0.0040 ± 0.00162 | 0.0061 ± 0.00133 | 0.0090 |
| N593 | 3.38 ± 0.018 | 4.88 ± 0.146 | 2.61 ± 0.138 | 2.94 ± 0.170 | 0.0051 ± 0.00001 | 0.0125 ± 0.00045 | 0.0212 ± 0.00120 | 0.0033 ± 0.00179 | 0.0083 |
| N600 | 5.45 ± 0.054 | 3.77 ± 0.124 | 3.88 ± 0.021 | 3.31 ± 0.280 | 0.0197 ± 0.00138 | 0.0212 ± 0.00207 | 0.0222 ± 0.00013 | 0.0087 ± 0.00152 | 0.0057 |
| N624 | 4.13 ± 0.053 | 4.69 ± 0.106 | 3.50 ± 0.073 | 3.23 ± 0.070 | 0.0059 ± 0.00035 | 0.0173 ± 0.00130 | 0.0109 ± 0.00121 | 0.0041 ± 0.00006 | 0.0033 |
| N664 | 6.05 ± 0.195 | 5.26 ± 0.160 | 2.84 ± 0.063 | 3.90 ± 0.241 | 0.0052 ± 0.00109 | 0.0168 ± 0.00099 | 0.0225 ± 0.00165 | 0.0206 ± 0.00164 | 0.0096 |
| N668 | 3.49 ± 0.028 | 4.27 ± 0.117 | 2.85 ± 0.166 | 3.19 ± 0.122 | 0.0119 ± 0.00091 | 0.0245 ± 0.00141 | 0.0181 ± 0.00150 | 0.0189 ± 0.00053 | 0.0062 |
| N728 | 4.13 ± 0.023 | 5.11 ± 0.155 | 3.25 ± 0.080 | 3.59 ± 0.068 | 0.0222 ± 0.00062 | 0.0246 ± 0.00076 | 0.0234 ± 0.00177 | 0.0119 ± 0.00349 | 0.0118 |
| N746 | 4.06 ± 0.197 | 2.30 ± 0.021 | 2.98 ± 0.126 | 3.73 ± 0.041 | 0.0225 ± 0.00064 | 0.0212 ± 0.00122 | 0.0172 ± 0.00134 | 0.0138 ± 0.00098 | 0.0062 |
| N748 | 4.36 ± 0.090 | 4.88 ± 0.084 | 4.05 ± 0.081 | 3.31 ± 0.006 | 0.0249 ± 0.00147 | 0.0224 ± 0.00060 | 0.0209 ± 0.00021 | 0.0201 ± 0.00133 | 0.0035 |
| N751 | 4.09 ± 0.104 | 4.47 ± 0.112 | 3.04 ± 0.184 | 3.37 ± 0.396 | 0.0221 ± 0.00409 | 0.0174 ± 0.00173 | 0.0241 ± 0.00165 | 0.0136 ± 0.00102 | 0.0076 |
| N752 | 4.19 ± 0.104 | 4.24 ± 0.155 | 3.25 ± 0.073 | 3.41 ± 0.110 | 0.0198 ± 0.00173 | 0.0220 ± 0.00011 | 0.0126 ± 0.00128 | 0.0177 ± 0.00031 | 0.0063 |
| N765 | 3.12 ± 0.195 | 3.96 ± 0.256 | 2.76 ± 0.116 | 3.48 ± 0.081 | 0.0113 ± 0.00077 | 0.0126 ± 0.00047 | 0.0261 ± 0.00119 | 0.0145 ± 0.00038 | 0.0038 |
| N781 | 4.39 ± 0.146 | 4.73 ± 0.200 | 3.17 ± 0.013 | 3.72 ± 0.251 | 0.0166 ± 0.00055 | 0.0229 ± 0.00037 | 0.0206 ± 0.00135 | 0.0102 ± 0.00020 | 0.0080 |
| N796 | 4.20 ± 0.039 | 4.13 ± 0.180 | 3.46 ± 0.182 | 3.94 ± 0.021 | 0.0211 ± 0.00191 | 0.0132 ± 0.00180 | 0.0108 ± 0.00196 | 0.0146 ± 0.00085 | 0.0092 |
| N801 | 5.16 ± 0.045 | 5.01 ± 0.244 | 3.76 ± 0.083 | 4.14 ± 0.023 | 0.0165 ± 0.00029 | 0.0236 ± 0.00293 | 0.0111 ± 0.00133 | 0.0120 ± 0.00083 | 0.0112 |
| N820 | 4.68 ± 0.126 | 4.17 ± 0.037 | 3.57 ± 0.085 | 3.92 ± 0.187 | 0.0247 ± 0.00161 | 0.0214 ± 0.00129 | 0.0221 ± 0.00174 | 0.0174 ± 0.00186 | 0.0075 |
| N825 | 5.65 ± 0.386 | 3.70 ± 0.340 | 3.94 ± 0.232 | 4.21 ± 0.037 | 0.0236 ± 0.00120 | 0.0171 ± 0.00086 | 0.0118 ± 0.00181 | 0.0127 ± 0.00188 | 0.0197 |
| N851 | 4.24 ± 0.143 | 4.09 ± 0.093 | 3.39 ± 0.032 | 3.51 ± 0.169 | 0.0173 ± 0.00099 | 0.0160 ± 0.00050 | 0.0190 ± 0.00013 | 0.0186 ± 0.00012 | 0.0077 |
| N858 | 4.89 ± 0.192 | 4.36 ± 0.047 | 4.28 ± 0.309 | 3.62 ± 0.188 | 0.0245 ± 0.00136 | 0.0171 ± 0.00152 | 0.0155 ± 0.00326 | 0.0162 ± 0.00000 | 0.0059 |
| N880 | 4.45 ± 0.088 | 4.80 ± 0.076 | 3.12 ± 0.051 | 3.53 ± 0.182 | 0.0187 ± 0.00056 | 0.0162 ± 0.00109 | 0.0201 ± 0.00013 | 0.0110 ± 0.00181 | 0.0173 |
| C271 | 4.90 ± 0.078 | 5.22 ± 0.243 | 3.00 ± 0.057 | 3.39 ± 0.101 | 0.0228 ± 0.00126 | 0.0250 ± 0.00086 | 0.0214 ± 0.00192 | 0.0230 ± 0.00081 | 0.0041 |
| C272 | 3.36 ± 0.170 | 4.19 ± 0.191 | 3.20 ± 0.103 | 3.45 ± 0.281 | 0.0107 ± 0.00106 | 0.0202 ± 0.00011 | 0.0224 ± 0.00028 | 0.0158 ± 0.00167 | 0.0076 |
| C278 | 3.96 ± 0.014 | 4.17 ± 0.076 | 3.14 ± 0.193 | 3.32 ± 0.124 | 0.0199 ± 0.00046 | 0.0199 ± 0.00150 | 0.0197 ± 0.00109 | 0.0192 ± 0.00039 | 0.0054 |
| C282 | 3.60 ± 0.067 | 4.70 ± 0.058 | 3.36 ± 0.132 | 3.56 ± 0.062 | 0.0205 ± 0.00151 | 0.0189 ± 0.00002 | 0.0110 ± 0.00094 | 0.0173 ± 0.00046 | 0.0082 |
| C293 | 4.02 ± 0.147 | 4.71 ± 0.099 | 3.08 ± 0.294 | 3.45 ± 0.038 | 0.0211 ± 0.00126 | 0.0148 ± 0.00134 | 0.0212 ± 0.00568 | 0.0183 ± 0.00084 | 0.0093 |
| C299 | 4.23 ± 0.048 | 4.48 ± 0.041 | 3.81 ± 0.030 | 3.72 ± 0.150 | 0.0245 ± 0.00088 | 0.0167 ± 0.00046 | 0.0250 ± 0.00154 | 0.0190 ± 0.00163 | 0.0091 |
| C310 | 3.14 ± 0.346 | 4.60 ± 0.158 | 3.99 ± 0.156 | 3.24 ± 0.110 | 0.0215 ± 0.00189 | 0.0231 ± 0.00302 | 0.0235 ± 0.00166 | 0.0220 ± 0.00205 | 0.0081 |
| C312 | 3.37 ± 0.194 | 4.19 ± 0.301 | 2.82 ± 0.124 | 2.38 ± 0.153 | 0.0209 ± 0.00006 | 0.0185 ± 0.00062 | 0.0240 ± 0.00163 | 0.0261 ± 0.00169 | 0.0057 |
| C335 | 3.82 ± 0.101 | 3.16 ± 0.003 | 3.34 ± 0.286 | 3.78 ± 0.327 | 0.0165 ± 0.00015 | 0.0177 ± 0.00051 | 0.0094 ± 0.00104 | 0.0226 ± 0.00024 | 0.0071 |
| C361 | 3.71 ± 0.060 | 3.18 ± 0.114 | 3.17 ± 0.155 | 2.85 ± 0.226 | 0.0205 ± 0.00171 | 0.0220 ± 0.00082 | 0.0215 ± 0.00068 | 0.0172 ± 0.00091 | 0.0038 |
| C364 | 4.44 ± 0.113 | 3.52 ± 0.060 | 3.24 ± 0.167 | 3.18 ± 0.105 | 0.0241 ± 0.00037 | 0.0190 ± 0.00568 | 0.0233 ± 0.00116 | 0.0251 ± 0.00138 | 0.0108 |
| C372 | 3.77 ± 0.182 | 3.86 ± 0.294 | 3.22 ± 0.278 | 3.64 ± 0.017 | 0.0175 ± 0.00001 | 0.0187 ± 0.00138 | 0.0179 ± 0.00087 | 0.0174 ± 0.00086 | 0.0122 |
| C386 | 3.63 ± 0.373 | 3.87 ± 0.352 | 3.08 ± 0.188 | 3.41 ± 0.359 | 0.0213 ± 0.00133 | 0.0153 ± 0.00376 | 0.0207 ± 0.00099 | 0.0279 ± 0.00085 | 0.0047 |
| C401 | 4.03 ± 0.035 | 2.26 ± 0.188 | 3.91 ± 0.044 | 3.93 ± 0.357 | 0.0190 ± 0.00187 | 0.0178 ± 0.00093 | 0.0162 ± 0.00129 | 0.0186 ± 0.00127 | 0.0062 |
| C414 | 3.56 ± 0.021 | 4.93 ± 0.011 | 3.16 ± 0.130 | 3.38 ± 0.194 | 0.0151 ± 0.00098 | 0.0168 ± 0.00158 | 0.0252 ± 0.00104 | 0.0242 ± 0.00003 | 0.0102 |
| C447 | 4.14 ± 0.109 | 3.72 ± 0.021 | 3.18 ± 0.063 | 3.37 ± 0.030 | 0.0210 ± 0.00025 | 0.0267 ± 0.00058 | 0.0282 ± 0.00010 | 0.0219 ± 0.00151 | 0.0066 |
| C457 | 3.37 ± 0.121 | 4.12 ± 0.113 | 3.13 ± 0.233 | 3.16 ± 0.263 | 0.0190 ± 0.00158 | 0.0217 ± 0.00053 | 0.0110 ± 0.00087 | 0.0202 ± 0.00024 | 0.0066 |
| C482 | 3.75 ± 0.143 | 3.70 ± 0.076 | 3.33 ± 0.195 | 3.19 ± 0.129 | 0.0247 ± 0.00067 | 0.0198 ± 0.00198 | 0.0223 ± 0.00092 | 0.0186 ± 0.00096 | 0.0058 |
| C494 | 3.16 ± 0.145 | 5.31 ± 0.005 | 2.99 ± 0.006 | 2.50 ± 0.181 | 0.0159 ± 0.00048 | 0.0149 ± 0.00163 | 0.0142 ± 0.00014 | 0.0151 ± 0.00106 | 0.0044 |
| C504 | 1.82 ± 0.245 | 4.40 ± 0.189 | 3.37 ± 0.103 | 2.12 ± 0.105 | 0.0111 ± 0.00075 | 0.0214 ± 0.00007 | 0.0140 ± 0.00026 | 0.0132 ± 0.00104 | 0.0033 |
| C540 | 3.28 ± 0.008 | 4.41 ± 0.191 | 3.33 ± 0.164 | 3.16 ± 0.227 | 0.0199 ± 0.00035 | 0.0184 ± 0.00162 | 0.0154 ± 0.00015 | 0.0217 ± 0.00059 | 0.0049 |
| C557 | 3.57 ± 0.010 | 5.10 ± 0.052 | 3.49 ± 0.218 | 3.36 ± 0.172 | 0.0189 ± 0.00167 | 0.0178 ± 0.00131 | 0.0122 ± 0.00187 | 0.0209 ± 0.00108 | 0.0070 |
| C572 | 3.23 ± 0.076 | 3.15 ± 0.073 | 2.84 ± 0.169 | 2.83 ± 0.222 | 0.0139 ± 0.00113 | 0.0159 ± 0.00082 | 0.0167 ± 0.00047 | 0.0207 ± 0.00332 | 0.0090 |
| C583 | 2.24 ± 0.155 | 3.80 ± 0.081 | 2.03 ± 0.174 | 2.47 ± 0.063 | 0.0218 ± 0.00063 | 0.0127 ± 0.00040 | 0.0159 ± 0.00072 | 0.0203 ± 0.00016 | 0.0023 |
| C587 | 4.66 ± 0.229 | 3.06 ± 0.130 | 3.17 ± 0.033 | 3.34 ± 0.248 | 0.0172 ± 0.00197 | 0.0176 ± 0.00168 | 0.0261 ± 0.00061 | 0.0159 ± 0.00183 | 0.0065 |
| C589 | 2.74 ± 0.175 | 3.09 ± 0.222 | 2.36 ± 0.256 | 2.58 ± 0.050 | 0.0270 ± 0.00030 | 0.0193 ± 0.00010 | 0.0224 ± 0.00054 | 0.0242 ± 0.00028 | 0.0031 |
| C658 | 2.82 ± 0.026 | 2.92 ± 0.267 | 2.43 ± 0.268 | 3.16 ± 0.187 | 0.0233 ± 0.00112 | 0.0216 ± 0.00176 | 0.0091 ± 0.00032 | 0.0219 ± 0.00130 | 0.0019 |
| C733 | 5.52 ± 0.179 | 4.46 ± 0.053 | 3.17 ± 0.146 | 3.42 ± 0.089 | 0.0157 ± 0.00413 | 0.0221 ± 0.00156 | 0.0309 ± 0.00027 | 0.0148 ± 0.00073 | 0.0110 |
| C743 | 1.91 ± 0.174 | 3.90 ± 0.135 | 3.24 ± 0.182 | 2.94 ± 0.205 | 0.0169 ± 0.00057 | 0.0201 ± 0.00015 | 0.0179 ± 0.00122 | 0.0133 ± 0.00188 | 0.0012 |
| C757 | 3.52 ± 0.136 | 4.22 ± 0.035 | 3.48 ± 0.191 | 2.97 ± 0.329 | 0.0154 ± 0.00032 | 0.0229 ± 0.00016 | 0.0211 ± 0.00024 | 0.0107 ± 0.00123 | 0.0058 |
| C771 | 4.05 ± 0.319 | 4.13 ± 0.258 | 3.15 ± 0.199 | 3.10 ± 0.053 | 0.0176 ± 0.00101 | 0.0194 ± 0.00035 | 0.0248 ± 0.00132 | 0.0157 ± 0.00244 | 0.0053 |
| C826 | 3.31 ± 0.059 | 4.25 ± 0.263 | 2.95 ± 0.019 | 4.39 ± 0.010 | 0.0161 ± 0.00044 | 0.0189 ± 0.00172 | 0.0099 ± 0.00180 | 0.0218 ± 0.00041 | 0.0058 |
| C856 | 3.85 ± 0.196 | 4.18 ± 0.082 | 3.46 ± 0.259 | 3.64 ± 0.125 | 0.0185 ± 0.00108 | 0.0157 ± 0.00160 | 0.0195 ± 0.00129 | 0.0215 ± 0.00012 | 0.0051 |
| C939 | 3.81 ± 0.228 | 3.62 ± 0.080 | 3.41 ± 0.122 | 3.39 ± 0.110 | 0.0284 ± 0.00049 | 0.0207 ± 0.00017 | 0.0158 ± 0.00121 | 0.0227 ± 0.00024 | 0.0043 |
| C981 | 4.41 ± 0.058 | 4.56 ± 0.098 | 3.11 ± 0.165 | 3.21 ± 0.384 | 0.0264 ± 0.00132 | 0.0228 ± 0.00184 | 0.0215 ± 0.00069 | 0.0106 ± 0.00039 | 0.0104 |
| C990 | 4.28 ± 0.018 | 4.76 ± 0.144 | 2.61 ± 0.232 | 3.15 ± 0.274 | 0.0175 ± 0.00061 | 0.0180 ± 0.00070 | 0.0216 ± 0.00024 | 0.0219 ± 0.00165 | 0.0090 |
| C993 | 4.57 ± 0.068 | 4.44 ± 0.034 | 2.48 ± 0.012 | 2.93 ± 0.156 | 0.0144 ± 0.00072 | 0.0120 ± 0.00144 | 0.0213 ± 0.00327 | 0.0121 ± 0.00196 | 0.0051 |
| C997 | 3.17 ± 0.208 | 4.33 ± 0.126 | 2.28 ± 0.020 | 2.56 ± 0.107 | 0.0264 ± 0.00105 | 0.0185 ± 0.00106 | 0.0204 ± 0.00011 | 0.0124 ± 0.00074 | 0.0058 |
| C1024 | 5.01 ± 0.012 | 5.62 ± 0.037 | 3.70 ± 0.027 | 2.99 ± 0.001 | 0.0103 ± 0.00512 | 0.0295 ± 0.00118 | 0.0242 ± 0.0009 | 0.0091 ± 0.00053 | 0.0125 |
| C1099 | 3.23 ± 0.143 | 4.42 ± 0.121 | 2.70 ± 0.181 | 2.85 ± 0.230 | 0.0253 ± 0.00038 | 0.0207 ± 0.00045 | 0.0193 ± 0.00173 | 0.0121 ± 0.00032 | 0.0028 |
| C1114 | 3.10 ± 0.292 | 4.79 ± 0.182 | 2.63 ± 0.235 | 3.11 ± 0.118 | 0.0069 ± 0.00138 | 0.0232 ± 0.00193 | 0.0203 ± 0.00020 | 0.0114 ± 0.00036 | 0.0050 |
| C1138 | 2.9 ± 0.159 | 4.49 ± 0.080 | 2.08 ± 0.315 | 2.51 ± 0.323 | 0.0203 ± 0.00178 | 0.0288 ± 0.00054 | 0.0251 ± 0.00093 | 0.0135 ± 0.00054 | 0.0045 |
| C1153 | 3.67 ± 0.297 | 5.10 ± 0.187 | 2.70 ± 0.151 | 2.39 ± 0.092 | 0.0121 ± 0.00163 | 0.0196 ± 0.00174 | 0.0245 ± 0.00022 | 0.0082 ± 0.00141 | 0.0031 |
| C1189 | 4.87 ± 0.129 | 4.24 ± 0.269 | 4.08 ± 0.006 | 3.61 ± 0.257 | 0.0117 ± 0.00031 | 0.0183 ± 0.00257 | 0.0095 ± 0.00447 | 0.0122 ± 0.00355 | 0.0072 |
| C1233 | 3.92 ± 0.018 | 4.86 ± 0.016 | 3.10 ± 0.043 | 3.08 ± 0.031 | 0.0237 ± 0.00064 | 0.0175 ± 0.00285 | 0.0235 ± 0.00115 | 0.0201 ± 0.00016 | 0.0050 |
| C1257 | 4.72 ± 0.243 | 5.07 ± 0.374 | 4.47 ± 0.225 | 3.24 ± 0.192 | 0.0266 ± 0.00563 | 0.0137 ± 0.00085 | 0.0130 ± 0.00064 | 0.0235 ± 0.00171 | 0.0153 |
| C1337 | 3.99 ± 0.040 | 5.39 ± 0.135 | 2.36 ± 0.232 | 3.77 ± 0.126 | 0.0162 ± 0.00098 | 0.0163 ± 0.00071 | 0.0196 ± 0.00340 | 0.0136 ± 0.00105 | 0.0028 |
| C1338 | 4.27 ± 0.153 | 4.79 ± 0.100 | 2.49 ± 0.219 | 2.94 ± 0.131 | 0.0190 ± 0.00116 | 0.0219 ± 0.00046 | 0.0137 ± 0.00325 | 0.0207 ± 0.00200 | 0.0065 |
| C1341 | 4.29 ± 0.093 | 4.37 ± 0.175 | 3.71 ± 0.135 | 3.41 ± 0.281 | 0.0223 ± 0.00006 | 0.0235 ± 0.00048 | 0.0102 ± 0.00058 | 0.0195 ± 0.00173 | 0.0064 |
| C1381 | 4.76 ± 0.279 | 5.81 ± 0.080 | 3.73 ± 0.083 | 3.10 ± 0.179 | 0.0193 ± 0.00057 | 0.0173 ± 0.00054 | 0.0065 ± 0.00049 | 0.0219 ± 0.00145 | 0.0075 |

PBL, peripheral blood leukocyte; NEU, neutrophil; LYM, lymphocyte; MONO, monocyte.

**Table S6.** The qualitative and quantitative ions for the detection of nucleosides.

| Analytes | Charge state | Qualitative ions (m/z) | Quantitative ion (m/z) |
| --- | --- | --- | --- |
| **A** | 1 | 268.103, 136.061 | 136.061 |
| **U** | 1 | 245.079, 113.036 | 113.036 |
| **C** | 1 | 244.092, 112.050 | 112.05 |
| **G** | 1 | 284.098, 152.065 | 152.065 |
| **dA** | 1 | 252.110, 136.061 | 136.061 |
| **T** | 1 | 243.098, 127.051 | 127.051 |
| **dC** | 1 | 228.098, 112.050 | 112.05 |
| **dG** | 1 | 268.105, 152.065 | 152.065 |
| **5-mdC** | 1 | 242.114, 126.068 | 126.068 |
| **5-hmdC** | 1 | 258.109, 142.062 | 142.062 |

**Table S7.** Accuracy of the method for the detection of 5-mdC and 5-hmdC.

| QCs | Nominal | Observed | RSD % (n=3) | Relative error (%) |
| --- | --- | --- | --- | --- |
| 5-mdC (vs. [dC], %) | 1.00 | 1.11 | 6.7 | 11.0 |
|  | 3.00 | 3.16 | 4.4 | 5.3 |
|  | 6.00 | 6.28 | 2.9 | 4.7 |
| 5-hmdC (vs. [dC], %) | 0.0050 | 0.0055 | 13.2 | 10.0 |
|  | 0.0100 | 0.0095 | 6.3 | -5.0 |
|  | 0.0500 | 0.0519 | 4.0 | 3.8 |
| RSD, relative standard deviation. | | | | |

**Table S8.** Intra- and inter-day imprecision for the quantification of 5-mdC and 5-hmdC by LC-ESI-MS method.

|  | Intra-day (RSD %, n = 3) | | | Inter-day (RSD %, n = 5) | | |
| --- | --- | --- | --- | --- | --- | --- |
| 5-mdC | 5-mdC/dC, 0.5% | 5-mdC/dC, 1.0% | 5-mdC/dC, 5.0% | 5-mdC/dC, 0.5% | 5-mdC/dC, 1.0% | 5-mdC/dC, 5.0% |
|  | 2.8 | 3.5 | 2.3 | 4.1 | 6.7 | 2.9 |
| 5-hmdC | 5-hmdC/dC, 0.01% | 5-hmdC/dC, 0.05% | 5-hmdC/dC, 0.10% | 5-hmdC/dC, 0.01% | 5-hmdC/dC, 0.05% | 5-hmdC/dC, 0.10% |
|  | 9.6 | 10.1 | 7.8 | 10.7 | 12.9 | 8.4 |

**Table S9.** Primers used for qPCR.

| Genes | Primers (5’→3’) | Amplicon length |
| --- | --- | --- |
| GAPDH | Forward: GAAGGTGAAGGTCGGAGTC | 226bp |
|  | Reverse: GAAGATGGTGATGGGATTTC |  |
| DNMT1 | Forward: ACCGCTTCTACTTCCTCGAGGCCTA | 335bp |
|  | Reverse: GTTGCAGTCCTCTGTGAACACTGTGG |  |
| DNMT3A | Forward: GACAAGAATGCCACCAAAGC | 190bp |
|  | Reverse: CGTCTCCGAACCACATGAC |  |
| DNMT3B | Forward: AATGTGAATCCAGCCAGGAAAGGC | 191bp |
|  | Reverse: ACTGGATTACACTCCAGGAACCGT |  |
| TET1 | Forward: TCTTCCCCATGACCACATCT | 105bp |
|  | Reverse: GAGGGAAAAGAAGCCCAAAG |  |
| TET2 | Forward: GGACATGATCCAGGAAGAGC | 200bp |
|  | Reverse: GCTTCCATTCTGGAGCTTTG |  |
| TET3 | Forward: CCCACAAGGACCAGCATAAC | 129bp |
|  | Reverse: CCATCTTGTACAGGGGGAGA |  |
| Primers were designed to target the location of exon/intron junction to avoid the amplification of contaminated DNA. | | |

**Figure S1.** The mRNA expression for DNMT1, DNMT3A, DNMT3B, TET1, TET2, and TET3 in the subgroup.


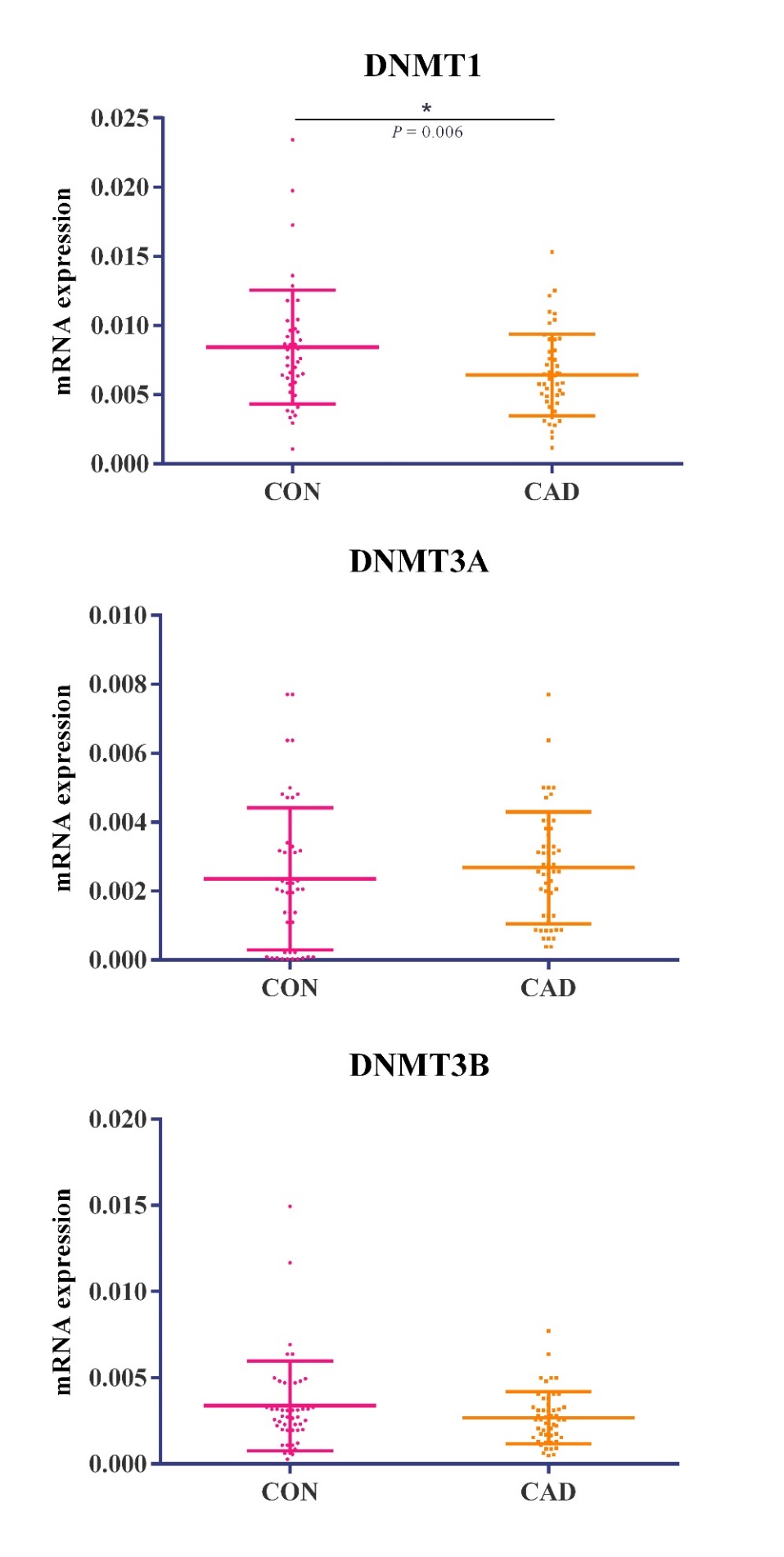


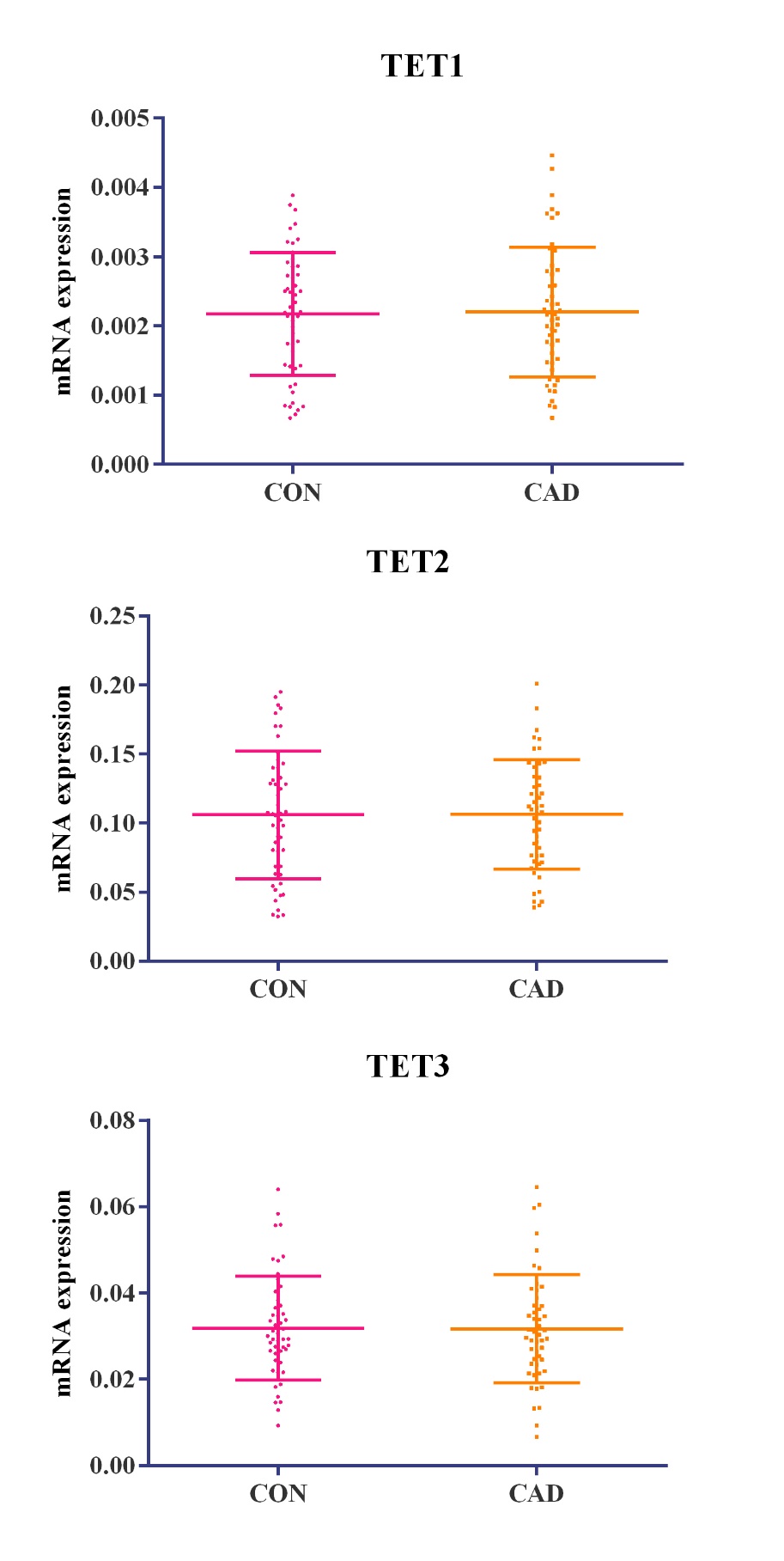


**Figure S2.** Confirmation of the isolated monocytes with FACS.


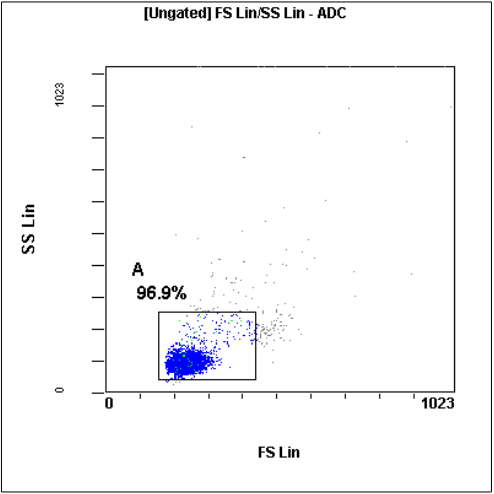

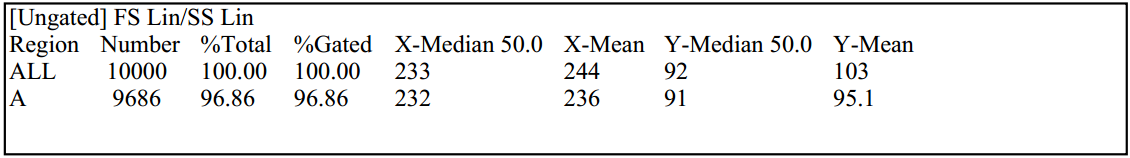

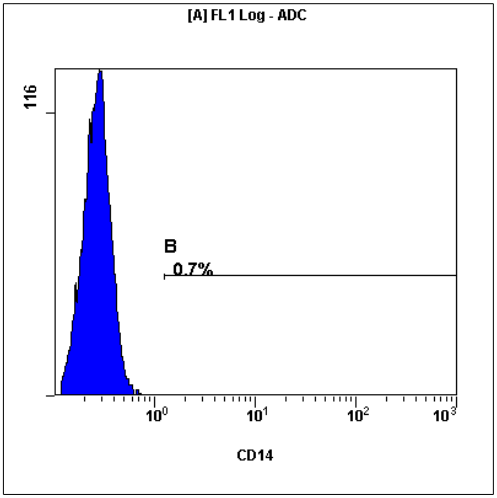

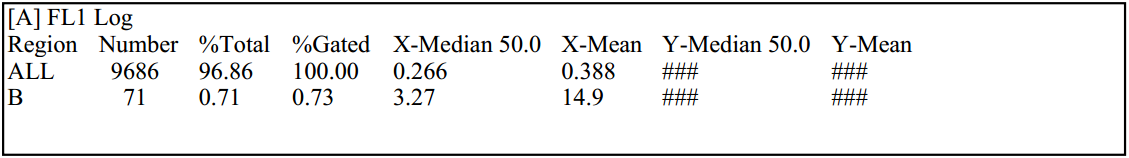


**Figure S3.** The standard curves, amplification plots and melting peaks of qPCR.

*
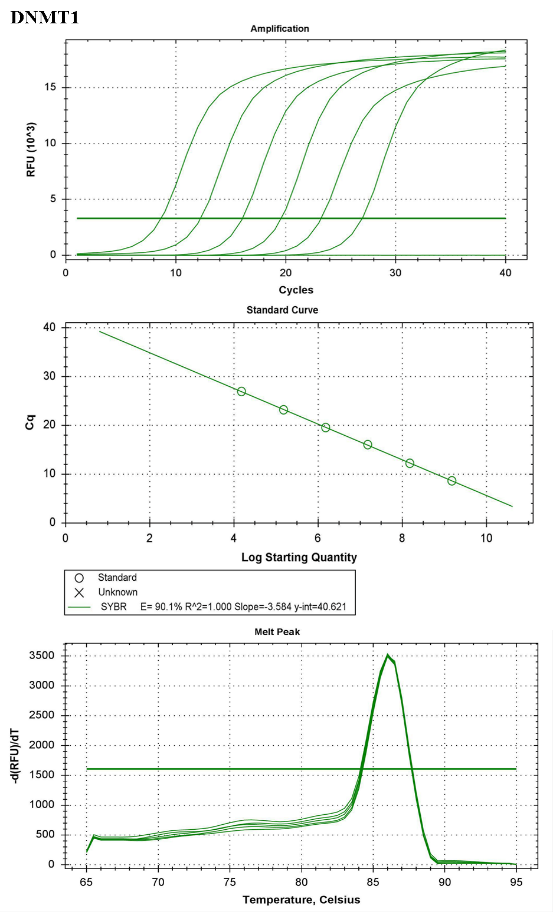
*

*
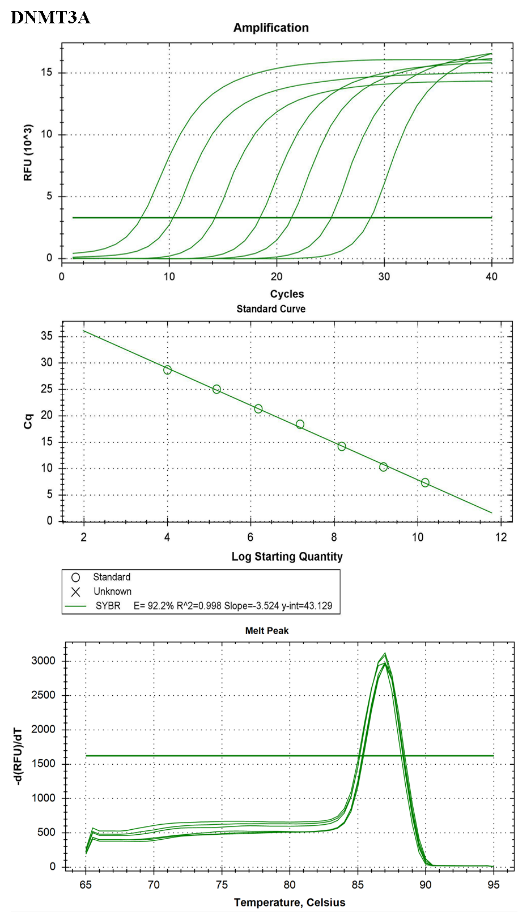
*

*
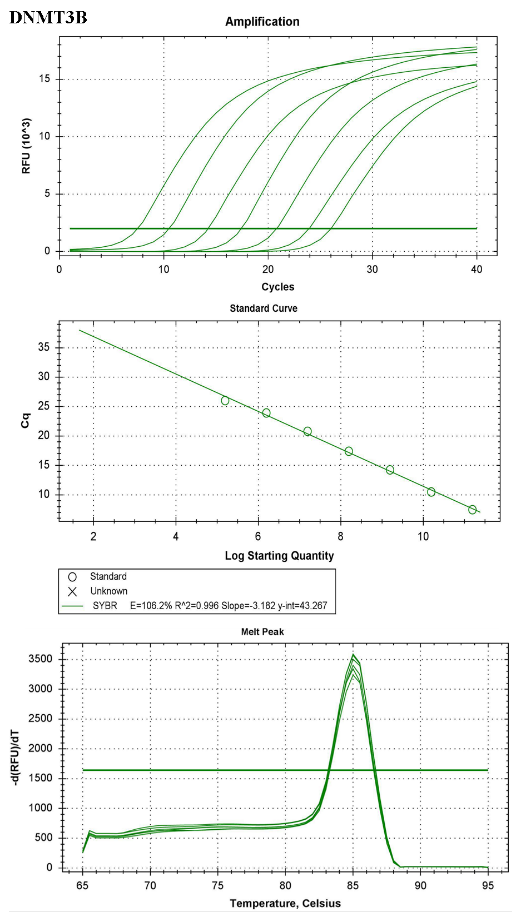
*


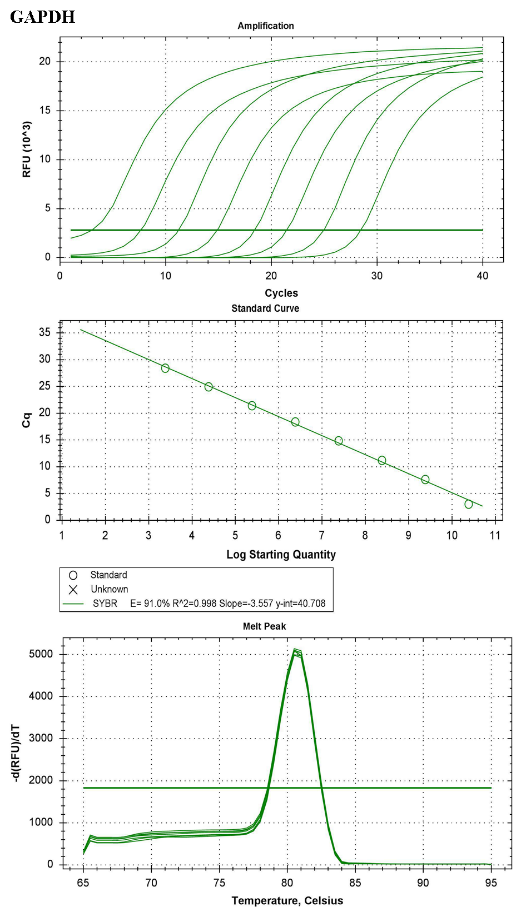


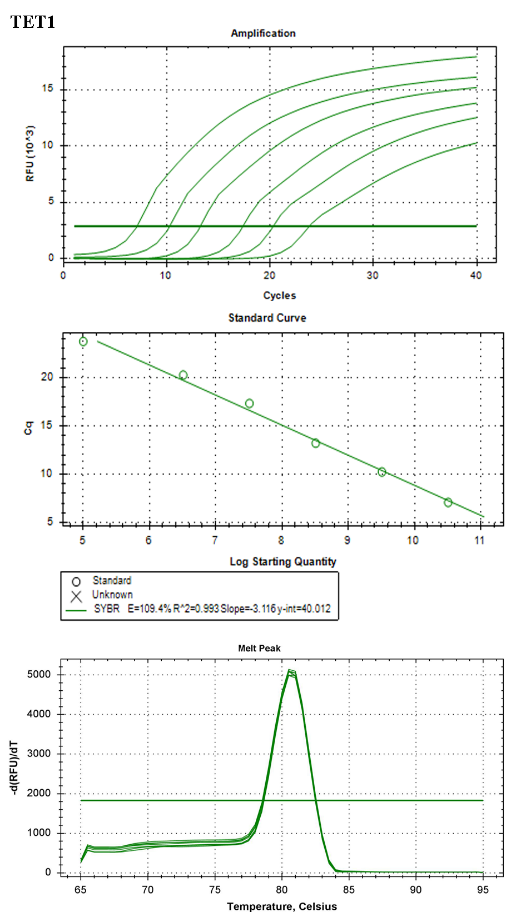


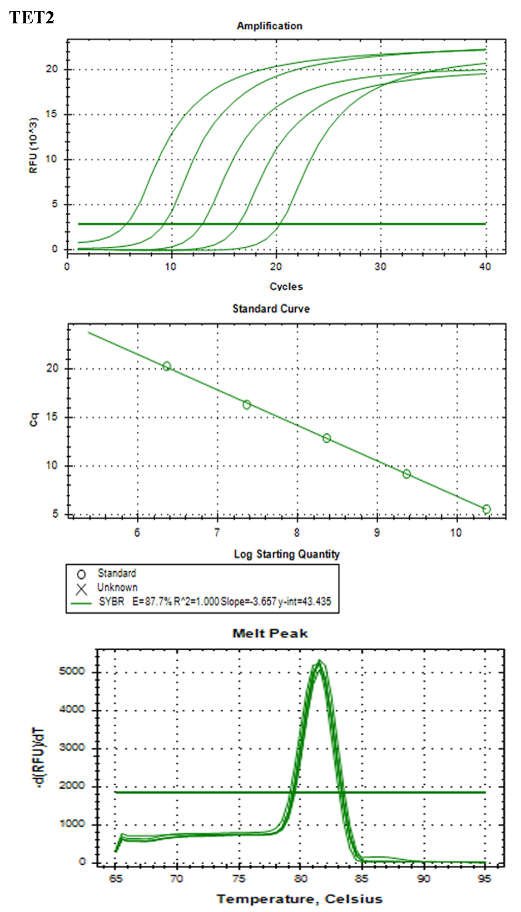


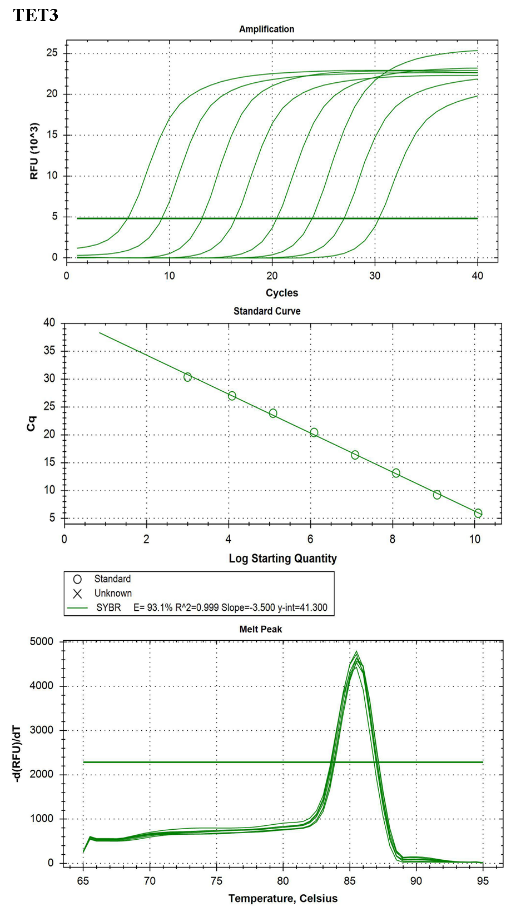

Supplement: Additional file 1: Table S1. — Clinical characteristics and measure contents of 5-mdC in genomic DNA of blood from 220 healthy controls. Table S2. Clinical characteristics and measure contents of 5-mdC in genomic DNA of blood from 215 CAD patients. Table S3. Clinical characteristics of 50 healthy controls in the randomly selected subgroup. Table S4. Clinical characteristics of 50 CAD patients in the randomly selected subgroup. Table S5. Measured contents of 5-mdC and 5-hmdC in genomic DNA of blood samples in subgroup with different blood cell sources. Table S6. The qualitative and quantitative ions for the detection of nucleosides. Table S7. Accuracy of the method for the detection of 5-mdC and 5-hmdC. Table S8. Intra- and inter-day imprecision for the quantification of 5-mdC and 5-hmdC by LC-ESI-MS/MS method. Table S9. Primers used for qPCR. Figure S1. The mRNA expression for DNMT1, DNMT3A, DNMT3B, TET1, TET2, and TET3 in the subgroup. Figure S2. Confirmation of the isolated monocytes with FACS. Figure S3. The standard curves, amplification plots, and melting peaks of qPCR. (DOCX 2049 kb) [file 13148_2018_443_MOESM1_ESM.docx]
